# Identification of Pluripotent and Adult Stem Cell Genes Unrelated to Cell Cycle and Associated with Poor Prognosis in Multiple Myeloma

**DOI:** 10.1371/journal.pone.0042161

**Published:** 2012-07-31

**Authors:** Alboukadel Kassambara, Dirk Hose, Jérôme Moreaux, Thierry Rème, Jennifer Torrent, Jean François Rossi, Hartmut Goldschmidt, Bernard Klein

**Affiliations:** 1 CHU Montpellier, Institute of Research in Biotherapy, Montpellier, France; 2 INSERM, U1040, Montpellier, France; 3 Université MONTPELLIER1, UFR Médecine, Montpellier, France; 4 Medizinische Klinik V, Universitätsklinikum Heidelberg and Nationales Centrum für Tumorerkrankungen, Heidelberg, Germany; Texas A&M University, United States of America

## Abstract

Gene expression-based scores used to predict risk in cancer frequently include genes coding for DNA replication, repair or recombination. Using two independent cohorts of 206 and 345 previously-untreated patients with Multiple Myeloma (MM), we identified 50 cell cycle-unrelated genes overexpressed in multiple myeloma cells (MMCs) compared to normal human proliferating plasmablasts and non-proliferating bone marrow plasma cells and which have prognostic value for overall survival. Thirty-seven of these 50 myeloma genes (74%) were enriched in genes overexpressed in one of 3 normal human stem cell populations – pluripotent (18), hematopoietic (10) or mesenchymal stem cells (9) - and only three genes were enriched in one of 5 populations of differentiated cells (memory B lymphocytes, T lymphocytes, polymorphonuclear cells, monocytes, osteoclasts). These 37 genes shared by MMCs and adult or pluripotent stem cells were used to build a stem cell score (^SC^score), which proved to be strongly prognostic in the 2 independent cohorts of patients compared to other gene expression-based risk scores or usual clinical scores using multivariate Cox analysis. This finding highlights cell cycle-unrelated prognostic genes shared by myeloma cells and normal stem cells, whose products might be important for normal and malignant stem cell biology.

## Introduction

Multiple Myeloma (MM) is a molecularly heterogeneous disease with recurrent gene translocations, deletions or gains and changes in gene expression in Multiple Myeloma Cells (MMCs) [1], [2]. High throughput DNA microarrays made it possible to identify gene-expression in MMCs linked with event free and/or overall survival of previously-untreated patients. These includes the high risk score from the University for Medical Sciences of Arkansas (UAMS-HRS) [3], the intergroupe Francophone du Myélome (IFM) score [4], and surrogate markers of proliferation [5], [6]. These scores mainly include genes coding for proteins involved in cell cycle, metabolism, and cell communication. We are interested in identifying genes whose gene products are unrelated to the machinery used for DNA replication and whose expression could predict for risk in patients with previously-untreated MM. In particular, it is of interest to investigate whether MMCs could aberrantly express genes shared by their bone environment, which could confer to them the ability to become less dependent on the environment and eventually to metastasize [7]. It is also of interest to investigate whether MMCs could express genes related to normal stem cell populations. Several studies have emphasized the existence of a Myeloma Stem cell, which lacked CD138, was able to form colonies in semi-solid medium in vitro, and recapitulate the tumor growth in vivo [8]. These studies were not confirmed, weakening the stem cell hypothesis in Multiple Myeloma [9]. But in order to get a tumor growth, it is mandatory that some MMCs have the stem cell property of self-renewal in vivo, in particular to recapitulate the tumor occurring after drug exposure. Whether all MMCs have this stem cell potential, which could be waked up given appropriate environment conditions or whether there is a fixed differentiation hierarchy within the myeloma tumor in vivo is an open issue. The recent finding that a few genes could reprogram a human adult cell into pluripotent stem cells favors such plasticity to occur in vivo [10]. In particular, the four Yamanaka pluripotent genes – *OCT4, SOX2, KLF4 and MYC* – are expressed by human pancreatic cancer cells in vivo [11]. Of note, we and others have shown that 3 of 4 Yamanaka genes – *KLF4, SOX2 and MYC* - are expressed by MMCs [12], suggesting MMCs could dedifferentiation in vivo.

To delete cell cycle genes, we took advantage of the possibility to generate high numbers of highly cell cycling normal plasmablasts, starting from human peripheral blood memory B lymphocytes [13], [14]. Fifty cell-cycle unrelated genes over- or under-expressed in MMCs were thus identified, whose expression is associated with overall survival in 2 independent large cohorts of patients with previously untreated MM. Of major interest, 37 (74%) of these 50 cell-cycle unrelated prognostic myeloma genes are overexpressed in 3 normal stem cell populations - pluripotent, hematopoietic or mesenchymal stem cells - compared to normal differentiated cells. In addition, these 37 cell-cycle unrelated genes make it possible to build a powerful prognostic “stem cell” score.

## Results

### Cell Cycle Unrelated Genes Overexpressed in Primary Myeloma Cells and/or Myeloma Cell Lines Compared to Normal Plasmablasts or Plasma Cells and Predicting Patients’ Overall Survival

**Figure 1:**
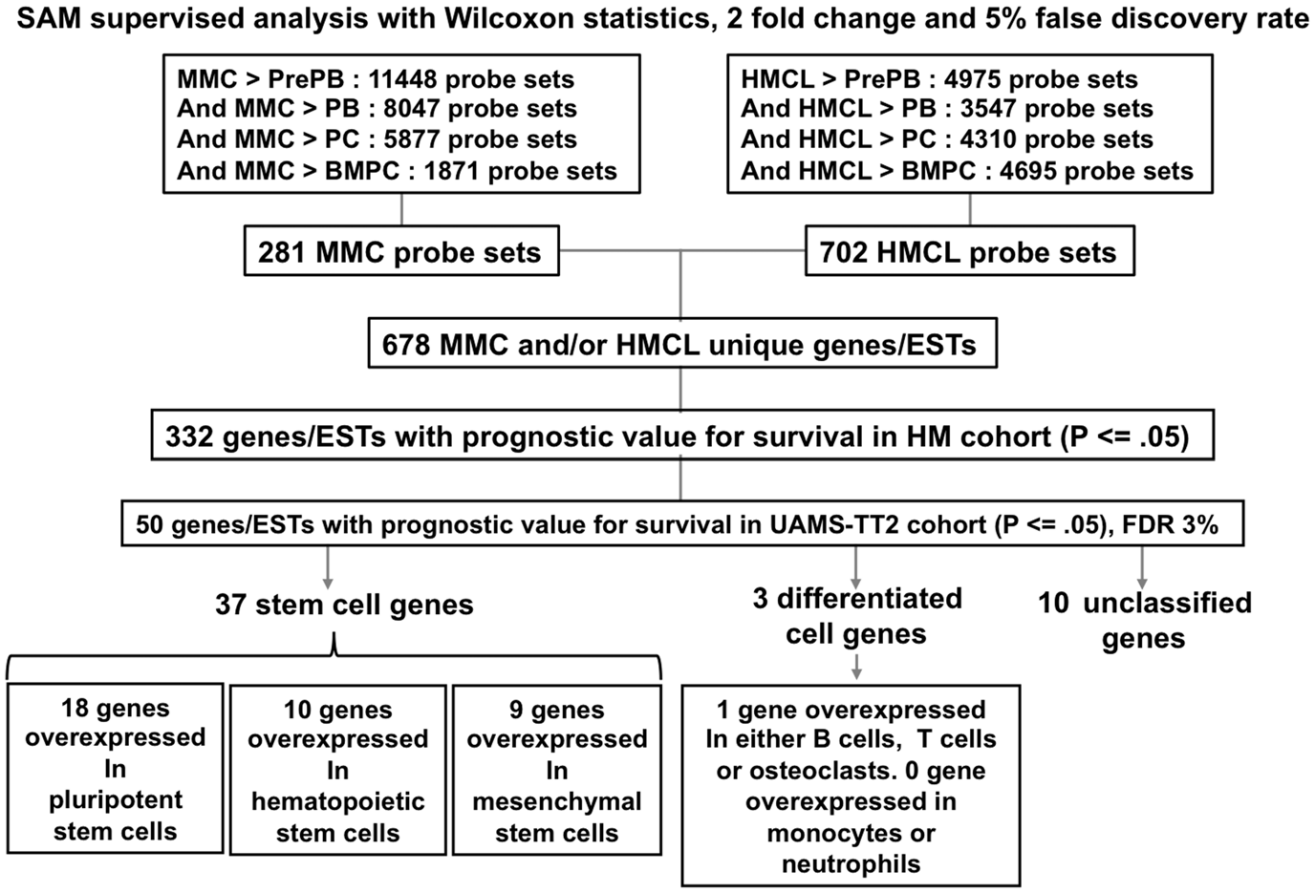
identification of genes overexpressed in primary myeloma cells and/or myeloma cell lines compared to normal plasmablasts or plasma cells and associated with patients’ prognostic value. 281 and 702 probe sets were overexpressed in MMCs and HMCLs compared to normal counterparts, respectively. These probe sets correspond to 678 unique genes/ESTs among which 332 genes/ESTs were associated to bad or good prognostic using HM cohort of patients. The prognostic value of 50 out of the 332 genes/ESTs is validated using an independent UAMS-TT2 cohort. Among these 50 genes, 37 were stem cell genes, 3 were differentiated cell genes and 10 genes were unclassified.

As shown in Figure 1, GEPs of primary MMCs or HMCLs were compared to those of their highly cycling normal counterparts – in vitro generated preplasmablasts and plasmablasts - as well as to those of non cell cycling normal counterparts - in vitro generated plasma cells and normal bone marrow plasma cells - using SAM supervised analysis (1000 permutations, FDR ≤5%, ratio ≥2). Preplasmablasts and plasmablasts were used to delete genes coding for cell cycle machinery. 885 unique probe sets, coding for 678 unique myeloma genes or ESTs were overexpressed in primary MMCs (281 probe sets) or in HMCLs (702 probe sets) compared to normal counterparts (Supplementary Table S2). Using the R MaxStat function, 332 of the 678 genes had prognostic value in the HM cohort. To correct for multiple testing, the independent patient cohort of 345 patients treated with TT2 protocol (UAMS-TT2 cohort) was used. Fifty (43 bad and 7 good prognostic genes) of these 332 prognostic genes kept prognostic value in this independent cohort using MaxStat parameters designed in HM cohort, yielding to a 3% false discovery rate (Supplementary Table S3). As expected, these 50 genes did not encode for cell cycle related proteins.

### Cell-cycle Unrelated Prognostic Myeloma Genes are Highly Enriched in Pluripotent or Adult Stem Cell Genes

**Figure 2:**
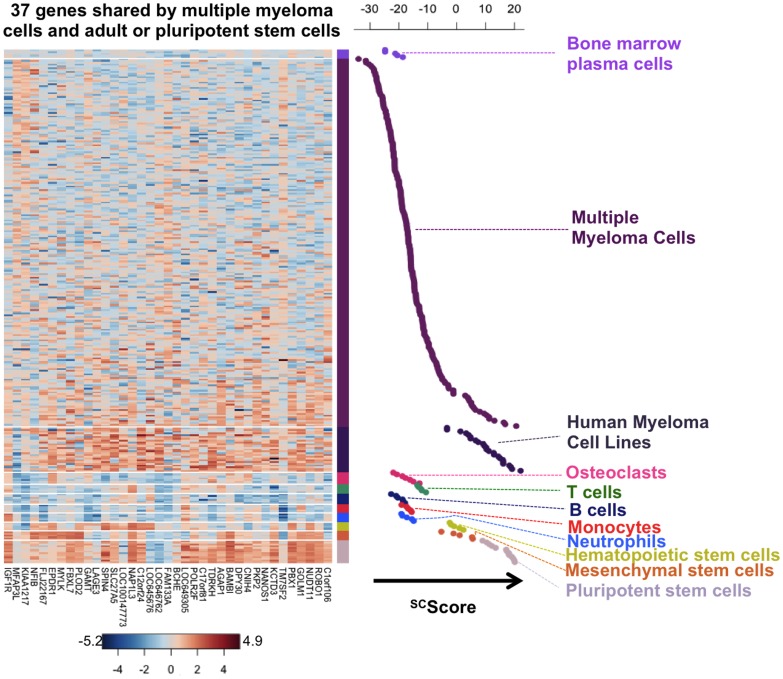
Expression profile of the 37 genes shared by multiple myeloma cells, adult or pluripotent stem cells. A heatmap of the expression of these genes is shown for normal bone marrow plasma cells, primary multiple myeloma cells from patients, myeloma cell lines, 5 populations of differentiated cells (B cells, T cells, neutrophils, monocytes and osteoclasts) and 3 human stem cell populations (pluripotent stem cells, hematopoietic stem cells and mesenchymal stem cells). Each value represents the difference from the gene median across normal and malignant samples and is depicted according to the color scale shown at the bottom (−5.2 to 4.9 on a log base 2 scale).

Since prognostic myeloma genes did not encode for cell-cycle related proteins, we investigated normal cell populations, which could express some of these myeloma genes specifically, using SAM multiclass analysis (1000 permutations, FDR <5%). Three human stem cell populations were included – pluripotent stem cells, hematopoietic stem cells and mesenchymal stem cells – and 5 populations of differentiated cells: B lymphocytes, T lymphocytes, polymorphonuclear cells, monocytes and osteoclasts. Considering genes overexpressed in only one normal population compared to others, 74% (37/50) of these prognostic myeloma genes were expressed by one of the 3 stem cell populations specifically and 6% (3/50) by differentiated cells (Table 1 and Figure 1). Ten genes were not specifically overexpressed in one of these 8 normal cell populations. A heatmap representation of the expression profile of the 37 stem cell genes in the normal and malignant cell populations as well as in stem cell and differentiated cell populations is displayed in Figure 2. Out of the 37 genes shared by MMCs and adult or pluripotent stem cells, 18 are overexpressed by pluripotent stem cells, 10 by hematopoietic stem cells, and 9 by mesenchymal stem cells (Table 1). This observation emphasizes that the cell-cycle unrelated prognostic myeloma genes are enriched in normal stem cell genes and we will focus in the following on these 37 stem cell genes. These 37 genes were found to be significantly associated with 5 pathways using Ingenuity: “nervous system development and Function, cellular development, genetic disorder”, “lipid and nucleic acid metabolism, molecular transport”, “tissue morphology, genetic disorder”, “endocrine system disorders”, and “hematological disease”. The 37 genes shared by MMCs and adult or pluripotent stem cells are variably expressed in HMCLs (supplementary Figure S1).

**Table 1:** Cell cycle unrelated prognostic myeloma genes are highly enriched in pluripotent or adult stem cell genes.

Pluripotent stem cells (n = 18)	Hematopoietic stem cells (n = 10)	Mesenchymal stem cells (n = 9)	Osteoclasts (n = 1)
*NUDT11*	*NAP1L3*	*EPDR1*	*CTSC*
*C1orf106*	*SPIN4*	*IGF1R*	
*PBX1*	*LOC100147773*	*MYLK*	
*NANOS1*	*LAGE3*	*PLOD2*	**T lymphocytes (n = 1)**
*ROBO1*	*SLC27A5*	*FBXL7*	ACVR1C*
*DPY30*	*LOC645676*	*FLJ22167*	
*AGAP1*	*FAM133A*	NFIB*	
*POLR2F*	*LOC646762*	MFAP3L*	**B lymphocytes (n = 1)**
*BAMBI*	*C12orf24*	KIAA1217*	CIRBP*
*GOLM1*	GAMT*		
*PKP2*			
*C17orf81*			**Polymorphonuclear cells (n = 0)**
*TDRKH*			
*KCTD3*			
*LOC649305*			**Monocytes (n = 0)**
*BCHE*			
*CNIH4*			
TM7SF2*			

Out of the 50 prognostic MM genes, 37 are overexpressed in only one normal cell population compared to 7 others. These 8 normal cell populations include 3 stem cell populations– pluripotent stem cells, mesenchymal stem cells and hematopoietic stem cells – and 5 populations of differentiated cells: B lymphocytes, T lymphocytes, polymorphonuclear cells, monocytes and osteoclasts. Genes are ranked according to their prognostic value. (*) indicates good prognostic genes.

### Building a Stem Cell Score (^SC^score) for Predicting Overall Survival

**Figure 3:**
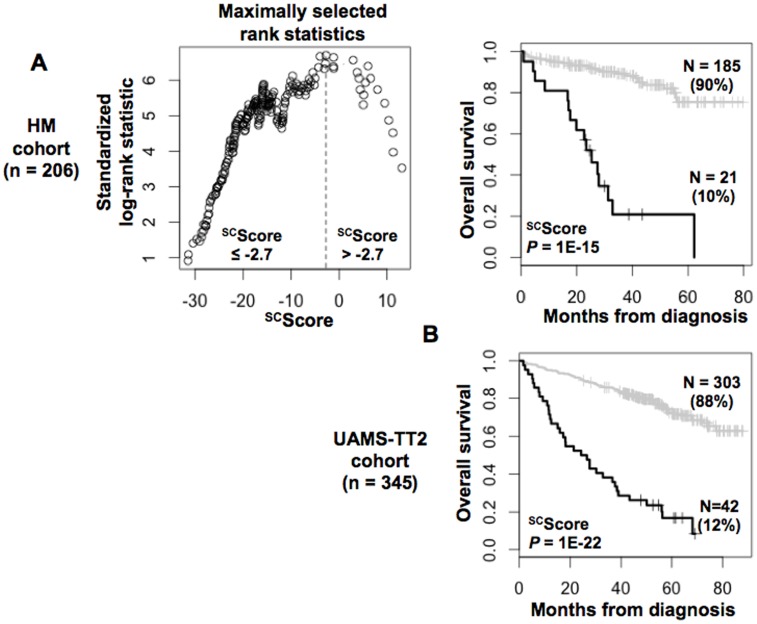
Building a myeloma-stem cell score (^SC^score) for predicting overall survival. A. The prognostic information provided by the 37 stem cell myeloma genes was summed within a ^SC^score as defined in the Materials and Methods. Patients of HM cohort were ranked according to increased ^SC^score and a maximum difference in OS was obtained with ^SC^score  = −2.7 splitting patients in a high risk (10%) and low risk (90%) groups. B. Validation of ^SC^score using UAMS-TT2 cohort.

The prognostic information provided by the 37 genes shared by MMCs and adult or pluripotent stem cells was summed within a ^SC^score as indicated in Materials and Methods. Based on this ^SC^score, Maxstat statistic test cuts the HM-patient cohort within 2 groups: a high-risk ^SC^score comprising 10% of patients with a median OAS of 25.3 months and a low-risk ^SC^score with a median OAS not-reached (*P* = 10^−15^, Figure 3A). ^SC^score was prognostic on HM cohort as a continuous variable. ^SC^score was also prognostic for the UAMS-TT2 cohort of 345 patients. Using the ^SC^score cut-point of −2.7 defined on HM cohort, 12% of UAMS-TT2 cohort patients were identified as high-risk ^SC^score group with a median OS of 25.5 months and 88% of UAMS-TT2 cohort patients were in low-risk ^SC^score group with a median OS not-reached (*P* = 1E-22, Figure 3B). ^SC^score was prognostic on UAMS-TT2 cohort as a continuous variable.

### Comparison of Various GEP-based Risk Scores

Three GEP-based risk scores were reported for MM disease using 70 genes for UAMS-HRS [3], 15 for IFM score [4] and 50 for a gene expression-based proliferation index (GPI) [5]. None of the 37 ^SC^score genes was used by IFM score and GPI, and only one (*ROBO1*) by UAMS-HRS. Prognostic value for overall survival of ^SC^score was compared with usual prognostic factors - ISS, spike *MMSET*, del17p - or the 3 GEP-based risk scores (UAMS-HRS, IFM score and GPI). Using univariate Cox analysis on HM cohort, all these factors had prognostic value and ^SC^score had the higher hazard ratio (Table 2A). Using multivariate Cox analysis, ^SC^score and GPI, kept prognostic value (Table 2C). Univariate cox analysis on UAMS-TT2 cohort showed that ^SC^score had the higher hazard ratio, followed by UAMS-HRS, del17p, spike *MMSET*, GPI and IFM score, ISS, β2m and albumin (Table 2A). Using multivariate analysis, ^SC^score, UAMS-HRS, GPI, del17p, ISS, β2m and albumin kept prognostic value (Table 2B).

**Table 2:** Cox univariate and multivariate analysis of OS in HM and UAMS-TT2 patients’ cohorts.

	Univariate Cox analysis - Overall survival			
A	HM		LR-TT2	
	HR	*P*	HR	*P*
^SC^Score	9	2.60E-11	6	3.50E-10
UAMS-HRS	2.4	1.40E-02	4.7	4.80E-13
IFM score	2.5	1.90E-02	1.8	4.00E-03
GPI	2.6	1.60E-04	1.8	2.20E-04
Spike MMSET	3.3	4.70E-04	2.2	3.20E-04
del17p	3.4	2.00E-02	2.5	3.70E-04
ISS	2	9.70E-04	1.6	0.000055
β2M	1.1	4.20E-05	1.1	0.000000049
Alb	0.47	1.40E-02	0.94	0.00012
	**Multivariate Cox analysis - Overall survival**			
**B**	**HM**		**LR-TT2**	
	**HR**	***P***	**HR**	**P**
^SC^Score	8.9	3.50E-09	4	2.60E-08
UAMS-HRS	1	NS	2.4	4.70E-04
^SC^Score	8.3	3.10E-10	5.7	3.40E-15
IFM score	1.7	NS	1.2	NS
^SC^Score	8.5	2.50E-10	5.4	7.90E-15
GPI	2.2	6.30E-04	1.4	3.60E-02
^SC^Score	13	4.20E-08	7.2	1.00E-13
Spike MMSET	0.62	NS	0.74	NS
^SC^Score	9.1	1.20E-10	5.9	0.00E+00
del17p	3.2	3.20E-02	2.3	1.30E-03
^SC^Score	7.6	1.80E-09	5.4	2.10E-15
ISS	1.6	1.60E-02	1.4	0.0033
^SC^Score	8	2.10E-09	5.1	2.20E-14
β2M	1	NS	1.1	0.0002
^SC^Score	8	8.70E-10	5.3	8.70E-15
Alb	0.65	NS	0.96	0.021
	**Multivariate Cox analysis - Overall survival**			
**C**	**HM**		**LR-TT2**	
	**HR**	**P**	**HR**	**P**
^SC^Score	6.4	0.0012	3.4	0.00013
del17p	1.9	NS	2.4	0.00093
ISS	1.4	NS	1.3	0.013
UAMS-HRS	0.68	NS	2.3	0.0078
IFM score	0.63	NS	0.9	NS
Spike MMSET	1.7	NS	1.1	NS
GPI	2.3	0.01	1.1	NS

**A**) Cox univariate analysis of overall survival. The prognostic factors were tested as single variable. **B**) Cox multivariate analysis of overall survival. The ^SC^Score were tested together with each of the prognostic factors. **C**) Cox multivariate analysis of overall survival using all prognostic factors together. Hazard ratios (HR) and *P*-values are shown. NS, Not Significant at a 5% threshold; GPI, gene expression based proliferation index; ISS, International Staging System; UAMS-HRS, high-risk score from UAMS; IFM, Intergroupe Francophone du Myelome.

### Link of ^SC^score with Patients’ Clinical and Genetic Parameters

The frequencies of patients with low albumin or low hemoglobin levels were significantly increased in patients with high risk ^SC^score (*P*≤.05, Supplementary Table S5). Others clinical data – age, β2m, ISS staging, CRP, Durie Salmon staging, light or heavy chain isotype and occurrence of bone lesions - were not significantly different between the 2 ^SC^score groups. The frequency of patients with t(4;14)(p16.3;q32.3), 1q21 or del13 was significantly increased in the high risk ^SC^score group. Of note, the frequencies of patients with del17p were not significantly different between the high-risk or low-risk ^SC^score groups (Table 3).

**Table 3:** Link of ^SC^score with patients’ Genetic abnormalities.

	^SC^Score ≤−2.7	^SC^Score >−2.7
t(11;14)^+^(n = 27)	**100%**	**0%**
t(11;14)^−^(n = 140)	**86%**	**14%**
t(4;14)^+^(n = 28)	***43%***	**57%**
t(4;14)^−^(n = 137)	***97%***	**3%**
1q21^+^(n = 62)	***79%***	**21%**
1q21^−^(n = 91)	***93%***	**7%**
del13^+^(n = 91)	***80%***	**20%**
del13^−^(n = 78)	***97%***	**3%**
del17^+^(n = 27)	78%	22%
del17^−^(n = 132)	90%	10%

Interphase-FISH-analysis was performed on CD138-purified plasma cells of 153 to 169 patients of the HM series, depending on the gene abnormality. Patients were separated in two groups according to ^SC^score (low-risk and high-risk groups). Data are the percentages of patients within these 2 groups with the biological parameters. When the percentages were different with a chisquare test (*P*≤.05), data are shown in bold and italic.

## Discussion

The major message of the current study is that prognostic genes aberrantly expressed by MMCs compared to normal plasmablasts or plasma cells and unrelated to DNA replication, repair and recombination are highly enriched (74%) in genes overexpressed in human pluripotent or adult stem cells. These 37 “stem cell myeloma genes” comprise 32 bad and 5 good prognostic genes and were used to build a stem cell risk score. The strong prognostic value of this stem cell risk score in 2 independent patients cohorts, compared to the two previously reported gene expression based risk score, emphasizes the biological relevance of the stem cell myeloma genes.

We can reasonably eliminate the possibility that the stem cell genes expressed by MMCs could be due to contaminating stem cells present in purified MMCs. Firstly, pluripotent stem cell genes are not known to be present in the bone marrow, at least at detectable levels, and mesenchymal stem cells or hematopoietic stem cells are rare in the bone marrow (<1%) making difficult such a contamination. Secondly, several hundred of genes are specifically overexpressed in pluripotent stem cells, in mesenchymal stem cell or in hematopoietic progenitors compared to the other populations used in this study (results not shown), and in case of a contamination of purified MMCs by these stem cells, these genes should be also detected, which is not the case. Thirdly, the stem cell genes are also expressed in human myeloma cell lines that have been cultured for years in vitro and could not be contaminated by normal stem cells (supplementary Figure S1 and supplementary Figure S2).

The high enrichment of prognostic myeloma genes in normal stem cell genes may suggest the frequency of myeloma stem cells is increased in patients with a high ^SC^score and that these myeloma stem cells share common pathways with normal stem cells. Vice versa, the fact that these stem cell genes are overexpressed in malignant plasma cells in association with a poor survival could highlight an important role of the gene product in normal stem cell biology. The status of a myeloma stem cell is currently controversial. While some studies emphasized the myeloma stem cell could be a CD138^−^ plasma cells [15], others failed to confirm these data [16], [17]. A major difficulty to identify a myeloma stem cell, able to generate myeloma tumor, is the lack of animal models to efficiently engraft patients’ primary MMCs. The current study identifying prognostic genes shared by normal stem cells and MMCs from patients with poor survival may prompt investigating whether these “stem cell” myeloma genes encode for new markers of a putative MM stem cell.

This “stem cell” myeloma gene list comprises 37 genes, whose 18 are overexpressed in pluripotent stem cells, 10 in hematopoietic stem cells, and 9 in mesenchymal stem cells. Three of the 4 Yamanaka genes – *SOX2, KLF4 and MYC* - making it possible to reprogram adult cells into pluripotent stem cells have been shown to be expressed by MMCs [12]. These genes were not picked here because also they were expressed in differentiated cells (KLF4) or had no prognostic value for MM patient survival. Among the 37 stem cell genes, 8 encode for metabolic proteins (*PLOD2, GAMT, TM7SF2, FBXL7, GOLM1, KCTD3, SLC27A5* and *NUDT11*), 6 for membrane proteins (*IGF1R, BAMBI, BCHE, PKP2, ROBO1* and *CNIH4*), and 3 for transcription factors (*POLR2F, PBX1* and *NFIB*) as assayed using Gene Ontology (Supplementary Table S4). The prognostic value of *IGF-1R* gene expression has been already documented in MM [18] and the known biology of the most speaking genes is reviewed below. ***BAMBI*** (Bone morphogenetic protein and Activin Membrane-Bound Inhibitor) is a bad prognosis gene that encodes for a decoy receptor antagonizing the function of bone morphogenic protein (BMP) or Activin receptors. *BAMBI* is highly expressed in pluripotent stem cells (supplementary Figure S2). Several studies have shown that BMPs can yield to MMC cell cycle blockade and apoptosis [19], [20], in particular though SMAD signaling activation and blockade of MYC pathway [19], [20]. Thus a high expression of BAMBI on MMCs could antagonize an inhibitory effect of BMPs on MMC growth in vivo. ***ROBO1*** gene is a poor prognostic gene in MM. It is the only gene shared between with the current 37 “stem cell” myeloma genes and UAMS-HRS 70 genes. *ROBO1* is highly expressed by normal pluripotent stem cells and weakly by normal plasma cells (supplementary Figure S2). *ROBO1* encodes for the Roundabout 1 membrane receptor, which belongs to immunoglobulin superfamily and whose ligands are the SLIT proteins [21]. SLIT proteins bind to heparan sulfate chain proteoglycans, which favor their interaction with ROBO1. The interaction between ROBO1 and SLIT proteins plays critical role in mediating axon guidance during neural development [22], recruitment of endothelial cells during angiogenesis [23], and cancer cell migration [24]. Of note, *SLIT* genes are highly expressed by bone marrow stromal cells of patients with MM, unlike monocytes or osteoclasts (data not shown). The interaction of SLIT proteins and ROBO1 on MMCs should be greatly facilitated by the high expression of heparan sulfate chain syndecan-1 on MMCs [25] and favor MMC dissemination. ***BCHE*** gene is a bad prognostic gene in MM and encodes for butyrylcholinesterase, a non-specific cholinesterase enzyme that hydrolyses many different choline esters. Non-neuronal acetycholine is synthesized by the majority of cells [26], [27] and serves to regulate basic cellular functions, e.g. proliferation or differentiation. *BCHE* expression is high in pluripotent stem cells (supplementary Figure S2) and decreased upon their differentiation [28]. An inhibition of butyrylcholinesterase yields to inhibition of embryonic stem cell proliferation [29]. The involvement of cholinesterases in normal and malignant plasma cells is not known. *BCHE* gene is not expressed in normal plasmablasts or plasma cells and dichotomically expressed in MMCs (either highly expressed or not expressed) suggesting a strong regulation of *BCHE* gene expression (supplementary Figure S2). Butyrylcholinesterase inhibitors are being developed for neurodegenerative diseases [30] and their biological effects on MMC survival and proliferation should be investigated. ***GOLM1*** is a poor prognostic gene, overexpressed in pluripotent stem cells (supplementary Figure S2). It encodes for Golgi Protein 73 (GP73), whose serum level is a powerful diagnosis factor for hepatocarcinoma, without elucidation of the biological function of GP73. Of note, both hepatocytes and plasma cells produce a lot of proteins requiring high Golgi activity and increased unfold protein response. GP73 could be a marker of MMC dedifferentiation since it is transiently expressed in plasmablasts, lacking on memory B cells and mature plasma cells (supplementary Figure S2). This raises interest to investigate the prognostic value of serum GP73 levels in patients with MM and to further investigate the role of the golgi apparatus in normal and malignant plasma cells. ***PBX1*** gene is a bad prognostic factor in MM. PBX1 interacts with MEIS to bind to DNA element [31]. *PBX1* gene is fused to the transcription factor E2A as a result of the t(1;19) translocation in pre-B cell leukemia [32]. This fusion prevents interaction with MEIS proteins and converts PBX1 to a transcriptional activator. The *PBX1* gene is overexpressed in pluripotent stem cells (supplementary Figure S2) and downregulated during their differentiation [33]. Actually, PBX1 cooperate with KLF4 to induce expression of *NANOG* gene and protein, which is critical to maintain stem cell pluripotency [33]. A role of PBX1 in MM has not been documented. ***NANOS1*** is a bad prognostic gene overexpressed in pluripotent stem cells (supplementary Figure S2). NANOS1 protein bears a COOH-terminal (CCHC)(2) zinc finger domain and belongs to an evolutionarily conserved protein family sharing functions in germ cell development in both vertebrates and invertebrates [34]. NANOS1 is essential for both establishing and maintaining germline stem cells by preventing their precocious entry into oogenesis [35]. Thus *NANOS1* expression in MM could be associated in MMC dedifferentiation. ***Plakophilin 2*** (*PKP2*) is a bad prognostic gene in MM. It is not expressed in normal plasmablasts or plasma cells and is overexpressed in pluripotent stem cells (supplementary Figure S2). PKP2 is involved in desmosome junction in association with members of cadherin family, desmoplakin, desmoglein and desmocollin. *PKP2* could be associated with N-cadherin in adherens junctions in cancer cells and involved in immortalization of mesenchymal stem cells [36]. N-cadherin is expressed by MMCs and directly mediates the bone marrow localization and retention of MMCs in vivo, and facilitates a close interaction between N-cadherin positive MMCs and osteoblasts [37]. Given its association with N-cadherin in cancer cells, plakophilin could be one of the mediator favoring this N-cadherin mediated interaction of MMCs with the tumor niche. ***NUDT11*** is a gene coding for a type 3 diphosphoinositol polyphosphate phosphohydrolase. It is overexpressed in pluripotent stem cells (supplementary Figure S2) and has a very powerful poor prognostic value when overexpressed in MMCs of 12% of the patients. The role of NUDT11 for conferring a stem cell property and increased risk in cancer should be investigated. ***POLR2F*** gene is a bad prognostic factor in MM and is overexpressed in normal pluripotent stem cells (supplementary Figure S2). It encodes for a subunit of RNA polymerase II complex that is critical to insure transcription of genes into RNA. Its high expression in MMCs of patients with poor survival could be the reflection of a high transcription activity in MMCs of these patients with risk. ***MFAP3L*** is a good prognosis gene in MM, which is overexpressed in mesenchymal stem cells. It encodes for a microfibrillar-associated protein 3-like, whose function is poorly known. It is highly expressed in plasma cells from patients with MGUS and 40% of patients with MM and decreased in HMCLs (supplementary Figure S2). *MFAP3L* is expressed in memory B cells and normal bone marrow plasma cells, unlike plasmablasts (supplementary Figure S2). Whether *MFAP3L* could be involved in differentiation of mature plasma cells and stop in the cell cycle or is a surrogate marker of mature plasma cells needs further investigation.

This brief review outlines that this study has identified novel “stem cell’ myeloma genes, which could encode for markers of putative myeloma stem cells or for proteins involved in MMC dedifferentiation, interaction with the tumor environment, or tumor spreading. The identification of primary MMCs, HMCLs and normal stem cells expressing these genes will greatly facilitate the study of the biological function of these stem cell myeloma gene products.

## Materials and Methods

### Patients’ and Healthy Donors’ Primary Cells, Stem Cell Lines and Myeloma Cell Lines

In accordance with the Declaration of Helsinki and institutional research board approval from Montpellier or Heidelberg University hospitals, bone marrow Multiple Myeloma Cells (MMCs) from 206 patients with previously-untreated Multiple Myeloma were purified using anti-CD138 MACS microbeads (Miltenyi Biotec) achieving a purity ≥95%. Briefly, whole BM cells were collected after red blood cell lysis with NH_4_Cl, mononuclear cells recovered by Ficoll-density gradient centrifugation, MMCs labelled with anti-CD138 MACS microbeads (Miltenyi Biotec) and sorted with an automacs device. Bone marrow hematopoietic stem cells, T lymphocytes, monocytes or polymorphonuclear neutrophils were purified (purity ≥95%) from 5 newly-diagnosed patients by labelling bone marrow cells with an anti-CD34 mAb, anti-CD3 mAb, anti-CD14 mAb, or an anti-CD15 mAbs (all from BD Biosciences) and CD34+, CD3+, CD14+, and CD15+ cells were sorted with a FACSAria cell sorter (BD Biosciences) [38]. Osteoclasts were generated by in vitro differentiation of monocytes as previously described and contained ≥ 95% integrin αvβ3 positive cells [39]. Normal bone marrow plasma cells were purified from healthy donors (HDs, n = 5) using anti-CD138 microbeads. Bone marrow mesenchymal stem cells were obtained in our laboratory from 5 newly-diagnosed patients [40]. Preplasmablasts, plasmablasts or plasma cells were obtained using our 3-step in vitro model starting from purified memory B cells from 5 HDs [13], [14]. Human myeloma cell lines (HMCLs, n = 25) were obtained in our laboratory (XG series) [41], [42], [43] or were commercially available - SKMM, OPM2, LP1 and RPMI8226– (ATTC, LGC Standards, France). They were maintained in RPMI1640 (Gibco Invitrogen, France), 10% fetal bovine serum (FBS, PAA laboratory GmbH, Austria) and for the IL-6-dependant cell lines, with 2 ng/ml of IL-6 (Abcys SA, Paris, France). Their extensive phenotypic and molecular characteristics have been reported [44].

### Gene Expression Data

Gene expression profiling (GEP) of MMCs were from two independent large patients’ cohorts: the Heidelberg-Montpellier (HM) cohort and the University of Arkansas for Medical Sciences (UAMS, Little Rock, USA) cohort treated with total therapy 2) [45]. GEP were obtained using Affymetrix U133 2.0 plus array (Affymetrix, Santa Clara, CA). The clinical characteristics of our patient HM cohort are provided in supplementary Table S1 and the .CEL files and MAS5 files deposited at the ArrayExpress public database under accession number E-MTAB-362. The structural chromosomal aberrations including t(4;14)(p16.3;q32.3) and t(11;14)(q13;q32.3), as well as numerical aberrations including 17p13 and 1q21 gain, were assayed by fluorescence in situ hybridization (iFISH) [46]. Patients with t(4;14)(p16.3;q32.3) were also detected using spike *MMSET* expression in MMCs as a surrogate marker [18]. The GEP of MMCs of the 345 patients of the UAMS TT2 cohort are publicly available at (GEO, http://www.ncbi.nlm.nih.gov/geo/, accession number GSE2658) and were used as a validation set. As iFISH data were not available for UAMS-TT2 patients, t(4;14) translocation was evaluated using *MMSET* spike expression [18] and del17p13 surrogated by *TP53* probe set signal [47]. GEP (.CEL files and MAS5 files) of HMCLs and of the various purified normal cells are deposited at ArrayExpress under accession numbers E-TABM-937, E-TABM-1088, E-MEXP-2360, E-MEXP-2360, E-MEXP-3034, E-TABM-937. Affymetrix U133 plus 2.0 gene expression profiling of human embryonic stem cells were available from GEO (http://www.ncbi.nlm.nih.gov/geo/, GSE6561, GSE7234, GSE7896).

### Statistical Analysis

Affymetrix gene expression data were normalized using MAS5 Affymetrix algorithm with a scaling factor of 500. Two-class and multi-class SAM supervised analysis (1000 permutations, FDR ≤ 5%, ratio ≥ 2) were used to compare data sets of each cell type (http://www-stat.stanford.edu/~tibs/SAM/). Gene Expression Profiles (GEPs) were also analyzed with our bioinformatics platform (RAGE, http://rage.montp.inserm.fr) [48] and with the Amazonia website (http://amazonia.montp.inserm.fr/) [49]. The statistical significance of differences in overall survival between groups of patients was calculated by the log-rank test. Multivariate analysis was performed using the Cox proportional hazards model. Survival curves were plotted using the Kaplan-Meier method. All these analyses have been done with R.2.10.1 (http://www.r-project.org/) and bioconductor version 2.5. [50], [51]. Gene annotation and networks were generated through the use of Ingenuity Pathways Analysis (Ingenuity® Systems, Redwood City, CA).

### Building a Risk Score Using Stem Cell Genes

A “stem cell” risk score (termed ^SC^score) was built to group the prognostic information of the 37 stem cell myeloma genes within one parameter. For the 37 stem cell myeloma genes, the odd ratios of the Cox analysis on the HM cohort were determined with R MaxStat package, and for each patient, these odd ratios were weighted by +1 if the patient’s gene expression is above the Maxstat cutoff, and −1 if below or equal this cutoff. The ^SC^Score of a given patient was the sum of these weighted odd ratios for the 37 prognostic genes. Thus the higher the ^SC^Score is, the worse the prognosis is. Patients from the same cohort were ranked according to increased ^SC^Scores and for a given value S, the difference in overall survival of patients with a ^SC^Score ≤ S or > S was computed, making it possible to define the ^SC^Score value with a maximum difference in survival using maximally selected rank test from R package MaxStat.

## Supporting Information

Figure S1 **Expression of the 37 genes shared by multiple myeloma cells and adult or pluripotent stem cells in human myeloma cell lines.** Data are the MAS5-normalized expression signal of each gene in myeloma cell lines.
(PDF)

Figure S2 **Gene expression signal of** ***BAMBI, ROBO1, BCHE, GOLM1, PBX1, NANOS1, PKP2, NUDT11, POL2RF*** **and** ***MFAP3L.*** Gene expression was assayed using Affymetrix microarray in pluripotent stem cells (n = 13), hematopoietic stem cells (n = 5), mesenchymal stem cells (n = 5), memory b cells (n = 5), plasmablasts (n = 5), bone marrow plasma cells (n = 5), multiple myeloma cells (n = 206) and human myeloma cell lines (n = 25). Data are the log2 MAS5-normalized expression signal of each gene in the different cell populations.
(PDF)

Table S1 **Clinical characteristics of patients of HM cohort.** Data are median values and ranges for age, serum monoclonal protein, serum-β2-microglobulin and the Salmon-Durie and International Staging System (ISS) stages. NA, not available.
(PDF)

Table S2 The 885 unique probe sets overexpressed in MMCs or in HMCLs compared to normal counterparts.
(PDF)

Table S3 The 50 genes with pronostic value in HM and UAMS-TT2 cohorts.
(PDF)

Table S4 The 37 stem cell myeloma genes.
(PDF)

Table S5 **Clinical characteristics of patients in the 2 groups defined by ^SC^score.** The 206 previously-untreated patients of the HM cohort were treated at the university hospitals of Heidelberg and Montpellier. Patients were separated in 2 groups: low-risk (^SC^score ≤ −2.7) and high-risk (^SC^score > −2.7) ^SC^score groups. Data are the percentages of patients within these 2 groups with the indicated clinical or biological parameters. When the percentages were different with a chisquare test (P ≤ .05), data are shown in bold and italic.
(PDF)
