# Supplementary material for: Identification of Pluripotent and Adult Stem Cell Genes Unrelated to Cell Cycle and Associated with Poor Prognosis in Multiple Myeloma
Source: PLoS One. 2012 Jul 31;7(7):e42161. doi: 10.1371/journal.pone.0042161 (PMC3409163; doi:10.1371/journal.pone.0042161)
Supplement: Figure S1 — Expression of the 37 genes shared by multiple myeloma cells and adult or pluripotent stem cells in human myeloma cell lines. Data are the MAS5-normalized expression signal of each gene in myeloma cell lines. (PDF) [file pone.0042161.s001.pdf]

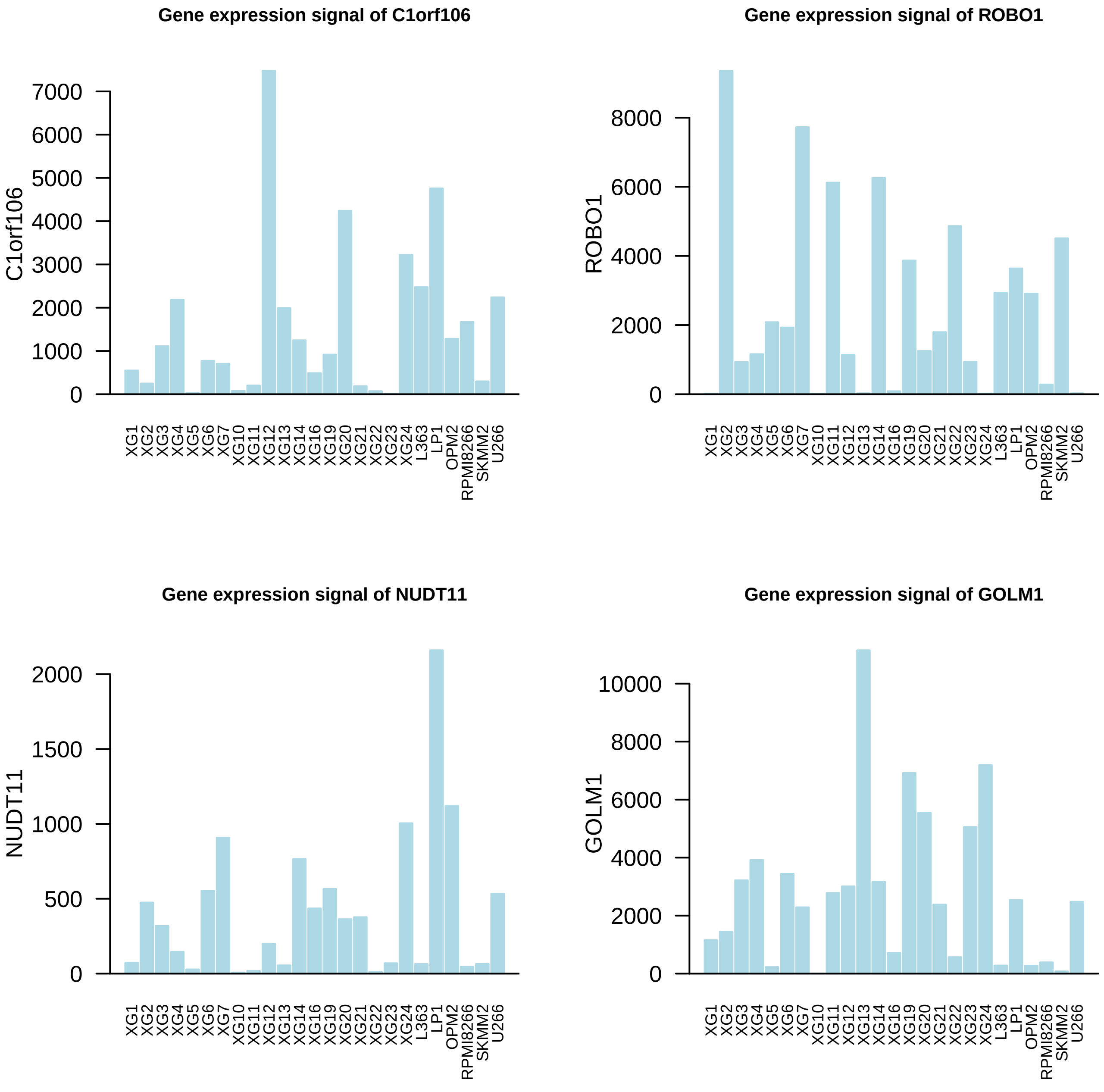
Gene expression signal of C1orf106
C1orf106
0
1000
2000
3000
4000
5000
6000
7000
Gene expression signal of ROBO1
ROBO1
0
2000
4000
6000
8000
Gene expression signal of NUDT11
NUDT11
0
500
1000
1500
2000
Gene expression signal of GOLM1
GOLM1
0
2000
4000
6000
8000
10000
XG1
XG2
XG3
XG4
XG5
XG6
XG7
XG10
XG11
XG12
XG13
XG14
XG16
XG19
XG20
XG21
XG22
XG23
XG24
L363
LP1
OPM2
RPMI8266
SKMM2
U266

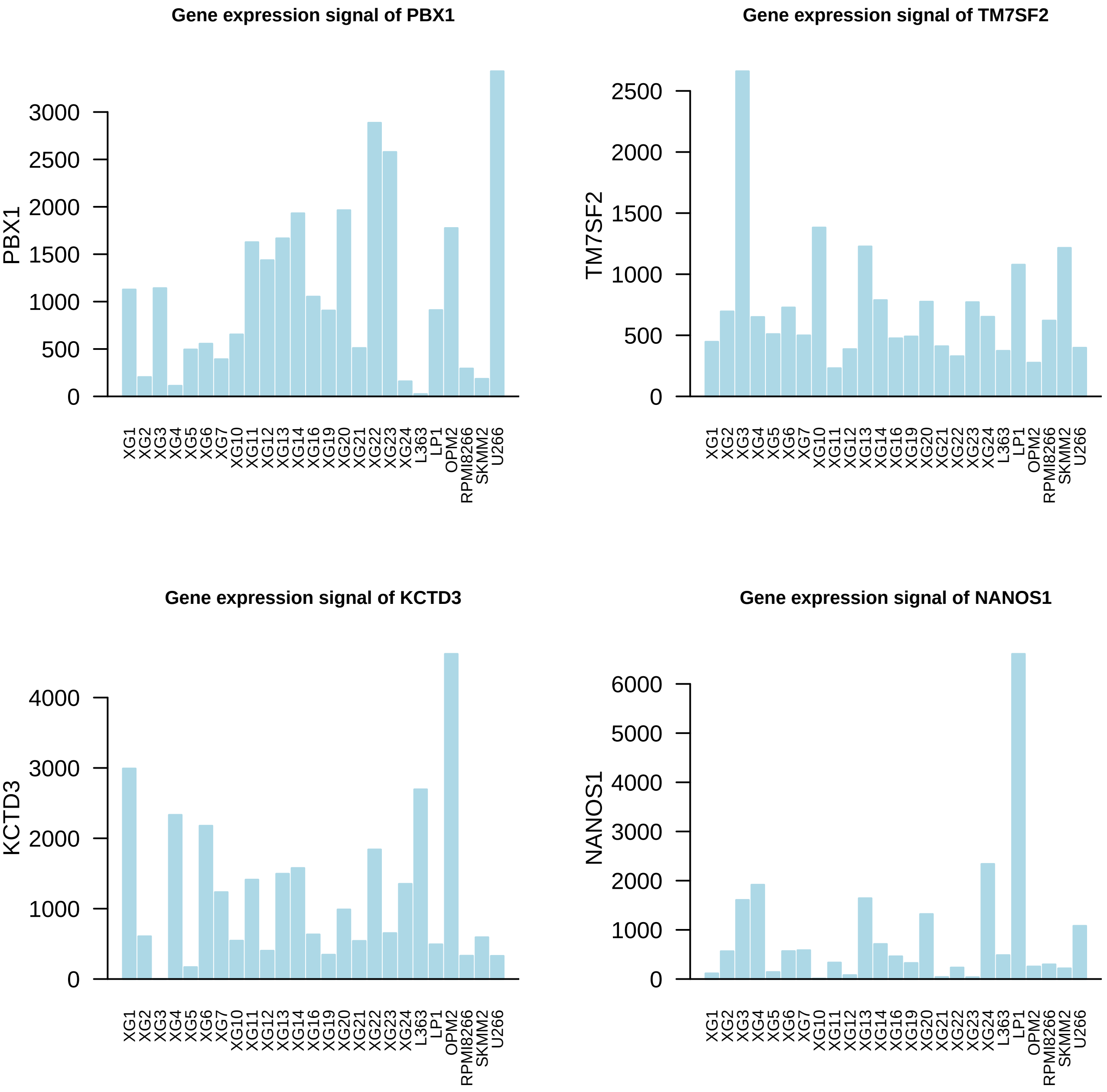

Gene expression signal of PBX1
PBX1
0
500
1000
1500
2000
2500
3000
XG1 XG2 XG3 XG4 XG5 XG6 XG7 XG10 XG11 XG12 XG13 XG14 XG16 XG19 XG20 XG21 XG22 XG23 XG24 L363 LP1 OPM2 RPMI8266 SKMM2 U266
Gene expression signal of TM7SF2
TM7SF2
0
500
1000
1500
2000
2500
XG1 XG2 XG3 XG4 XG5 XG6 XG7 XG10 XG11 XG12 XG13 XG14 XG16 XG19 XG20 XG21 XG22 XG23 XG24 L363 LP1 OPM2 RPMI8266 SKMM2 U266
Gene expression signal of KCTD3
KCTD3
0
1000
2000
3000
4000
XG1 XG2 XG3 XG4 XG5 XG6 XG7 XG10 XG11 XG12 XG13 XG14 XG16 XG19 XG20 XG21 XG22 XG23 XG24 L363 LP1 OPM2 RPMI8266 SKMM2 U266
Gene expression signal of NANOS1
NANOS1
0
1000
2000
3000
4000
5000
6000
XG1 XG2 XG3 XG4 XG5 XG6 XG7 XG10 XG11 XG12 XG13 XG14 XG16 XG19 XG20 XG21 XG22 XG23 XG24 L363 LP1 OPM2 RPMI8266 SKMM2 U266

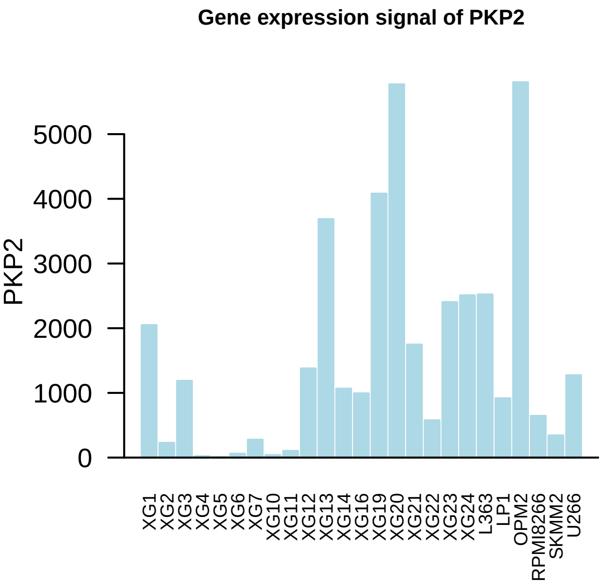
Gene expression signal of PKP2
PKP2
0
1000
2000
3000
4000
5000
XG1 XG2 XG3 XG4 XG5 XG6 XG7 XG10 XG11 XG12 XG13 XG14 XG16 XG19 XG20 XG21 XG22 XG23 XG24 L363 LP1 OPM2 RPMI8266 SKMM2 U266

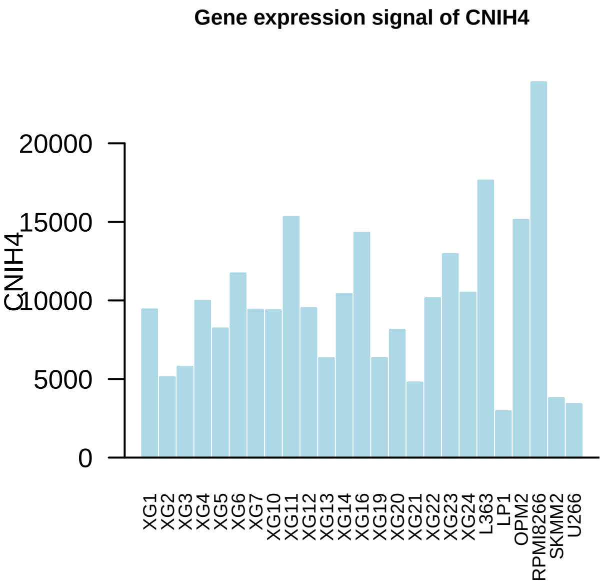
Gene expression signal of CNIH4
CNIH4
0
5000
10000
15000
20000
XG1 XG2 XG3 XG4 XG5 XG6 XG7 XG10 XG11 XG12 XG13 XG14 XG16 XG19 XG20 XG21 XG22 XG23 XG24 L363 LP1 OPM2 RPMI8266 SKMM2 U266

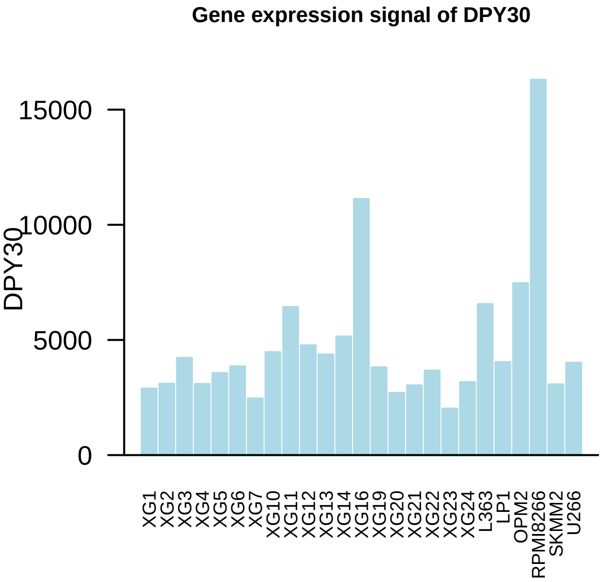
Gene expression signal of DPY30
DPY30
0
5000
10000
15000
XG1 XG2 XG3 XG4 XG5 XG6 XG7 XG10 XG11 XG12 XG13 XG14 XG16 XG19 XG20 XG21 XG22 XG23 XG24 L363 LP1 OPM2 RPMI8266 SKMM2 U266

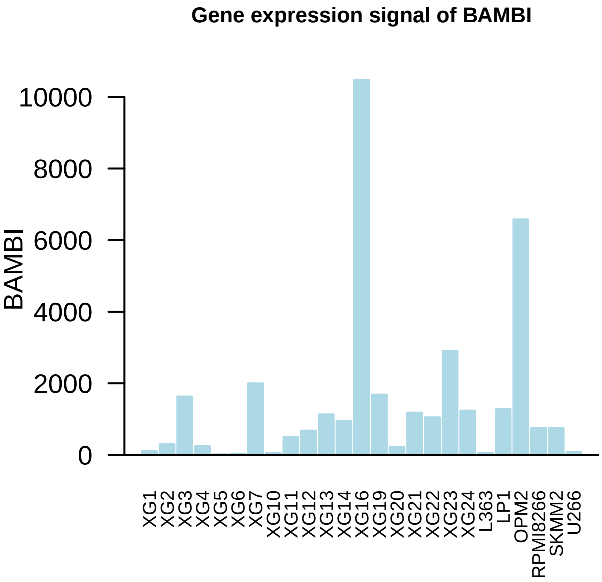
Gene expression signal of BAMBI
BAMBI
0
2000
4000
6000
8000
10000
XG1 XG2 XG3 XG4 XG5 XG6 XG7 XG10 XG11 XG12 XG13 XG14 XG16 XG19 XG20 XG21 XG22 XG23 XG24 L363 LP1 OPM2 RPMI8266 SKMM2 U266

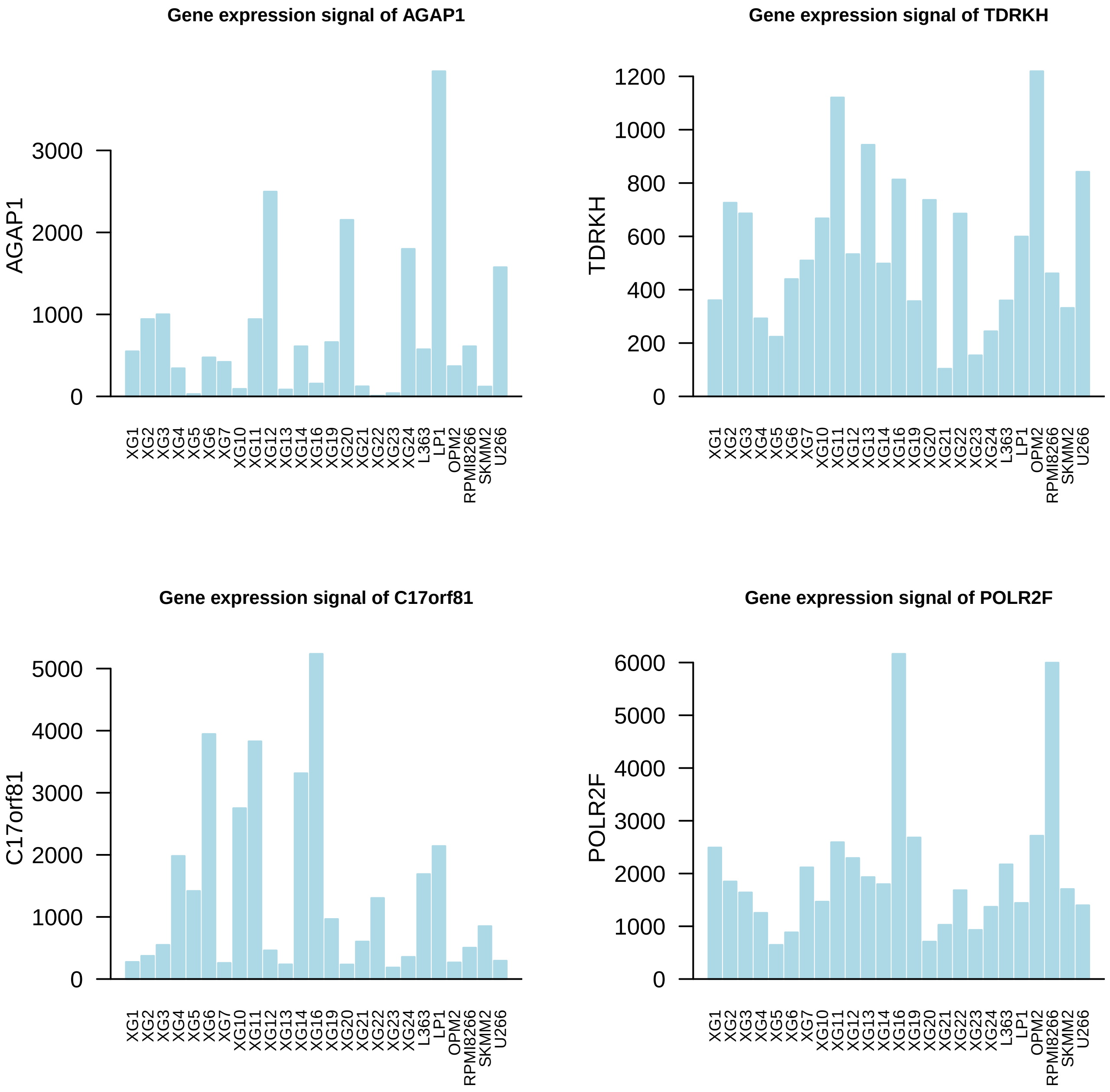
Gene expression signal of AGAP1
AGAP1
0
1000
2000
3000
XG1 XG2 XG3 XG4 XG5 XG6 XG7 XG10 XG11 XG12 XG13 XG14 XG16 XG19 XG20 XG21 XG22 XG23 XG24 L363 LP1 OPM2 RPMI8266 SKMM2 U266
Gene expression signal of TDRKH
TDRKH
0
200
400
600
800
1000
1200
XG1 XG2 XG3 XG4 XG5 XG6 XG7 XG10 XG11 XG12 XG13 XG14 XG16 XG19 XG20 XG21 XG22 XG23 XG24 L363 LP1 OPM2 RPMI8266 SKMM2 U266
Gene expression signal of C17orf81
C17orf81
0
1000
2000
3000
4000
5000
XG1 XG2 XG3 XG4 XG5 XG6 XG7 XG10 XG11 XG12 XG13 XG14 XG16 XG19 XG20 XG21 XG22 XG23 XG24 L363 LP1 OPM2 RPMI8266 SKMM2 U266
Gene expression signal of POLR2F
POLR2F
0
1000
2000
3000
4000
5000
6000
XG1 XG2 XG3 XG4 XG5 XG6 XG7 XG10 XG11 XG12 XG13 XG14 XG16 XG19 XG20 XG21 XG22 XG23 XG24 L363 LP1 OPM2 RPMI8266 SKMM2 U266

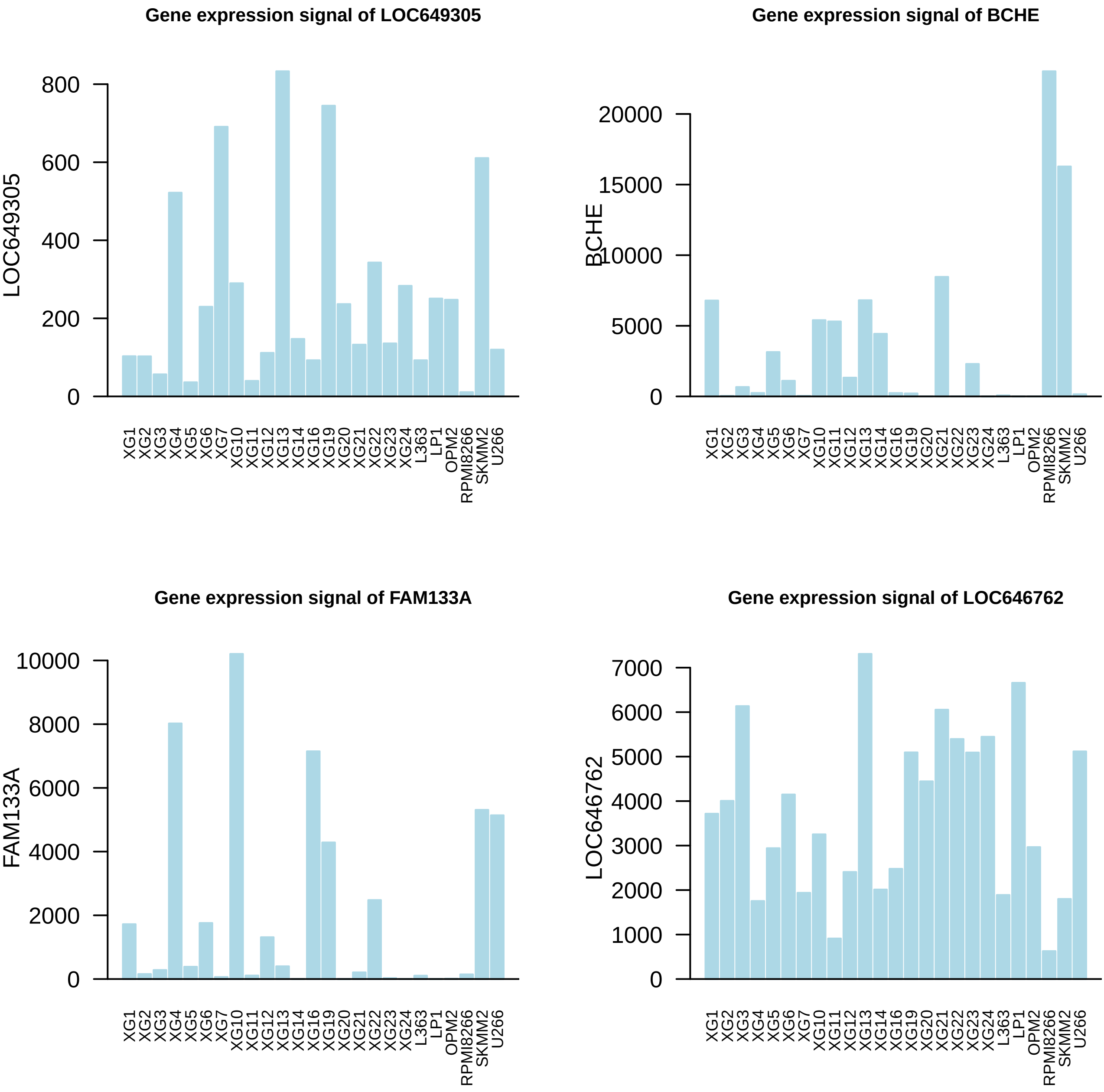
Gene expression signal of LOC649305
LOC649305
0
200
400
600
800
Gene expression signal of BCHE
BCHE
0
5000
10000
15000
20000
Gene expression signal of FAM133A
FAM133A
0
2000
4000
6000
8000
10000
Gene expression signal of LOC646762
LOC646762
0
1000
2000
3000
4000
5000
6000
7000
XG1
XG2
XG3
XG4
XG5
XG6
XG7
XG10
XG11
XG12
XG13
XG14
XG16
XG19
XG20
XG21
XG22
XG23
XG24
L363
LP1
OPM2
RPMI8266
SKMM2
U266

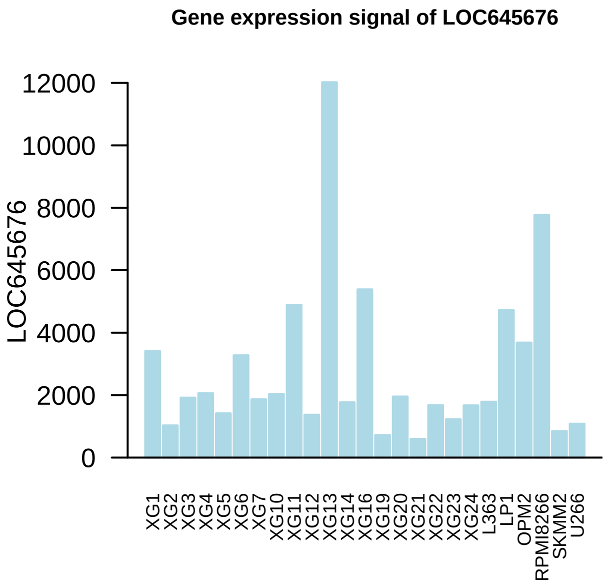
Gene expression signal of LOC645676
LOC645676
0
2000
4000
6000
8000
10000
12000
XG1
XG2
XG3
XG4
XG5
XG6
XG7
XG10
XG11
XG12
XG13
XG14
XG16
XG19
XG20
XG21
XG22
XG23
XG24
L363
LP1
OPM2
RPMI8266
SKMM2
U266

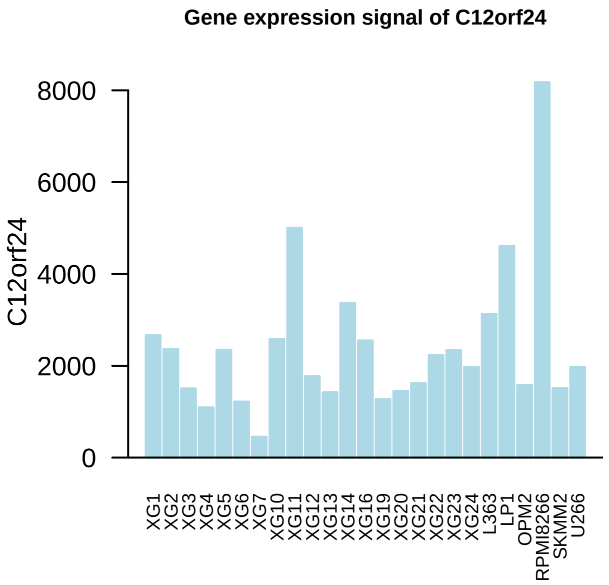
Gene expression signal of C12orf24
C12orf24
0
2000
4000
6000
8000
XG1
XG2
XG3
XG4
XG5
XG6
XG7
XG10
XG11
XG12
XG13
XG14
XG16
XG19
XG20
XG21
XG22
XG23
XG24
L363
LP1
OPM2
RPMI8266
SKMM2
U266

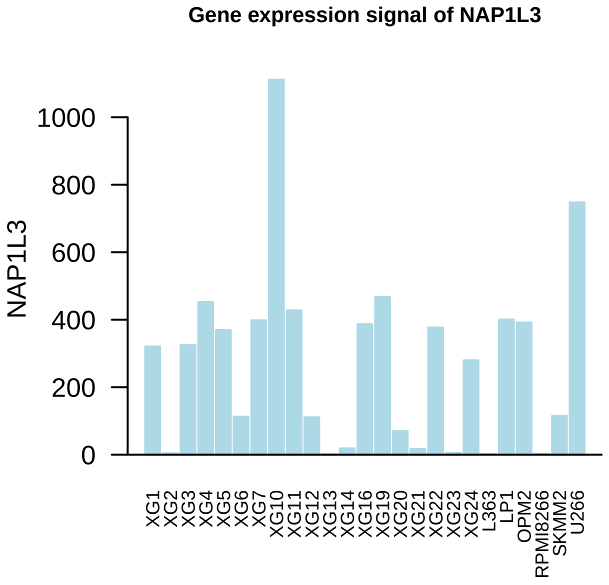
Gene expression signal of NAP1L3
NAP1L3
0
200
400
600
800
1000
XG1
XG2
XG3
XG4
XG5
XG6
XG7
XG10
XG11
XG12
XG13
XG14
XG16
XG19
XG20
XG21
XG22
XG23
XG24
L363
LP1
OPM2
RPMI8266
SKMM2
U266

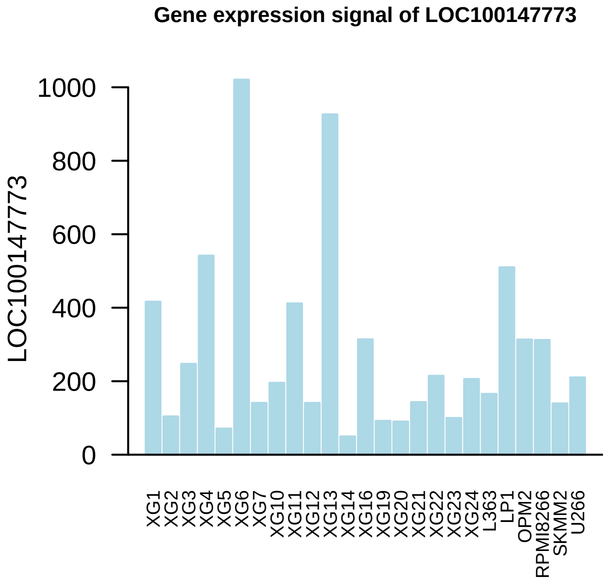
Gene expression signal of LOC100147773
LOC100147773
0
200
400
600
800
1000
XG1
XG2
XG3
XG4
XG5
XG6
XG7
XG10
XG11
XG12
XG13
XG14
XG16
XG19
XG20
XG21
XG22
XG23
XG24
L363
LP1
OPM2
RPMI8266
SKMM2
U266

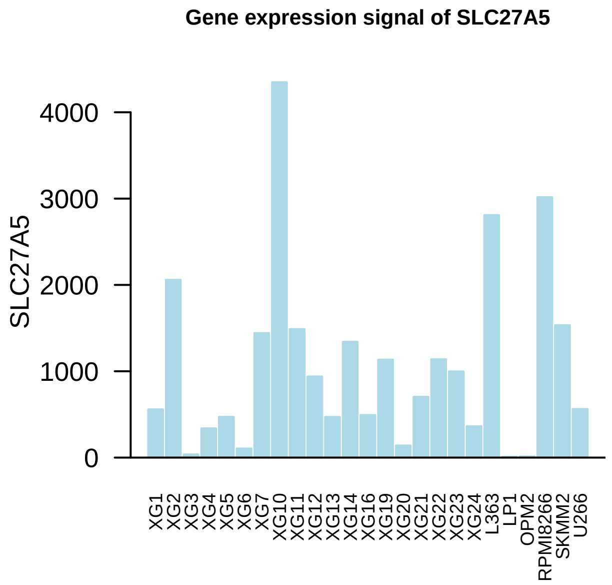
Gene expression signal of SLC27A5
SLC27A5
0
1000
2000
3000
4000
XG1 XG2 XG3 XG4 XG5 XG6 XG7 XG10 XG11 XG12 XG13 XG14 XG16 XG19 XG20 XG21 XG22 XG23 XG24 L363 LP1 OPM2 RPMI8266 SKMM2 U266

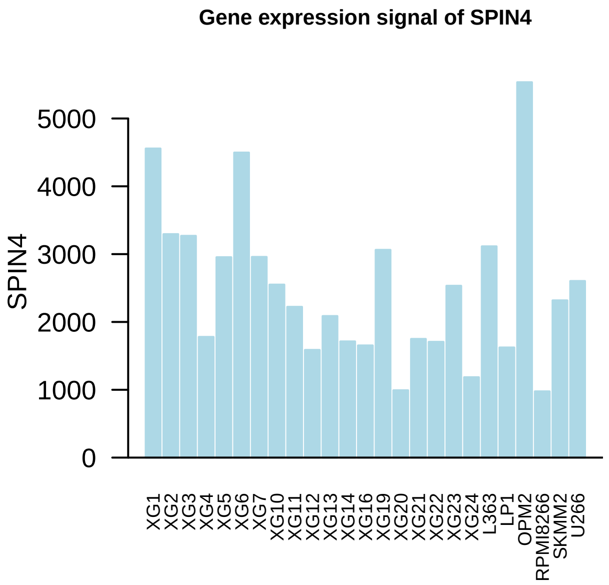
Gene expression signal of SPIN4
SPIN4
0
1000
2000
3000
4000
5000
XG1 XG2 XG3 XG4 XG5 XG6 XG7 XG10 XG11 XG12 XG13 XG14 XG16 XG19 XG20 XG21 XG22 XG23 XG24 L363 LP1 OPM2 RPMI8266 SKMM2 U266

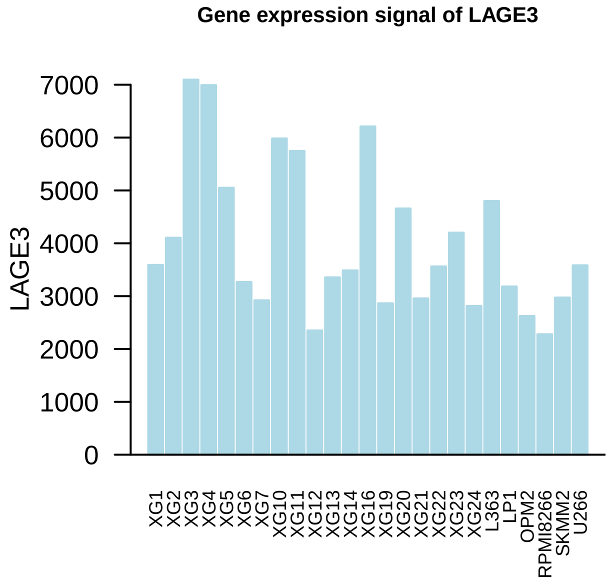
Gene expression signal of LAGE3
LAGE3
0
1000
2000
3000
4000
5000
6000
7000
XG1 XG2 XG3 XG4 XG5 XG6 XG7 XG10 XG11 XG12 XG13 XG14 XG16 XG19 XG20 XG21 XG22 XG23 XG24 L363 LP1 OPM2 RPMI8266 SKMM2 U266

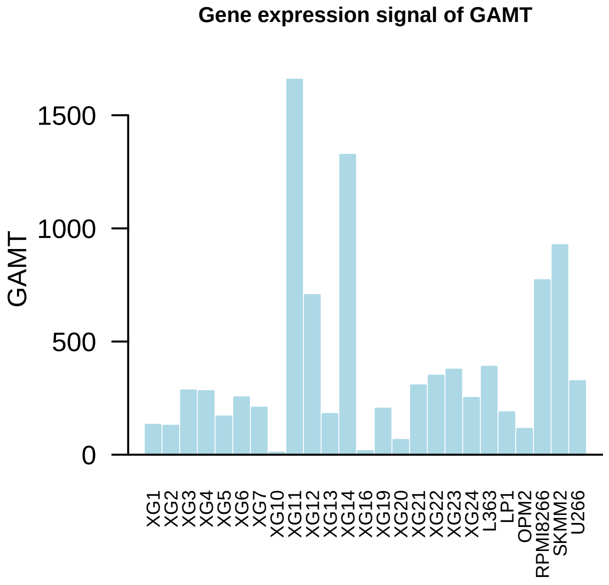
Gene expression signal of GAMT
GAMT
0
500
1000
1500
XG1 XG2 XG3 XG4 XG5 XG6 XG7 XG10 XG11 XG12 XG13 XG14 XG16 XG19 XG20 XG21 XG22 XG23 XG24 L363 LP1 OPM2 RPMI8266 SKMM2 U266

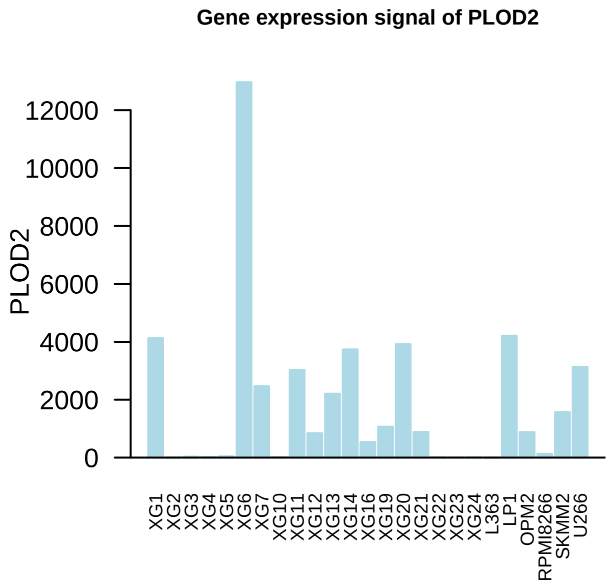
Gene expression signal of PLOD2
PLOD2
0
2000
4000
6000
8000
10000
12000
XG1
XG2
XG3
XG4
XG5
XG6
XG7
XG10
XG11
XG12
XG13
XG14
XG16
XG19
XG20
XG21
XG22
XG23
XG24
L363
LP1
OPM2
RPMI8266
SKMM2
U266

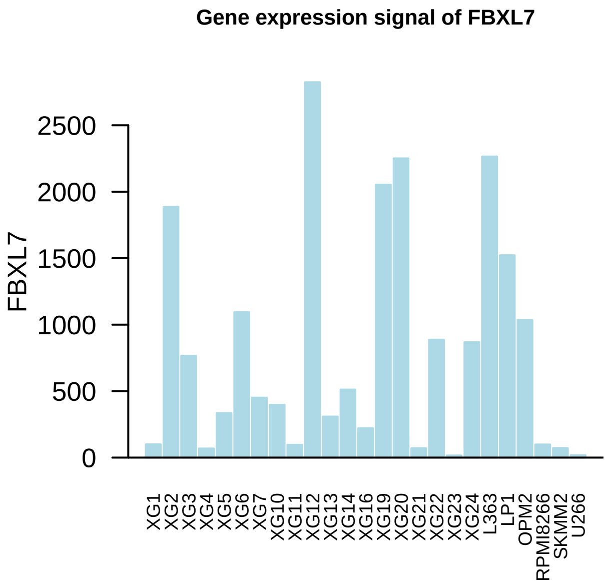
Gene expression signal of FBXL7
FBXL7
0
500
1000
1500
2000
2500
XG1
XG2
XG3
XG4
XG5
XG6
XG7
XG10
XG11
XG12
XG13
XG14
XG16
XG19
XG20
XG21
XG22
XG23
XG24
L363
LP1
OPM2
RPMI8266
SKMM2
U266

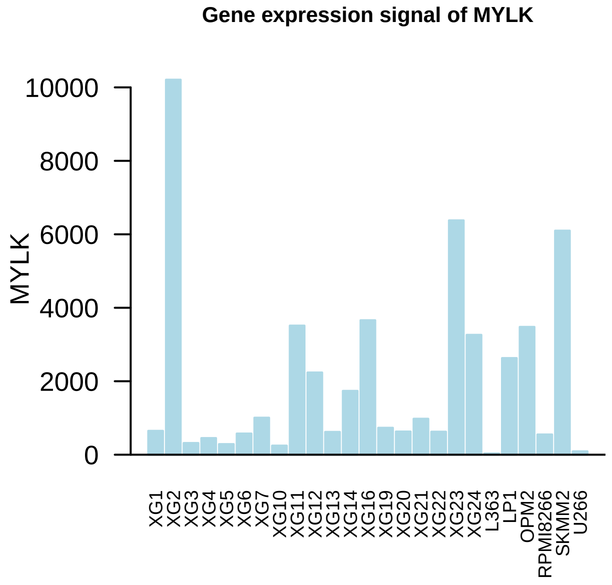
Gene expression signal of MYLK
MYLK
0
2000
4000
6000
8000
10000
XG1
XG2
XG3
XG4
XG5
XG6
XG7
XG10
XG11
XG12
XG13
XG14
XG16
XG19
XG20
XG21
XG22
XG23
XG24
L363
LP1
OPM2
RPMI8266
SKMM2
U266

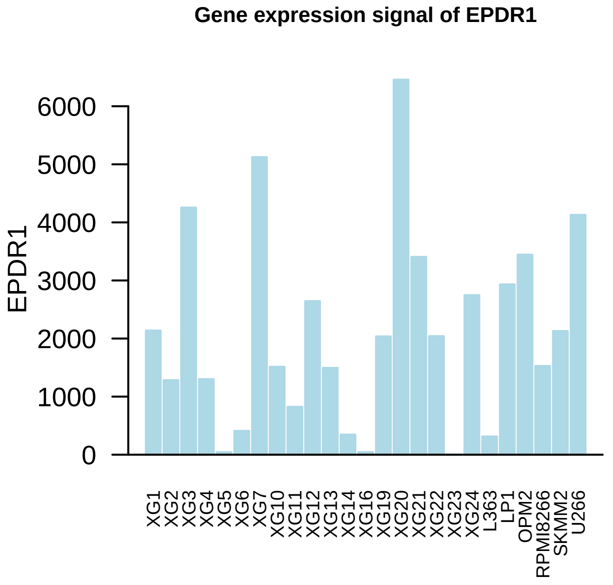
Gene expression signal of EPDR1
EPDR1
0
1000
2000
3000
4000
5000
6000
XG1
XG2
XG3
XG4
XG5
XG6
XG7
XG10
XG11
XG12
XG13
XG14
XG16
XG19
XG20
XG21
XG22
XG23
XG24
L363
LP1
OPM2
RPMI8266
SKMM2
U266

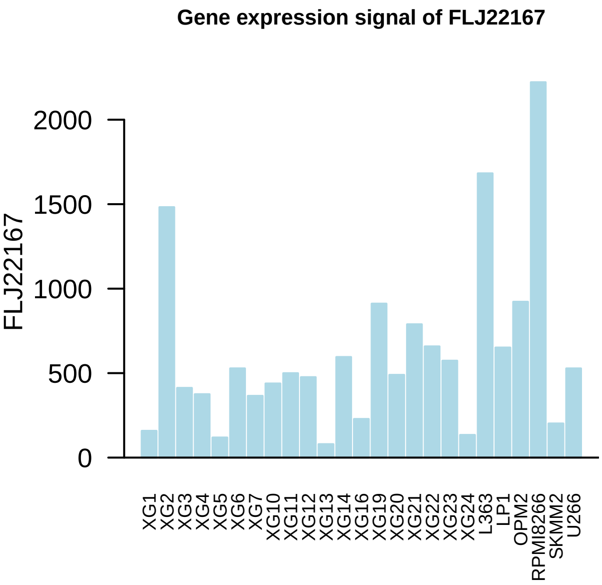
Gene expression signal of FLJ22167
FLJ22167
0
500
1000
1500
2000
XG1 XG2 XG3 XG4 XG5 XG6 XG7 XG10 XG11 XG12 XG13 XG14 XG16 XG19 XG20 XG21 XG22 XG23 XG24 L363 LP1 OPM2 RPMI8266 SKMM2 U266

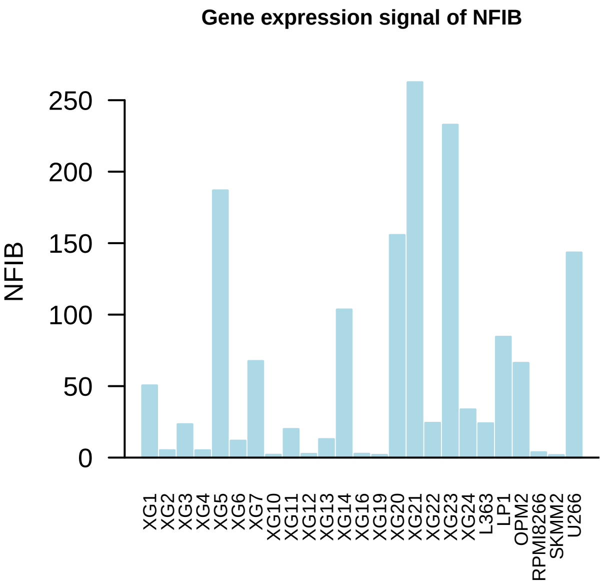
Gene expression signal of NFIB
NFIB
0
50
100
150
200
250
XG1 XG2 XG3 XG4 XG5 XG6 XG7 XG10 XG11 XG12 XG13 XG14 XG16 XG19 XG20 XG21 XG22 XG23 XG24 L363 LP1 OPM2 RPMI8266 SKMM2 U266

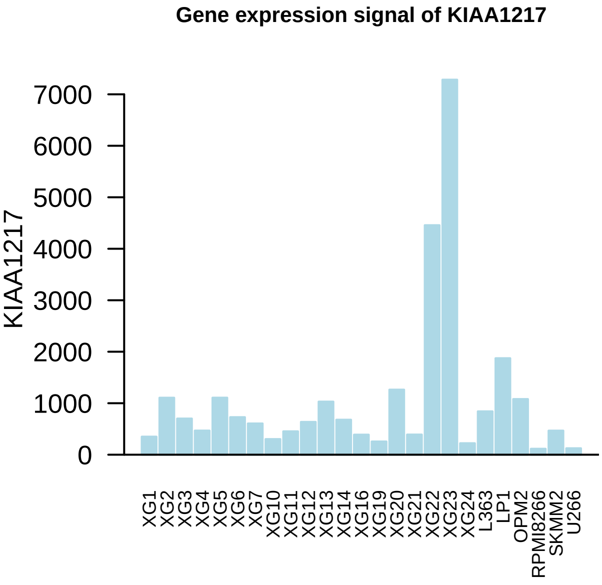
Gene expression signal of KIAA1217
KIAA1217
0
1000
2000
3000
4000
5000
6000
7000
XG1 XG2 XG3 XG4 XG5 XG6 XG7 XG10 XG11 XG12 XG13 XG14 XG16 XG19 XG20 XG21 XG22 XG23 XG24 L363 LP1 OPM2 RPMI8266 SKMM2 U266

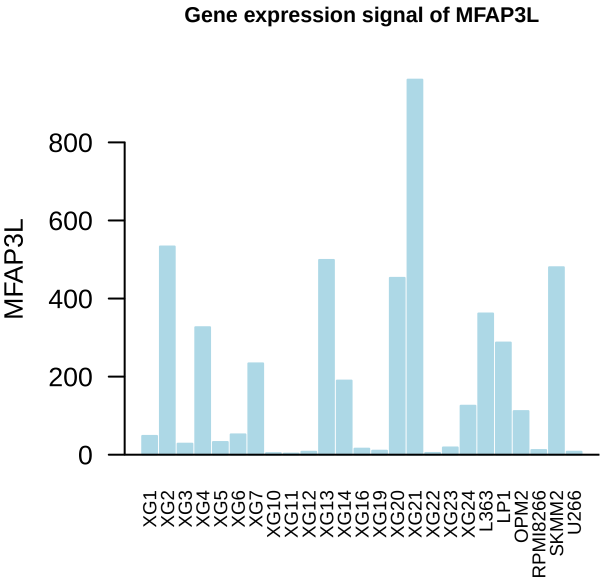
Gene expression signal of MFAP3L
MFAP3L
0
200
400
600
800
XG1 XG2 XG3 XG4 XG5 XG6 XG7 XG10 XG11 XG12 XG13 XG14 XG16 XG19 XG20 XG21 XG22 XG23 XG24 L363 LP1 OPM2 RPMI8266 SKMM2 U266

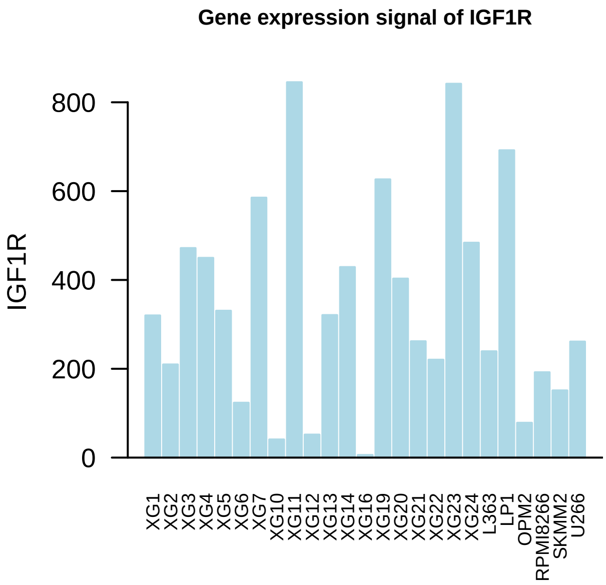
Gene expression signal of IGF1R
IGF1R
0
200
400
600
800
XG1
XG2
XG3
XG4
XG5
XG6
XG7
XG10
XG11
XG12
XG13
XG14
XG16
XG19
XG20
XG21
XG22
XG23
XG24
L363
LP1
OPM2
RPMI8266
SKMM2
U266
