# Supplementary material for: Identification of Pluripotent and Adult Stem Cell Genes Unrelated to Cell Cycle and Associated with Poor Prognosis in Multiple Myeloma
Source: PLoS One. 2012 Jul 31;7(7):e42161. doi: 10.1371/journal.pone.0042161 (PMC3409163; doi:10.1371/journal.pone.0042161)

Figure S2

BAMBI (203304_at); BMP and activin membrane-bound inhibitor homolog (Xenopus laevis); **BAD prognostic**; overexpressed in **pluripotent stem cells** (PSC)

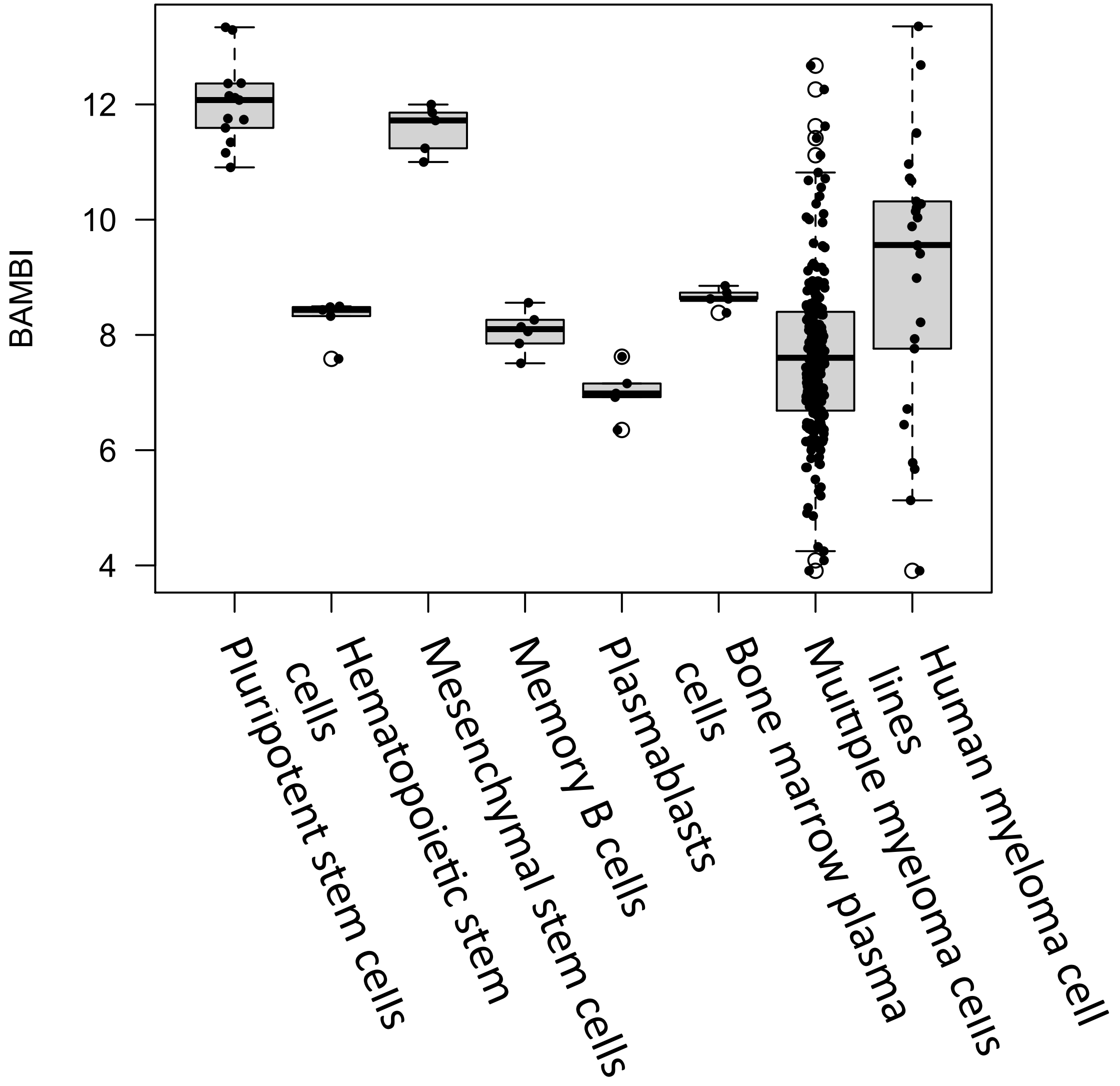

Figure S2

ROBO1 (213194_at); roundabout, axon guidance receptor, homolog 1 (Drosophila); **BAD prognostic**; overexpressed in **pluripotent stem cells** (PSC)

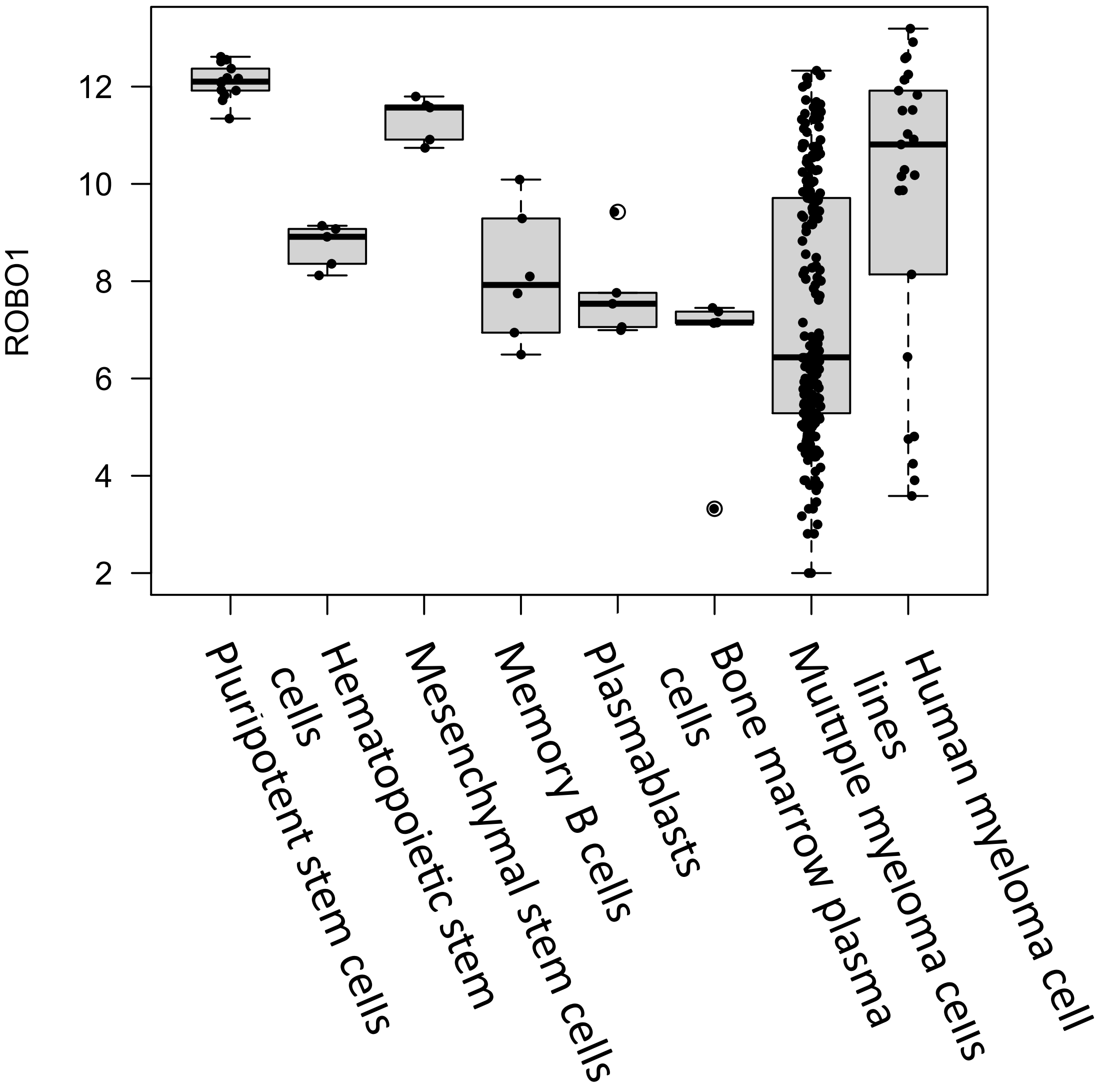

Figure S2

BCHE (205433_at); butyrylcholinesterase; **BAD prognostic**; overexpressed in **pluripotent stem cells** (PSC)

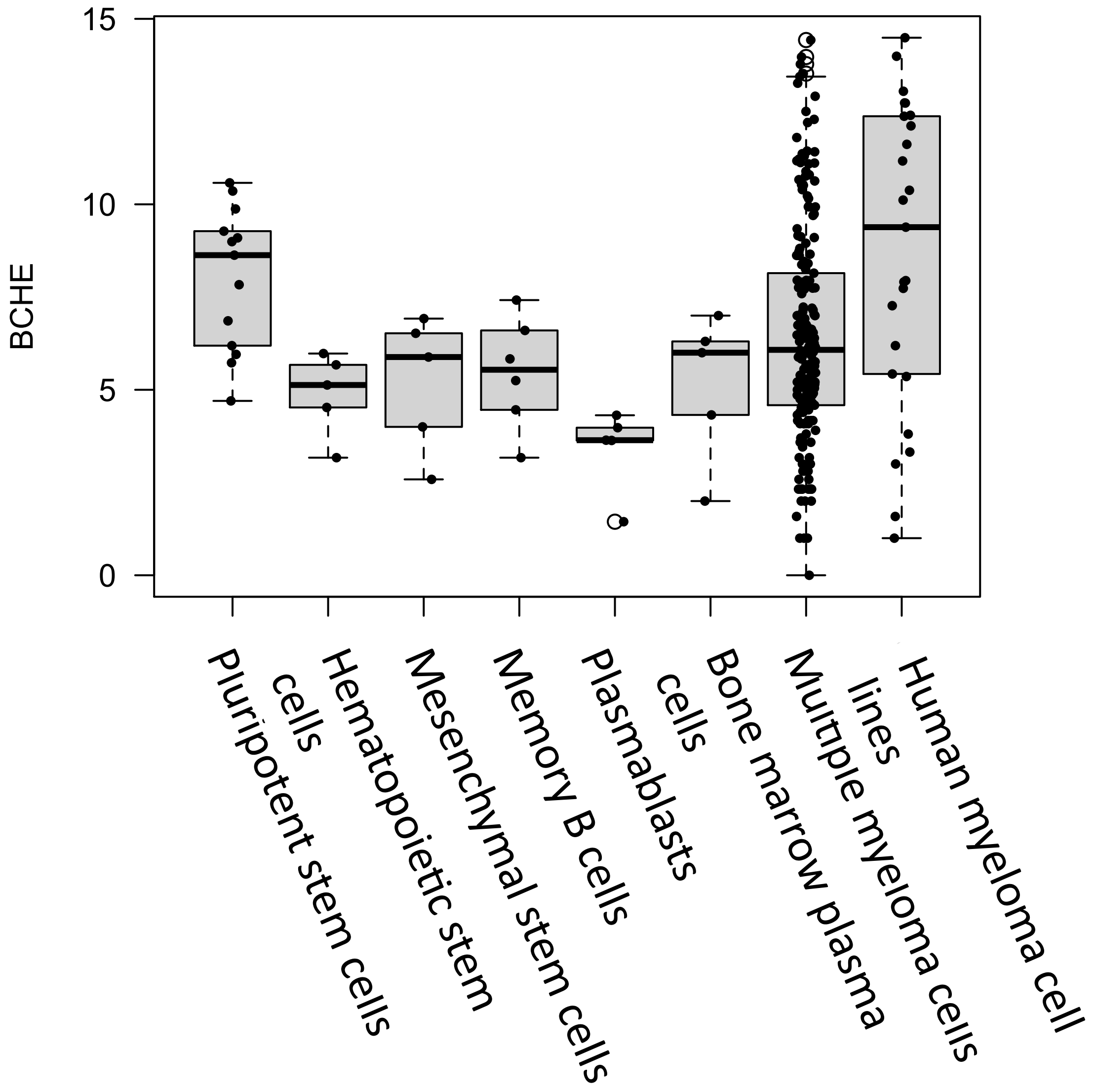

Figure S2

GOLM1 (217771_at); golgi membrane protein 1; **BAD prognostic**; overexpressed in **pluripotent stem cells** (PSC)

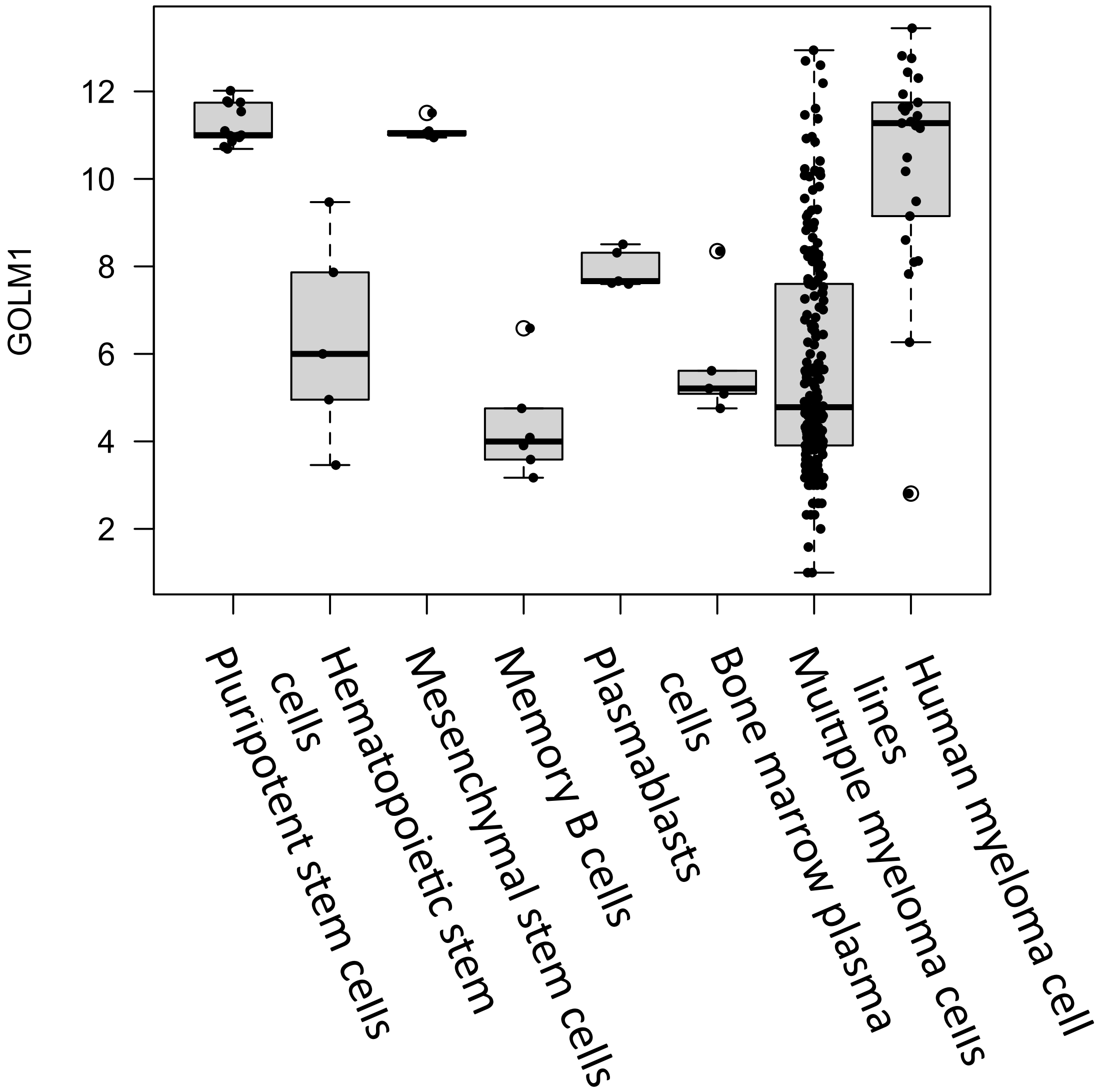

Figure S2

PBX1 (212151_at); pre-B-cell leukemia homeobox 1; **BAD prognostic**; overexpressed in **pluripotent stem cells** (PSC)

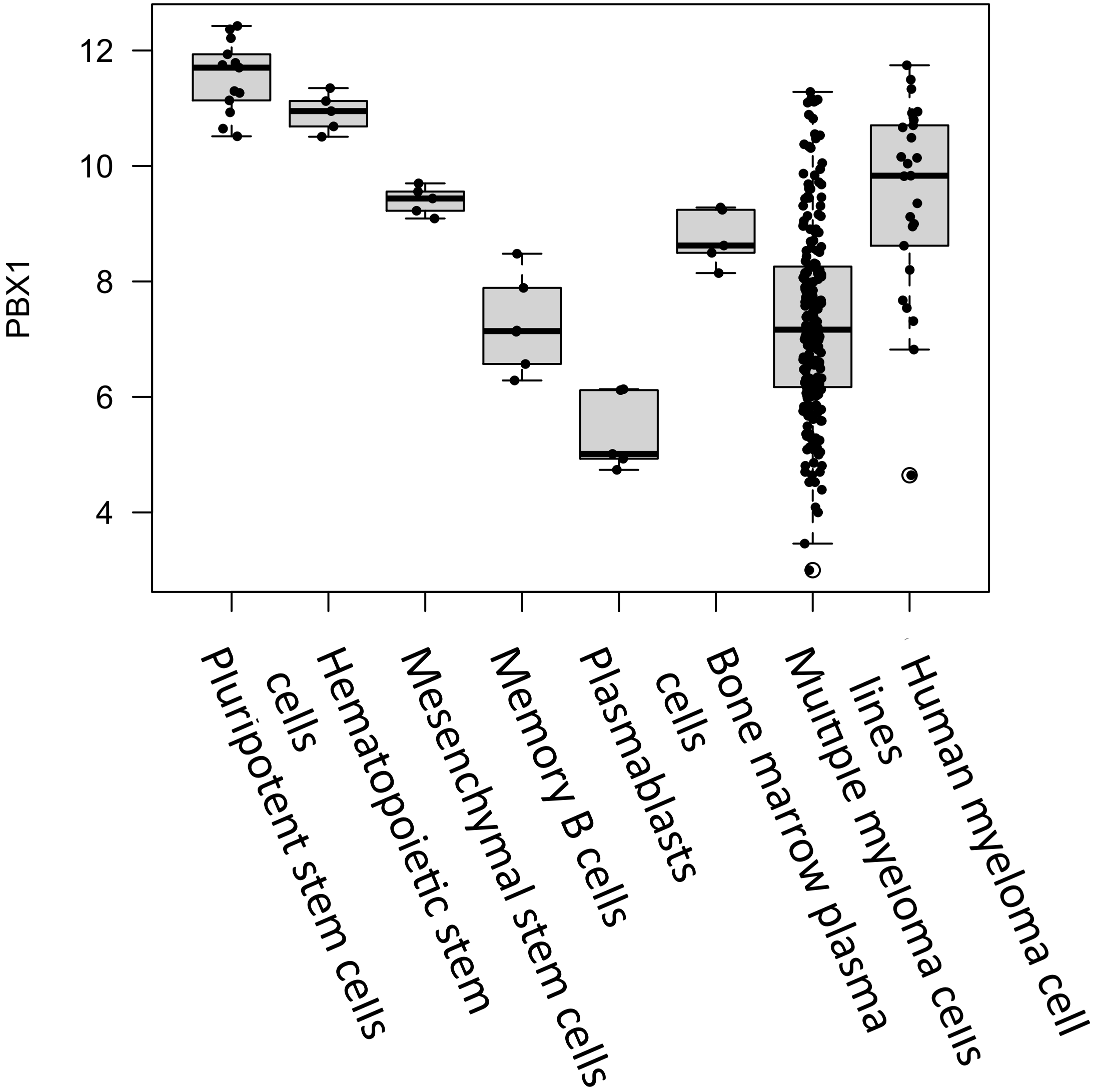

Figure S2

NANOS1 (228523_at); nanos homolog 1 (Drosophila); **BAD prognostic**; overexpressed in **pluripotent stem cells** (PSC)

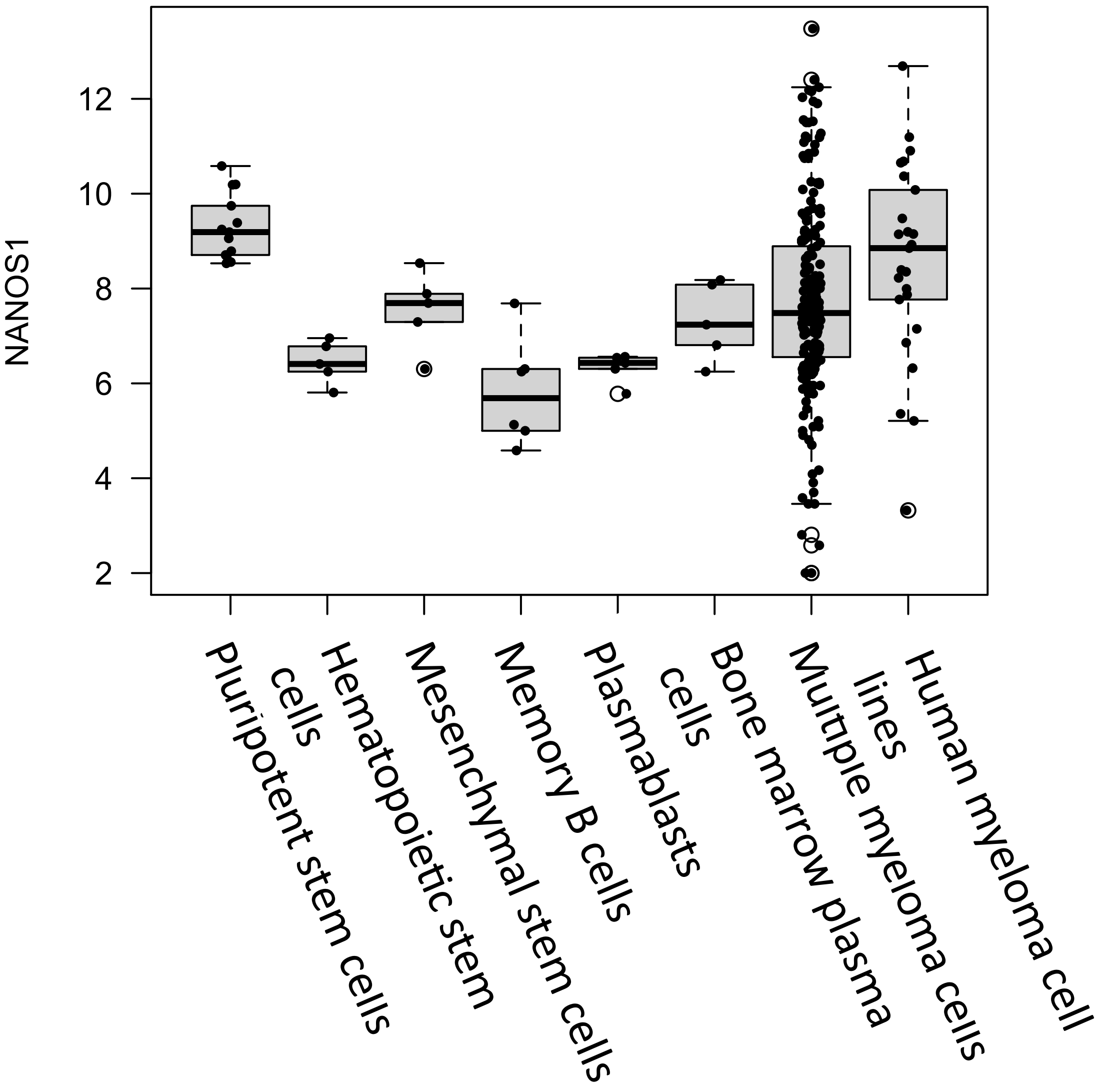

Figure S2

PKP2 (207717_s_at); plakophilin 2; **BAD prognostic**; overexpressed in **pluripotent stem cells** (PSC)

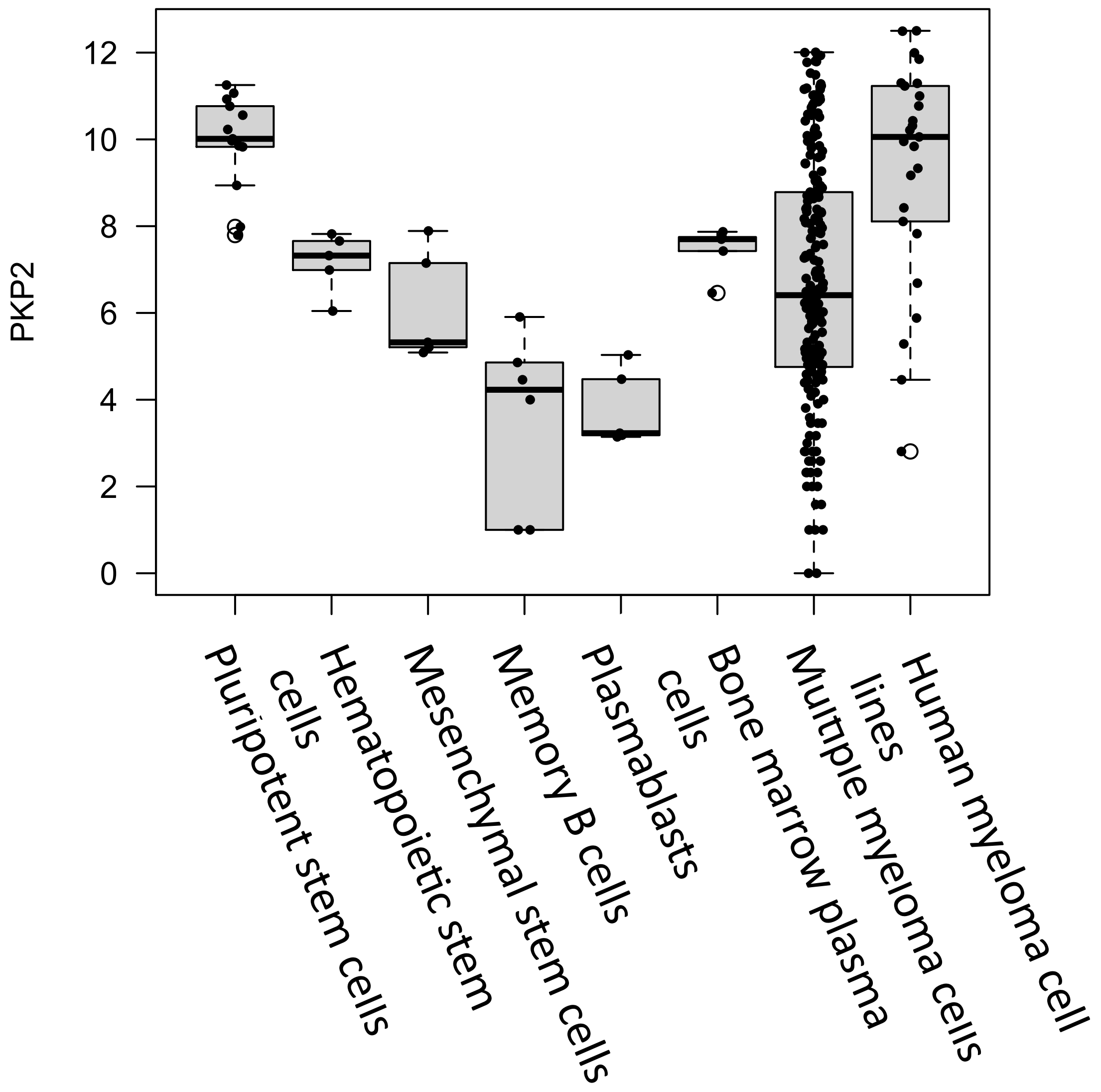

Figure S2

NUDT11 (219855_at); nudix (nucleoside diphosphate linked moiety X)-type motif 11; **bad prognostic**; overexpressed in **pluripotent stem cells** (PSC)

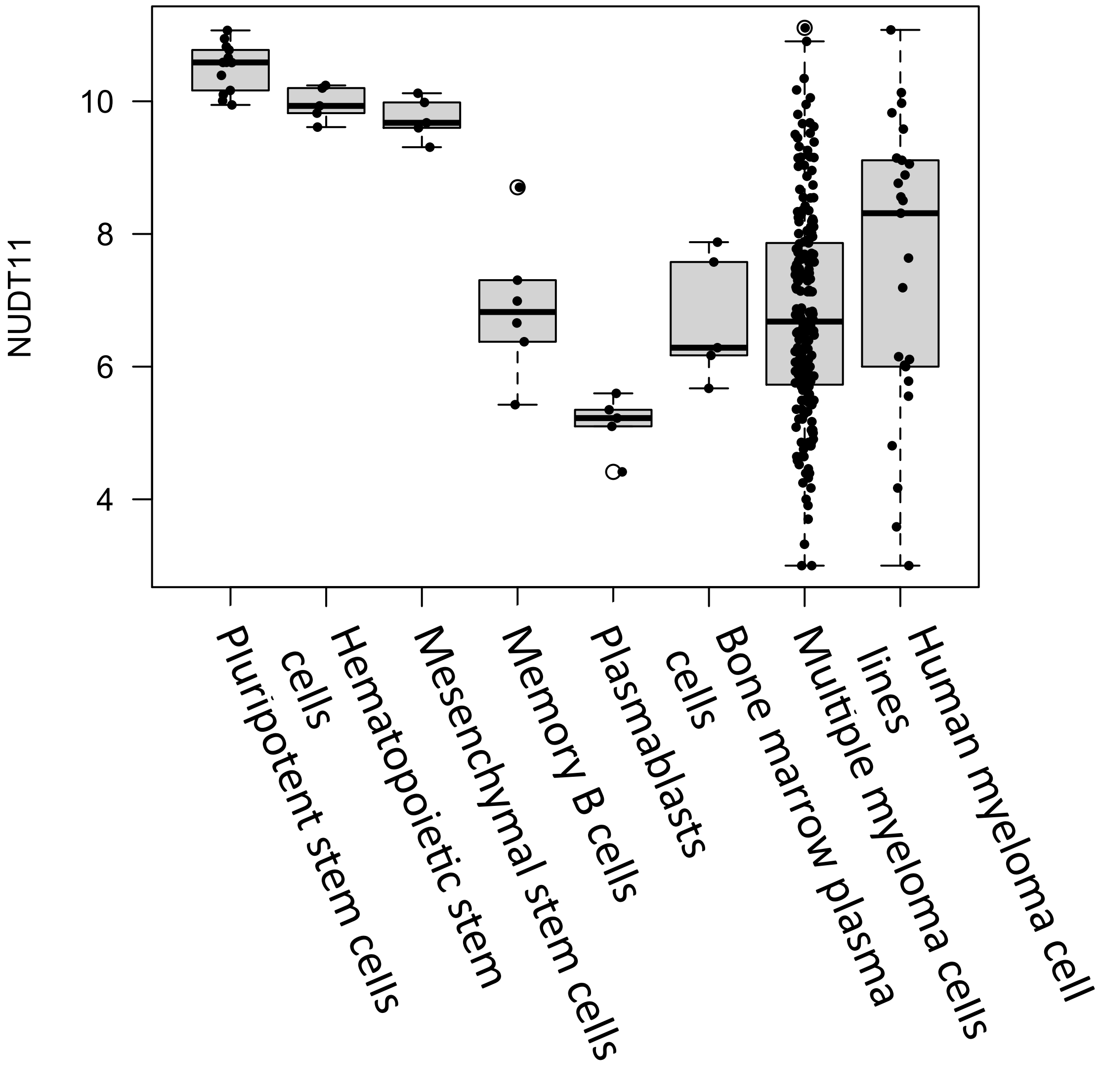

Figure S2

***POL2RF (**209511_at**)**; polymerase (RNA) II (DNA directed) polypeptide F; **bad prognostic**; overexpressed in **pluripotent stem cells** (PSC)

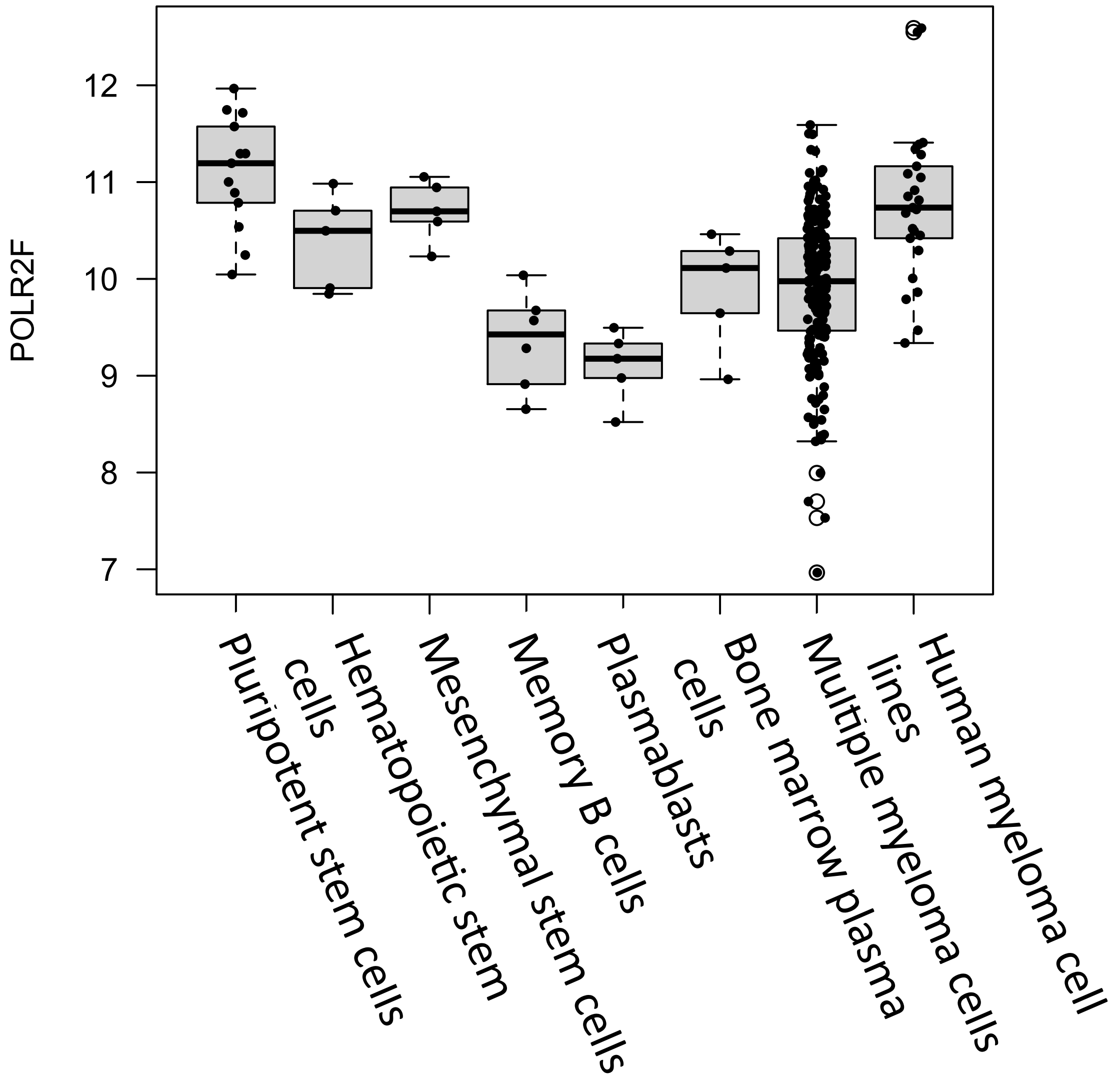

Figure S2

MFAP3L (205442_at***)***; microfibrillar-associated protein 3-like; **bad prognostic**; overexpressed in **mesenchymal stem cells** (MSC)

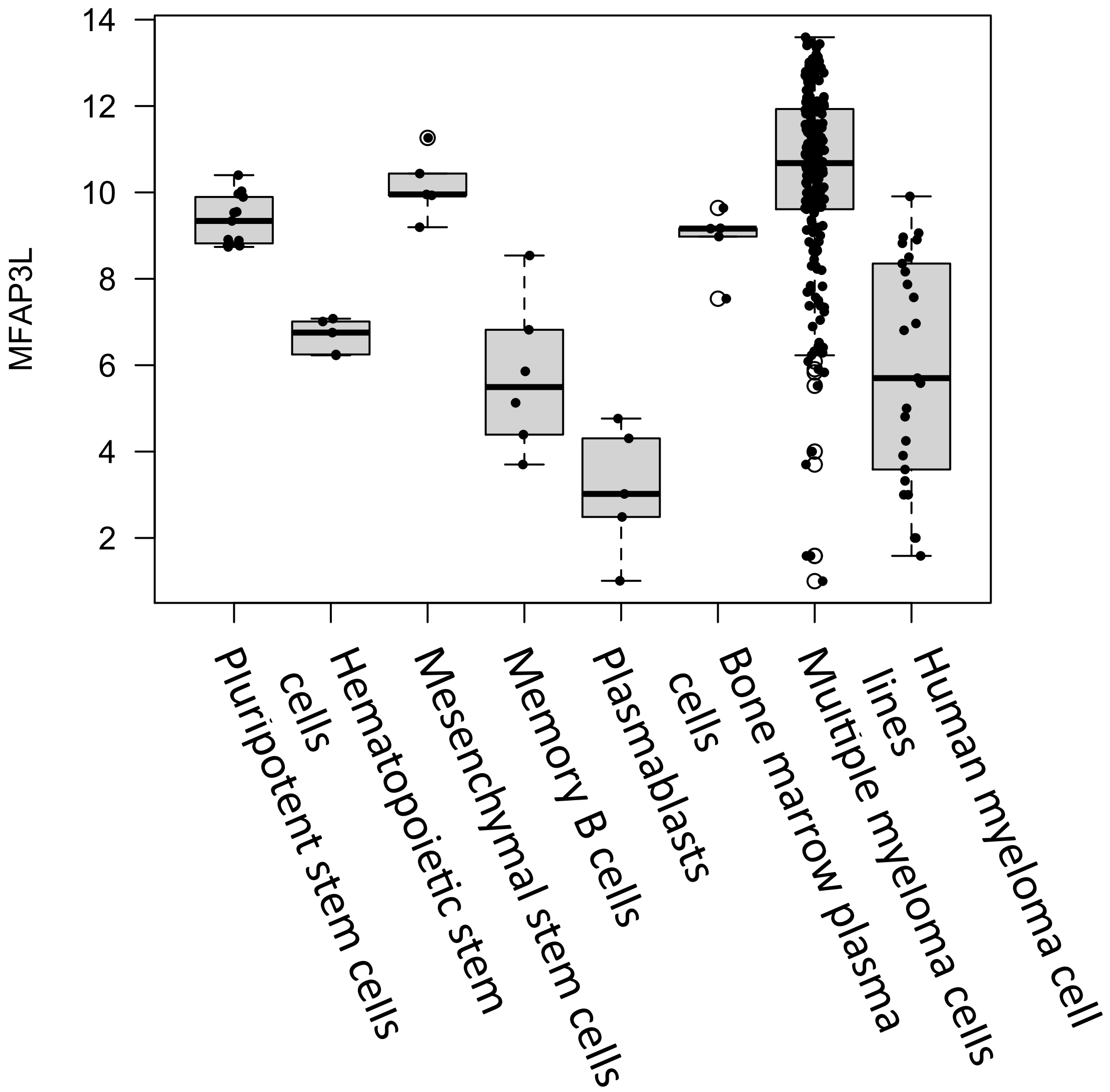

Supplement: Figure S2 — Gene expression signal of BAMBI, ROBO1, BCHE, GOLM1, PBX1, NANOS1, PKP2, NUDT11, POL2RF and MFAP3L. Gene expression was assayed using Affymetrix microarray in pluripotent stem cells (n = 13), hematopoietic stem cells (n = 5), mesenchymal stem cells (n = 5), memory b cells (n = 5), plasmablasts (n = 5), bone marrow plasma cells (n = 5), multiple myeloma cells (n = 206) and human myeloma cell lines (n = 25). Data are the log2 MAS5-normalized expression signal of each gene in the different cell populations. (PDF) [file pone.0042161.s002.pdf]
