# Supplementary material for: Identification of Pluripotent and Adult Stem Cell Genes Unrelated to Cell Cycle and Associated with Poor Prognosis in Multiple Myeloma
Source: PLoS One. 2012 Jul 31;7(7):e42161. doi: 10.1371/journal.pone.0042161 (PMC3409163; doi:10.1371/journal.pone.0042161)
Supplement: Table S2 — The 885 unique probe sets overexpressed in MMCs or in HMCLs compared to normal counterparts. (PDF) [file pone.0042161.s004.pdf]

Table S2. The 885 unique probe sets overexpressed in MMCs or in HMCLs compared to normal counterparts.

Probe sets	Name	Cytoband	Description
1555225_at	C1orf43	1q21.3	chromosome 1 open reading frame 43
1555226_s_at	C1orf43	1q21.3	chromosome 1 open reading frame 43
200837_at	BCAP31	Xq28	B-cell receptor-associated protein 31
201076_at	NHP2L1	22q13.2	NHP2 non-histone chromosome protein 2-like 1 (S. cerevisiae)
201119_s_at	COX8A	11q13.1	cytochrome c oxidase subunit 8A (ubiquitous)
201123_s_at	EIF5A	17p13.1	eukaryotic translation initiation factor 5A
201802_at	SLC29A1	6p21.1	solute carrier family 29 (nucleoside transporters), member 1
201927_s_at	PKP4	2q24.1	plakophilin 4
202159_at	FARSA	19p13.2	phenylalanyl-tRNA synthetase, alpha subunit
202322_s_at	GGPS1	1q42.3	geranylgeranyl diphosphate synthase 1
202480_s_at	DEDD	1q23.3	death effector domain containing
202846_s_at	PIGC	1q24.3	phosphatidylinositol glycan anchor biosynthesis, class C
203109_at	UBE2M	19q13.43	ubiquitin-conjugating enzyme E2M (UBC12 homolog, yeast)
203208_s_at	MTFR1	8q13.1	mitochondrial fission regulator 1
203235_at	THOP1	19p13.3	thimet oligopeptidase 1
203515_s_at	PMVK	1q21.3	phosphomevalonate kinase
203679_at	TMED1	19p13.2	transmembrane emp24 protein transport domain containing 1
204266_s_at	CHKA	11q13.2	choline kinase alpha
204587_at	SLC25A14	Xq26.1	solute carrier family 25 (mitochondrial carrier, brain), member 14
208784_s_at	KLHDC3	6p21.1	kelch domain containing 3
208799_at	PSMB5	14q11.2	proteasome (prosome, macropain) subunit, beta type, 5
209219_at	RDBP	HSCHR6_MHC_SSTO	RD RNA binding protein
209759_s_at	DCI	16p13.3	dodecenoyl-Coenzyme A delta isomerase (3,2 trans-enoyl-Coenzyme A isomerase)
210976_s_at	PFKM	12q13.11	phosphofructokinase, muscle
211855_s_at	SLC25A14	Xq26.1	solute carrier family 25 (mitochondrial carrier, brain), member 14
212541_at	FLAD1	1q21.3	FAD1 flavin adenine dinucleotide synthetase homolog (S. cerevisiae)
213222_at	PLCB1	20p12.3	phospholipase C, beta 1 (phosphoinositide-specific)
214383_x_at	KLHDC3	6p21.1	kelch domain containing 3
215667_x_at	LOC100132832	---	postmeiotic segregation increased 2-like 5-like /// PMS2 postmeiotic segregation increased 2 (S. cerevisiae)-like /// postmeiotic segregation increased 2-like 1 pseudogene /// postmeiotic segregation increased 2-like 2 pseudogene /// postmeiotic segregation increased 2-like 3 /// postmeiotic segregation increased 2-like 5
216194_s_at	TBCB	19q13.12	tubulin folding cofactor B
216602_s_at	FARSA	19p13.2	phenylalanyl-tRNA synthetase, alpha subunit
216843_x_at	PMS2L1	7q22.1	postmeiotic segregation increased 2-like 1 pseudogene
218046_s_at	MRPS16	10q22.2	mitochondrial ribosomal protein S16
218188_s_at	TIMM13	19p13.3	translocase of inner mitochondrial membrane 13 homolog (yeast)
218227_at	NUBP2	16p13.3	nucleotide binding protein 2 (MinD homolog, E. coli)
218481_at	EXOSC5	19q13.2	exosome component 5
218529_at	CD320	19p13.2	CD320 molecule
218695_at	EXOSC4	8q24.3	exosome component 4
219061_s_at	LAGE3	Xq28	L antigen family, member 3
219372_at	IFT81	12q24.11	intraflagellar transport 81 homolog (Chlamydomonas)
221270_s_at	QTRT1	19p13.2	queuine tRNA-ribosyltransferase 1
221579_s_at	NUDT3	6p21.31	nudix (nucleoside diphosphate linked moiety X)-type motif 3
221604_s_at	PEX16	11p11.2	peroxisomal biogenesis factor 16
221622_s_at	TMEM126B	11q14.1	transmembrane protein 126B
221759_at	G6PC3	17q21.31	glucose 6 phosphatase, catalytic, 3
221797_at	C17orf90	17q25.3	chromosome 17 open reading frame 90
221939_at	YIPF2	19p13.2	Yip1 domain family, member 2
222155_s_at	GPR172A	8q24.3	G protein-coupled receptor 172A
222736_s_at	TMEM38B	9q31.2	transmembrane protein 38B
222988_s_at	TMEM9	1q32.1	transmembrane protein 9
223989_s_at	REXO2	11q23.2	REX2, RNA exonuclease 2 homolog (S. cerevisiae)
225385_s_at	HNRPLL	2p22.1	heterogeneous nuclear ribonucleoprotein L-like
225421_at	PM20D2	6q15	peptidase M20 domain containing 2
225625_at	ALKBH2	12q24.11	alkB, alkylation repair homolog 2 (E. coli)
225638_at	C1orf31	1q42.2	chromosome 1 open reading frame 31
225647_s_at	CTSC	11q14.2	cathepsin C
225722_at	---	---	---
227369_at	SERBP1	1p31.3	SERPINE1 mRNA binding protein 1
227633_at	RHEB	7q36.1	Ras homolog enriched in brain
227936_at	TMEM68	8q12.1	transmembrane protein 68
228654_at	SPIN4	Xq11.1	spindlin family, member 4
229666_s_at	CSTF3	11p13	cleavage stimulation factor, 3' pre-RNA, subunit 3, 77kDa
232007_at	AGPAT5	8p23.1	1-acylglycerol-3-phosphate O-acyltransferase 5 (lysophosphatidic acid acyltransferase, epsilon)
236026_at	GPATCH2	1q41	G patch domain containing 2
238418_at	SLC35B4	7q33	solute carrier family 35, member B4
238662_at	ATPBD4	15q14	ATP binding domain 4
50374_at	C17orf90	17q25.3	chromosome 17 open reading frame 90
1555764_s_at	TIMM10	11q12.1	translocase of inner mitochondrial membrane 10 homolog (yeast)

1556096_s_at	UNC13C	15q21.3	unc-13 homolog C (C. elegans)
1560916_a_at	DPY19L1	7p14.2	dpy-19-like 1 (C. elegans)
202927_at	PIN1	19p13.2	peptidylprolyl cis/trans isomerase, NIMA-interacting 1
203022_at	RNASEH2A	19p13.2	ribonuclease H2, subunit A
203919_at	TCEA2	20q13.33	transcription elongation factor A (SII), 2
205194_at	PSPH	7p11.2	phosphoserine phosphatase
207628_s_at	WBSCR22	7q11.23	Williams Beuren syndrome chromosome region 22
208972_s_at	ATP5G1	17q21.32	ATP synthase, H+ transporting, mitochondrial F0 complex, subunit C1 (subunit 9)
209837_at	AP4M1	7q22.1	adaptor-related protein complex 4, mu 1 subunit
212215_at	PREPL	2p21	prolyl endopeptidase-like
214473_x_at	PMS2L3	7q11.23	postmeiotic segregation increased 2-like 3
214526_x_at	PMS2L1	7q22.1	postmeiotic segregation increased 2-like 1 pseudogene
214756_x_at	PMS2L1	7q22.1	postmeiotic segregation increased 2-like 1 pseudogene
216525_x_at	PMS2L3	7q11.23	postmeiotic segregation increased 2-like 3
217960_s_at	TOMM22	22q13.1	translocase of outer mitochondrial membrane 22 homolog (yeast)
223156_at	MRPS23	17q22	mitochondrial ribosomal protein S23
223272_s_at	C1orf57	1q42.2	chromosome 1 open reading frame 57
223917_s_at	SLC39A3	19p13.3	solute carrier family 39 (zinc transporter), member 3
227415_at	LOC283508	---	hypothetical protein LOC283508
227500_at	FBXL18	7p22.1	F-box and leucine-rich repeat protein 18
238914_at	DCC	18q21.2	deleted in colorectal carcinoma
242082_at	MMAB	12q24.11	methylmalonic aciduria (cobalamin deficiency) cblB type
203550_s_at	FAM189B	1q22	family with sequence similarity 189, member B
205190_at	PLS1	3q23	plastin 1 (I isoform)
210707_x_at	PMS2L11	7q11.23	postmeiotic segregation increased 2-like 11 pseudogene
217294_s_at	ENO1	1p36.23	enolase 1, (alpha)
220864_s_at	NDUFA13	19p13.11	NADH dehydrogenase (ubiquinone) 1 alpha subcomplex, 13
226328_at	KLF16	19p13.3	Kruppel-like factor 16
227669_at	BRP44	1q24.2	Brain protein 44
230032_at	OSGEPL1	2q32.2	O-sialoglycoprotein endopeptidase-like 1
1567080_s_at	CLN6	15q23	ceroid-lipofuscinosis, neuronal 6, late infantile, variant
1569969_a_at	UNC13C	15q21.3	unc-13 homolog C (C. elegans)
201405_s_at	COPS6	7q22.1	COP9 constitutive photomorphogenic homolog subunit 6 (Arabidopsis)
201577_at	NME1	17q21.33	non-metastatic cells 1, protein (NM23A) expressed in
201706_s_at	PEX19	1q23.2	peroxisomal biogenesis factor 19
203775_at	SLC25A13	7q21.3	solute carrier family 25, member 13 (citrin)
204521_at	C12orf24	12q24.11	chromosome 12 open reading frame 24
205224_at	SURF2	9q34.2	surfeit 2
209445_x_at	C7orf44	7p13	chromosome 7 open reading frame 44
212155_at	RNF187	1q42.13	ring finger protein 187
218011_at	UBL5	19p13.2	ubiquitin-like 5
220587_s_at	MLST8	16p13.3	MTOR associated protein, LST8 homolog (S. cerevisiae)
222347_at	LOC644450	---	hypothetical protein LOC644450
225201_s_at	MRPL14	6p21.1	mitochondrial ribosomal protein L14
227008_at	HDDC3	15q26.1	HD domain containing 3
230194_at	LRPPRC	2p21	leucine-rich PPR-motif containing
1554577_a_at	PSMD10	Xq22.3	proteasome (prosome, macropain) 26S subunit, non-ATPase, 10
1556095_at	UNC13C	15q21.3	unc-13 homolog C (C. elegans)
200885_at	RHOC	1p13.2	ras homolog gene family, member C
202382_s_at	GNPDA1	5q31.3	glucosamine-6-phosphate deaminase 1
202830_s_at	SLC37A4	11q23.3	solute carrier family 37 (glucose-6-phosphate transporter), member 4
204766_s_at	NUDT1	7p22.3	nudix (nucleoside diphosphate linked moiety X)-type motif 1
209068_at	HNRPDL	4q21.22	heterogeneous nuclear ribonucleoprotein D-like
211043_s_at	CLTB	5q35.2	clathrin, light chain (Lcb)
211752_s_at	NDUFS7	19p13.3	NADH dehydrogenase (ubiquinone) Fe-S protein 7, 20kDa (NADH-coenzyme Q reductase)
212792_at	DPY19L1	7p14.2	dpy-19-like 1 (C. elegans)
213504_at	COPS6	7q22.1	COP9 constitutive photomorphogenic homolog subunit 6 (Arabidopsis)
213893_x_at	PMS2L5	7q11.23	postmeiotic segregation increased 2-like 5
218507_at	C7orf68	7q32.1	chromosome 7 open reading frame 68
218654_s_at	MRPS33	7q34	mitochondrial ribosomal protein S33
218671_s_at	ATPIF1	1p35.3	ATPase inhibitory factor 1
218689_at	FANCF	11p14.3	Fanconi anemia, complementation group F
218908_at	ASPSCR1	17q25.3	alveolar soft part sarcoma chromosome region, candidate 1
223306_at	EBPL	13q14.2	emopamil binding protein-like
223461_at	TBC1D7	6p24.1	TBC1 domain family, member 7
223649_s_at	SLC25A39	17q21.31	solute carrier family 25, member 39
226414_s_at	ANAPC11	17q25.3	anaphase promoting complex subunit 11
228879_at	SNORD104	17q23.3	small nucleolar RNA, C/D box 104
229371_at	---	---	---
233350_s_at	TEX264	3p21.2	testis expressed 264
238529_at	LOC730631	---	Hypothetical LOC730631
243661_at	ZNF273	7q11.21	zinc finger protein 273
58696_at	EXOSC4	8q24.3	exosome component 4
1554417_s_at	APH1A	1q21.2	anterior pharynx defective 1 homolog A (C. elegans)
203219_s_at	APRT	16q24.3	adenine phosphoribosyltransferase

204004_at	PAWR	12q21.2	PRKC, apoptosis, WT1, regulator
206441_s_at	COMMD4	15q24.2	COMM domain containing 4
213357_at	GTF2H5	6q25.3	general transcription factor IIH, polypeptide 5
218159_at	DDRGK1	20p13	DDRGK domain containing 1
225646_at	CTSC	11q14.2	cathepsin C
225961_at	KLHDC5	12p11.22	kelch domain containing 5
226963_at	BTF3L4	1p32.3	basic transcription factor 3-like 4
238021_s_at	CRNDE	---	colorectal neoplasia differentially expressed (non-protein coding)
242488_at	---	---	---
91952_at	DCAF15	19p13.12	DDB1 and CUL4 associated factor 15
207039_at	CDKN2A	9p21.3	cyclin-dependent kinase inhibitor 2A (melanoma, p16, inhibits CDK4)
212765_at	CAMSAP1L1	1q32.1	calmodulin regulated spectrin-associated protein 1-like 1
218275_at	SLC25A10	17q25.3	solute carrier family 25 (mitochondrial carrier; dicarboxylate transporter), member 10
221712_s_at	WDR74	11q12.3	WD repeat domain 74
222317_at	PDE3B	11p15.2	phosphodiesterase 3B, cGMP-inhibited
223126_s_at	C1orf21	1q25.3	chromosome 1 open reading frame 21
224298_s_at	UBAC2	13q32.3	UBA domain containing 2
225386_s_at	HNRPLL	2p22.1	heterogeneous nuclear ribonucleoprotein L-like
230399_at	---	---	---
232271_at	HNF4G	8q21.11	hepatocyte nuclear factor 4, gamma
239010_at	FLJ39632	---	Hypothetical LOC642477
242069_at	CBX5	12q13.13	chromobox homolog 5 (HP1 alpha homolog, Drosophila)
1555487_a_at	ACTR3B	7q36.1	ARP3 actin-related protein 3 homolog B (yeast) /// similar to actin-related protein 3-beta
202961_s_at	ATP5J2	7q22.1	ATP synthase, H+ transporting, mitochondrial F0 complex, subunit F2
203458_at	SPR	2p13.2	sepiapterin reductase (7,8-dihydrobiopterin:NADP+ oxidoreductase)
203739_at	ZNF217	20q13.2	zinc finger protein 217
204133_at	RRP9	3p21.2	ribosomal RNA processing 9, small subunit (SSU) processome component, homolog (yeast)
204331_s_at	MRPS12	19q13.2	mitochondrial ribosomal protein S12
205543_at	HSPA4L	4q28.1	heat shock 70kDa protein 4-like
206545_at	CD28	2q33.2	CD28 molecule
208226_x_at	ADAM22	7q21.12	ADAM metallopeptidase domain 22
208227_x_at	ADAM22	7q21.12	ADAM metallopeptidase domain 22
209478_at	STRA13	17q25.3	stimulated by retinoic acid 13 homolog (mouse)
210115_at	RPL39L	3q27.3	ribosomal protein L39-like
210378_s_at	SSNA1	9q34.3	Sjogren syndrome nuclear autoantigen 1
213906_at	MYBL1	8q13.1	v-myb myeloblastosis viral oncogene homolog (avian)-like 1
218220_at	C12orf10	12q13.13	chromosome 12 open reading frame 10
218290_at	PLEKHJ1	19p13.3	pleckstrin homology domain containing, family J member 1
218816_at	LRRC1	6p12.1	leucine rich repeat containing 1
220144_s_at	ANKRD5	20p12.2	ankyrin repeat domain 5
221822_at	CCDC101	16p11.2	coiled-coil domain containing 101
223530_at	TDRKH	1q21.3	tudor and KH domain containing
224129_s_at	DPY30	2p22.3	dpy-30 homolog (C. elegans)
226483_at	TMEM68	8q12.1	transmembrane protein 68
226813_at	C1orf57	1q42.2	chromosome 1 open reading frame 57
242881_x_at	---	---	---
200844_s_at	PRDX6	1q25.1	peroxiredoxin 6
201079_at	SYNGR2	17q25.3	synaptogyrin 2
202024_at	ASNA1	19p13.2	arsA arsenite transporter, ATP-binding, homolog 1 (bacterial)
205172_x_at	CLTB	5q35.2	clathrin, light chain (Lcb)
205826_at	MYOM2	8p23.3	myomesin (M-protein) 2, 165kDa
208237_x_at	ADAM22	7q21.12	ADAM metallopeptidase domain 22
208967_s_at	AK2	1p35.1	adenylate kinase 2
209424_s_at	AMACR	5p13.2	alpha-methylacyl-CoA racemase
209425_at	AMACR	5p13.2	alpha-methylacyl-CoA racemase
209731_at	NTHL1	16p13.3	nth endonuclease III-like 1 (E. coli)
212727_at	DLG3	Xq13.1	discs, large homolog 3 (Drosophila)
218336_at	PFDN2	1q23.3	prefoldin subunit 2
219274_at	TSPAN12	7q31.31	tetraspanin 12
223805_at	OSBPL6	2q31.2	oxysterol binding protein-like 6
225454_at	CCDC124	19p13.11	coiled-coil domain containing 124
228400_at	SHROOM3	4q21.1	shroom family member 3
1554057_at	LOC645676	---	hypothetical LOC645676
1558164_s_at	PEX13	2p16.1	peroxisomal biogenesis factor 13
206284_x_at	CLTB	5q35.2	clathrin, light chain (Lcb)
206506_s_at	SUPT3H	6p21.1	suppressor of Ty 3 homolog (S. cerevisiae)
206698_at	XK	Xp21.1	X-linked Kx blood group (McLeod syndrome)
209213_at	CBR1	21q22.12	carbonyl reductase 1
209864_at	FRAT2	10q24.1	frequently rearranged in advanced T-cell lymphomas 2
210135_s_at	SHOX2	3q25.32	short stature homeobox 2
214582_at	PDE3B	11p15.2	phosphodiesterase 3B, cGMP-inhibited
216593_s_at	LOC100289848	---	similar to phosphatidylinositol glycan, class C /// phosphatidylinositol glycan anchor biosynthesis, class C

217771_at	GOLM1	9q21.33	golgi membrane protein 1
222994_at	PRDX5	11q13.1	peroxiredoxin 5
225193_at	---	---	---
225308_s_at	TANC1	2q24.2	tetratricopeptide repeat, ankyrin repeat and coiled-coil containing 1
226458_at	---	---	---
226727_at	CISD3	17q12	CDGSH iron sulfur domain 3
239989_at	CNTLN	9p22.2	centlein, centrosomal protein
1556194_a_at	---	---	---
201563_at	SORD	15q21.1	sorbitol dehydrogenase
201929_s_at	PKP4	2q24.1	plakophilin 4
203234_at	UPP1	7p12.3	uridine phosphorylase 1
203452_at	B3GAT3	11q12.3	beta-1,3-glucuronyltransferase 3 (glucuronosyltransferase I)
204068_at	STK3	Xq26.2	serine/threonine kinase 3 (STE20 homolog, yeast)
207831_x_at	DHPS	19p13.2	deoxyhypusine synthase
209426_s_at	AMACR	5p13.2	alpha-methylacyl-CoA racemase
209567_at	RRS1	8q13.1	RRS1 ribosome biogenesis regulator homolog (S. cerevisiae)
211471_s_at	RAB36	22q11.22	RAB36, member RAS oncogene family
214596_at	CHRM3	1q43	cholinergic receptor, muscarinic 3
214706_at	ZNF200	16p13.3	zinc finger protein 200
217485_x_at	PMS2L1	7q22.1	postmeiotic segregation increased 2-like 1 pseudogene
218688_at	DAK	11q12.2	dihydroxyacetone kinase 2 homolog (S. cerevisiae)
220942_x_at	FAM162A	3q21.1	family with sequence similarity 162, member A
224855_at	LEFTY1	1q42.12	left-right determination factor 1 /// pyrroline-5-carboxylate reductase family, member 2
225861_at	C16orf14	---	chromosome 16 open reading frame 14
225997_at	MOBKL1A	4q13.3	MOB1, Mps One Binder kinase activator-like 1A (yeast)
231882_at	FLJ39632	---	hypothetical LOC642477
232101_s_at	PIGN	18q21.33	phosphatidylinositol glycan anchor biosynthesis, class N
242546_at	FLJ39632	---	hypothetical LOC642477
1560371_at	LOC401321	---	hypothetical LOC401321
204756_at	MAP2K5	15q23	mitogen-activated protein kinase kinase 5
205164_at	GCAT	22q13.1	glycine C-acetyltransferase (2-amino-3-ketobutyrate coenzyme A ligase)
208369_s_at	GCDH	19p13.2	glutaryl-Coenzyme A dehydrogenase
214958_s_at	TMC6	17q25.3	transmembrane channel-like 6
219431_at	ARHGAP10	4q31.23	Rho GTPase activating protein 10
227103_s_at	ECE2	3q27.1	endothelin converting enzyme 2
1553715_s_at	C16orf14	---	chromosome 16 open reading frame 14
1557331_at	POLR1B	2q13	polymerase (RNA) I polypeptide B, 128kDa
208907_s_at	MRPS18B	HSCHR6_MHC_MCF	mitochondrial ribosomal protein S18B
209449_at	LSM2	HSCHR6_MHC_APD	LSM2 homolog, U6 small nuclear RNA associated (S. cerevisiae)
217871_s_at	MIF	22q11.23	macrophage migration inhibitory factor (glycosylation-inhibiting factor)
217894_at	KCTD3	1q41	potassium channel tetramerisation domain containing 3
218996_at	TFPT	19q13.42	TCF3 (E2A) fusion partner (in childhood Leukemia)
220459_at	MCM3APAS	21q22.3	MCM3AP antisense RNA (non-protein coding)
223993_s_at	CNIH4	1q42.11	cornichon homolog 4 (Drosophila)
224823_at	MYLK	3q21.1	myosin light chain kinase
230298_at	MBLAC2	5q14.3	metallo-beta-lactamase domain containing 2
233746_x_at	C15orf63	15q15.3	chromosome 15 open reading frame 63 /// small EDRK-rich factor 2
1559633_a_at	CHRM3	1q43	cholinergic receptor, muscarinic 3
1568612_at	GABRG2	5q34	gamma-aminobutyric acid (GABA) A receptor, gamma 2
202555_s_at	MYLK	3q21.1	myosin light chain kinase
207543_s_at	P4HA1	10q22.1	prolyl 4-hydroxylase, alpha polypeptide I
213321_at	BCKDHB	6q14.1	branched chain keto acid dehydrogenase E1, beta polypeptide
214614_at	MNX1	7q36.3	motor neuron and pancreas homeobox 1
218728_s_at	CNIH4	1q42.11	cornichon homolog 4 (Drosophila)
225904_at	C1orf96	1q42.13	chromosome 1 open reading frame 96
227421_at	C21orf57	21q22.3	chromosome 21 open reading frame 57
227864_s_at	FAM125A	19p13.11	family with sequence similarity 125, member A
231102_at	CROT	7q21.12	carnitine O-octanoyltransferase
231319_x_at	KIF9	3p21.31	kinesin family member 9
241749_at	MURC	9q31.1	muscle-related coiled-coil protein
202785_at	NDUFA7	19p13.2	NADH dehydrogenase (ubiquinone) 1 alpha subcomplex, 7, 14.5kDa
202802_at	DHPS	19p13.2	deoxyhypusine synthase
203819_s_at	IGF2BP3	7p15.3	insulin-like growth factor 2 mRNA binding protein 3
207480_s_at	MEIS2	15q14	Meis homeobox 2
217289_s_at	SLC37A4	11q23.3	solute carrier family 37 (glucose-6-phosphate transporter), member 4
219733_s_at	SLC27A5	19q13.43	solute carrier family 27 (fatty acid transporter), member 5
225657_at	LOC152217	---	hypothetical LOC152217
226453_at	RNASEH2C	11q13.1	ribonuclease H2, subunit C
228066_at	C17orf96	17q12	chromosome 17 open reading frame 96
239552_at	VWDE	7p21.3	von Willebrand factor D and EGF domains
240185_at	LOC100147773	---	hypothetical LOC100147773
203025_at	ARD1A	---	ARD1 homolog A, N-acetyltransferase (S. cerevisiae)
205677_s_at	DLEU1	13q14.2	deleted in lymphocytic leukemia 1 (non-protein coding)
209507_at	RPA3	7p21.3	replication protein A3, 14kDa
209942_x_at	MAGEA3	Xq28	melanoma antigen family A, 3

213411_at	---	---	---
214612_x_at	MAGEA6	Xq28	melanoma antigen family A, 6
215812_s_at	LOC653562	---	similar to solute carrier family 6 member 8 /// solute carrier family 6 (neurotransmitter transporter, creatine), member 10 (pseudogene) /// solute carrier family 6 (neurotransmitter transporter, creatine), member 8
219072_at	BCL7C	16p11.2	B-cell CLL/lymphoma 7C
221692_s_at	MRPL34	19p13.11	mitochondrial ribosomal protein L34
226609_at	DCBLD1	6q22.1	discoidin, CUB and LCCL domain containing 1
226780_s_at	C7orf55	7q34	chromosome 7 open reading frame 55
230769_at	DENND2C	1p13.2	DENN/MADD domain containing 2C
204483_at	ENO3	17p13.2	enolase 3 (beta, muscle)
206950_at	SCN9A	2q24.3	sodium channel, voltage-gated, type IX, alpha subunit
210587_at	INHBE	12q13.3	inhibin, beta E
213079_at	TSR2	Xp11.22	TSR2, 20S rRNA accumulation, homolog (S. cerevisiae)
221058_s_at	CKLF	16q21	chemokine-like factor
223253_at	EPDR1	7p14.1	ependymin related protein 1 (zebrafish)
224448_s_at	C6orf125	6p21.31	chromosome 6 open reading frame 125
224666_at	NSMCE1	16p12.1	non-SMC element 1 homolog (S. cerevisiae)
227256_at	USP31	16p12.2	ubiquitin specific peptidase 31
228355_s_at	NDUFAF2	5q12.1	NADH dehydrogenase (ubiquinone) 1 alpha subcomplex, assembly factor 2
230434_at	PHOSPHO2	2q31.1	phosphatase, orphan 2
231423_s_at	ANKRD16	10p15.1	ankyrin repeat domain 16
206070_s_at	EPHA3	3p11.1	EPH receptor A3
208704_x_at	APLP2	11q24.3	amyloid beta (A4) precursor-like protein 2
208760_at	UBE2I	16p13.3	Ubiquitin-conjugating enzyme E2I (UBC9 homolog, yeast)
209781_s_at	KHDRBS3	8q24.23	KH domain containing, RNA binding, signal transduction associated 3
220993_s_at	GPR63	6q16.1	G protein-coupled receptor 63
225882_at	SLC35B4	7q33	solute carrier family 35, member B4
226285_at	CAPRIN1	11p13	cell cycle associated protein 1
226460_at	FNIP2	4q32.1	folliculin interacting protein 2
227174_at	WDR72	15q21.3	WD repeat domain 72
228619_x_at	TIPRL	1q24.2	TIP41, TOR signaling pathway regulator-like (S. cerevisiae)
229796_at	SIX4	14q23.1	SIX homeobox 4
230257_s_at	TSEN15	1q25.3	tRNA splicing endonuclease 15 homolog (S. cerevisiae)
238022_at	CRNDE	---	colorectal neoplasia differentially expressed (non-protein coding)
240280_at	UFSP1	7q22.1	UFM1-specific peptidase 1 (non-functional)
1553587_a_at	POLE4	2p12	polymerase (DNA-directed), epsilon 4 (p12 subunit)
203771_s_at	BLVRA	7p13	biliverdin reductase A
205830_at	CLGN	4q31.1	calmegin
207307_at	HTR2C	Xq23	5-hydroxytryptamine (serotonin) receptor 2C
207663_x_at	GAGE3	Xp11.23	G antigen 3
219260_s_at	C17orf81	17p13.1	chromosome 17 open reading frame 81
220040_x_at	ZC4H2	Xq11.2	zinc finger, C4H2 domain containing
221558_s_at	LEF1	4q25	lymphoid enhancer-binding factor 1
226231_at	---	---	---
228116_at	---	---	---
238728_at	---	---	---
201221_s_at	SNRNP70	19q13.33	small nuclear ribonucleoprotein 70kDa (U1)
208248_x_at	APLP2	11q24.3	amyloid beta (A4) precursor-like protein 2
210467_x_at	MAGEA12	Xq28	melanoma antigen family A, 12
218201_at	NDUFB2	7q34	NADH dehydrogenase (ubiquinone) 1 beta subcomplex, 2, 8kDa
221849_s_at	DCAF15	19p13.12	DDB1 and CUL4 associated factor 15
224387_at	COMMD5	8q24.3	COMM domain containing 5
225924_at	FNIP2	4q32.1	folliculin interacting protein 2
226558_at	LOC389834	---	ankyrin repeat domain 57 pseudogene
231952_at	---	---	---
239069_s_at	---	---	---
202975_s_at	RHOBTB3	5q15	Rho-related BTB domain containing 3
207325_x_at	MAGEA1	Xq28	melanoma antigen family A, 1 (directs expression of antigen MZ2-E)
209511_at	POLR2F	22q13.1	polymerase (RNA) II (DNA directed) polypeptide F
213587_s_at	ATP6V0E2	7q36.1	ATPase, H+ transporting V0 subunit e2
214642_x_at	MAGEA5	Xq28	melanoma antigen family A, 5
219161_s_at	CKLF	16q21	chemokine-like factor
221526_x_at	PARD3	10p11.21	par-3 partitioning defective 3 homolog (C. elegans)
224890_s_at	C7orf59	7q22.1	chromosome 7 open reading frame 59
225202_at	RHOBTB3	5q15	Rho-related BTB domain containing 3
235443_at	LOC100131067	---	hypothetical protein LOC100131067
203820_s_at	IGF2BP3	7p15.3	insulin-like growth factor 2 mRNA binding protein 3
204066_s_at	AGAP1	2q37.2	ArfGAP with GTPase domain, ankyrin repeat and PH domain 1
204238_s_at	C6orf108	6p21.1	chromosome 6 open reading frame 108
208155_x_at	GAGE1	Xp11.23	G antigen 1 /// G antigen 12F /// G antigen 12G /// G antigen 12I /// G antigen 12J /// G antigen 4 /// G antigen 5 /// G antigen 6 /// G antigen 7
211674_x_at	CTAG1A	Xq28	cancer/testis antigen 1A /// cancer/testis antigen 1B
213012_at	NEDD4	15q21.3	neural precursor cell expressed, developmentally down-regulated 4
221272_s_at	C1orf21	1q25.3	chromosome 1 open reading frame 21
229415_at	CYCS	7p15.3	cytochrome c, somatic

229517_at	PTPDC1	9q22.32	protein tyrosine phosphatase domain containing 1
230738_at	LOC730631	---	Hypothetical LOC730631
1557070_at	---	---	---
1568780_at	LOC649305	---	hypothetical LOC649305
202903_at	LSM5	7p14.3	LSM5 homolog, U6 small nuclear RNA associated (S. cerevisiae)
204364_s_at	REEP1	2p11.2	receptor accessory protein 1
208235_x_at	GAGE12F	Xp11.23	G antigen 12F /// G antigen 12G /// G antigen 12I /// G antigen 7
211025_x_at	COX5B	2q11.2	cytochrome c oxidase subunit Vb
214079_at	DHRS2	14q11.2	dehydrogenase/reductase (SDR family) member 2
219345_at	BOLA1	1q21.2	bolA homolog 1 (E. coli)
225359_at	DNAJC19	3q26.33	DnaJ (Hsp40) homolog, subfamily C, member 19
225464_at	FRMD6	14q22.1	FERM domain containing 6
227798_at	SMAD1	4q31.21	SMAD family member 1
228053_s_at	TOMM5	9p13.2	translocase of outer mitochondrial membrane 5 homolog (yeast)
230158_at	DPY19L2	7p14.2	dpy-19-like 2 (C. elegans)
236514_at	ACOT8	20q13.12	acyl-CoA thioesterase 8
1557174_a_at	---	---	---
201259_s_at	SYPL1	7q22.3	synaptophysin-like 1
207714_s_at	SERPINH1	11q13.5	serpin peptidase inhibitor, clade H (heat shock protein 47), member 1, (collagen binding protein 1)
215733_x_at	CTAG2	Xq28	cancer/testis antigen 2
225802_at	TOP1MT	8q24.3	topoisomerase (DNA) I, mitochondrial
226360_at	ZNRF3	22q12.1	zinc and ring finger 3
228977_at	LOC729680	---	hypothetical protein LOC729680
229826_at	LOC440957	---	similar to CG32736-PA
235147_at	FLJ32063	---	Hypothetical LOC150538
236989_at	---	---	---
202932_at	YES1	18p11.32	v-yes-1 Yamaguchi sarcoma viral oncogene homolog 1
203627_at	IGF1R	15q26.3	insulin-like growth factor 1 receptor
204979_s_at	SH3BGR	21q22.2	SH3 domain binding glutamic acid-rich protein
205354_at	GAMT	19p13.3	guanidinoacetate N-methyltransferase
213194_at	ROBO1	3p12.2	roundabout, axon guidance receptor, homolog 1 (Drosophila)
219474_at	C3orf52	3q13.2	chromosome 3 open reading frame 52
225065_x_at	NCRNA00188	17p11.2	non-protein coding RNA 188
226433_at	RNF157	17q25.1	ring finger protein 157
227921_at	---	---	---
232010_at	FSTL5	4q32.2	follistatin-like 5
64900_at	FLJ22167	---	hypothetical protein FLJ22167
1557215_at	---	---	---
1562099_at	---	---	---
200085_s_at	TCEB2	16p13.3	transcription elongation factor B (SIII), polypeptide 2 (18kDa, elongin B)
202260_s_at	STXBP1	9q34.11	syntaxin binding protein 1
206394_at	MYBPC2	19q13.33	myosin binding protein C, fast type
207717_s_at	PKP2	12p11.21	plakophilin 2
207874_s_at	CFHR4	1q31.3	complement factor H-related 4
210993_s_at	SMAD1	4q31.21	SMAD family member 1
212353_at	SULF1	8q13.2	sulfatase 1
222772_at	MYEF2	15q21.1	myelin expression factor 2
225189_s_at	RAPH1	2q33.2	Ras association (RalGDS/AF-6) and pleckstrin homology domains 1
228365_at	CPNE8	12q12	copine VIII
235532_at	---	---	---
202431_s_at	MYC	8q24.21	v-myc myelocytomatosis viral oncogene homolog (avian)
202976_s_at	RHOBTB3	5q15	Rho-related BTB domain containing 3
210125_s_at	BANF1	11q13.1	barrier to autointegration factor 1
214857_at	---	---	---
218862_at	ASB13	10p15.1	ankyrin repeat and SOCS box-containing 13
223451_s_at	CKLF	16q21	chemokine-like factor
241367_at	TEX19	17q25.3	testis expressed 19
1558139_at	FLJ39632	---	hypothetical LOC642477 /// hypothetical LOC400879
1569469_a_at	LHX8	1p31.1	LIM homeobox 8
209867_s_at	LPHN3	4q13.1	latrophilin 3
210546_x_at	CTAG1A	Xq28	cancer/testis antigen 1A /// cancer/testis antigen 1B
211425_x_at	SSX4	Xp11.23	synovial sarcoma, X breakpoint 4 /// synovial sarcoma, X breakpoint 4B
212148_at	PBX1	1q23.3	pre-B-cell leukemia homeobox 1
212891_s_at	GADD45GIP1	19p13.2	growth arrest and DNA-damage-inducible, gamma interacting protein 1
218161_s_at	CLN6	15q23	ceroid-lipofuscinosis, neuronal 6, late infantile, variant
218326_s_at	LGR4	11p14.1	leucine-rich repeat-containing G protein-coupled receptor 4
218847_at	IGF2BP2	3q27.2	insulin-like growth factor 2 mRNA binding protein 2
224741_x_at	GAS5	1q25.1	growth arrest-specific 5 (non-protein coding)
224841_x_at	GAS5	1q25.1	growth arrest-specific 5 (non-protein coding)
224955_at	TEAD1	11p15.3	TEA domain family member 1 (SV40 transcriptional enhancer factor)
227579_at	---	---	---
230701_x_at	KIF9	3p21.31	kinesin family member 9
1553972_a_at	CBS	21q22.3	cystathionine-beta-synthase
206435_at	B4GALNT1	12q13.3	beta-1,4-N-acetyl-galactosaminyl transferase 1

209066_x_at	UQCRB	8q22.1	ubiquinol-cytochrome c reductase binding protein
212254_s_at	DST	6p12.1	dystonin
212816_s_at	CBS	21q22.3	cystathionine-beta-synthase
224916_at	TMEM173	5q31.2	transmembrane protein 173
227688_at	LRCH2	Xq23	leucine-rich repeats and calponin homology (CH) domain containing 2
229090_at	LOC220930	---	hypothetical LOC220930
206640_x_at	GAGE12C	Xp11.23	G antigen 12C /// G antigen 12D /// G antigen 12E /// G antigen 12F /// G antigen 12G /// G antigen 12H /// G antigen 12I /// G antigen 2A /// G antigen 2C /// G antigen 4 /// G antigen 5 /// G antigen 6 /// G antigen 7
218644_at	PLEK2	14q23.3	pleckstrin 2
224929_at	TMEM173	5q31.2	transmembrane protein 173
231914_at	NUDT14	14q32.33	nudix (nucleoside diphosphate linked moiety X)-type motif 14
239155_at	---	---	---
239319_at	LOC728342	---	Hypothetical protein LOC728342
239834_at	---	---	---
205433_at	BCHE	3q26.1	butyrylcholinesterase
206204_at	GRB14	2q24.3	growth factor receptor-bound protein 14
210437_at	MAGEA9	Xq28	melanoma antigen family A, 9 /// melanoma antigen family A, 9B
210852_s_at	AASS	7q31.32	aminoadipate-semialdehyde synthase
221884_at	MECOM	3q26.2	MDS1 and EVI1 complex locus
223378_at	GLIS2	16p13.3	GLIS family zinc finger 2
225603_s_at	C8orf83	8q22.1	chromosome 8 open reading frame 83
226545_at	CD109	6q13	CD109 molecule
227053_at	PACSIN1	6p21.31	protein kinase C and casein kinase substrate in neurons 1
229464_at	MYEF2	15q21.1	myelin expression factor 2
244080_at	LOC100287039	---	hypothetical protein LOC100287039
244114_x_at	---	---	---
204560_at	FKBP5	6p21.31	FK506 binding protein 5
205450_at	PHKA1	Xq13.2	phosphorylase kinase, alpha 1 (muscle)
228049_x_at	---	---	---
228523_at	NANOS1	10q26.11	nanos homolog 1 (Drosophila)
217339_x_at	CTAG1A	Xq28	cancer/testis antigen 1A /// cancer/testis antigen 1B
225731_at	ANKRD50	4q28.1	ankyrin repeat domain 50
235207_at	---	---	---
203917_at	CXADR	21q21.1	coxsackie virus and adenovirus receptor
208791_at	CLU	8p21.1	clusterin
224560_at	TIMP2	17q25.3	TIMP metallopeptidase inhibitor 2
226374_at	---	---	---
226781_at	C7orf55	7q34	chromosome 7 open reading frame 55
205551_at	SV2B	15q26.1	synaptic vesicle glycoprotein 2B
205774_at	F12	5q35.3	coagulation factor XII (Hageman factor)
209325_s_at	RGS16	1q25.3	regulator of G-protein signaling 16
210094_s_at	PARD3	10p11.21	par-3 partitioning defective 3 homolog (C. elegans)
213155_at	WSCD1	17p13.2	WSC domain containing 1
214254_at	MAGEA4	Xq28	melanoma antigen family A, 4
220771_at	LOC51152	---	melanoma antigen
225354_s_at	SH3BGRL2	6q14.1	SH3 domain binding glutamic acid-rich protein like 2
226837_at	SPRED1	15q14	sprouty-related, EVH1 domain containing 1
1553441_at	CNTNAP4	16q23.1	contactin associated protein-like 4
207739_s_at	GAGE1	Xp11.23	G antigen 1 /// G antigen 12F /// G antigen 12G /// G antigen 12I /// G antigen 12J /// G antigen 2A /// G antigen 2B /// G antigen 2C /// G antigen 2D /// G antigen 2E /// G antigen 3 /// G antigen 4 /// G antigen 5 /// G antigen 6 /// G antigen 7 /// G antigen 8
219738_s_at	PCDH9	13q21.32	protocadherin 9
222810_s_at	RASAL2	1q25.2	RAS protein activator like 2
226707_at	NAPRT1	8q24.3	nicotinate phosphoribosyltransferase domain containing 1
236741_at	WDR72	15q21.3	WD repeat domain 72
215016_x_at	DST	6p12.1	dystonin
218029_at	FAM65A	16q22.1	family with sequence similarity 65, member A
223949_at	TMPRSS3	21q22.3	transmembrane protease, serine 3
229900_at	CD109	6q13	CD109 molecule
233814_at	EFNA5	5q21.3	ephrin-A5
1555579_s_at	PTPRM	18p11.23	protein tyrosine phosphatase, receptor type, M
202022_at	ALDOC	17q11.2	aldolase C, fructose-bisphosphate
203773_x_at	BLVRA	7p13	biliverdin reductase A
203854_at	CFI	4q25	complement factor I
207808_s_at	PROS1	3q11.1	protein S (alpha)
210480_s_at	MYO6	6q14.1	myosin VI
212686_at	PPM1H	12q14.2	protein phosphatase 1H (PP2C domain containing)
214608_s_at	EYA1	8q13.3	eyes absent homolog 1 (Drosophila)
228274_at	SDSL	12q24.13	serine dehydratase-like
1554459_s_at	CFHR3	1q31.3	complement factor H-related 3
210394_x_at	SSX4	Xp11.23	synovial sarcoma, X breakpoint 4 /// synovial sarcoma, X breakpoint 4B
210497_x_at	SSX2	Xp11.22	synovial sarcoma, X breakpoint 2
211432_s_at	TYRO3	15q15.1	TYRO3 protein tyrosine kinase
212151_at	PBX1	1q23.3	pre-B-cell leukemia homeobox 1

219368_at	NAP1L2	Xq13.2	nucleosome assembly protein 1-like 2
225481_at	FRMD6	14q22.1	FERM domain containing 6
227491_at	---	---	---
227917_at	FAM85A	---	family with sequence similarity 85, member A
1555564_a_at	CFI	4q25	complement factor I
201841_s_at	HSPB1	7q11.23	heat shock 27kDa protein 1
202620_s_at	PLOD2	3q24	procollagen-lysine, 2-oxoglutarate 5-dioxygenase 2
204749_at	NAP1L3	Xq21.32	nucleosome assembly protein 1-like 3
205123_s_at	TMEFF1	9q31.1	transmembrane protein with EGF-like and two follistatin-like domains 1
207156_at	HIST1H2AG	6p22.1	histone cluster 1, H2ag
233514_x_at	TEX11	Xq13.1	testis expressed 11
241224_x_at	DSCR8	21q22.13	Down syndrome critical region gene 8
207712_at	BAGE	---	B melanoma antigen
209210_s_at	FERMT2	14q22.1	fermitin family homolog 2 (Drosophila)
228171_s_at	PLEKHG4	16q22.1	pleckstrin homology domain containing, family G (with RhoGef domain) member 4
208451_s_at	C4A	HSCHR6_MHC_QBL	complement component 4A (Rodgers blood group) /// complement component 4B (Chido blood group) /// similar to Complement component 4A (Rodgers blood group) /// similar to complement component 4A (Rodgers blood group)
219702_at	PLAC1	Xq26.3	placenta-specific 1
224970_at	NFIA	1p31.3	nuclear factor I/A
229337_at	USP2	11q23.3	ubiquitin specific peptidase 2
235902_at	---	---	---
1558605_at	---	---	---
205529_s_at	RUNX1T1	8q21.3	runt-related transcription factor 1; translocated to, 1 (cyclin D-related)
209465_x_at	PTN	7q33	pleiotrophin
213249_at	FBXL7	5p15.1	F-box and leucine-rich repeat protein 7
220445_s_at	CSAG2	Xq28	CSAG family, member 2 /// CSAG family, member 3
224763_at	RPL37	5p13.1	Ribosomal protein L37
233413_at	---	---	---
235759_at	---	---	---
242135_at	LOC100289097	---	hypothetical protein LOC100289097 /// similar to FRG1 protein (FSHD region gene 1 protein)
243483_at	TRPM8	2q37.1	transient receptor potential cation channel, subfamily M, member 8
243943_x_at	C6orf52	6p24.2	chromosome 6 open reading frame 52
223170_at	TMEM98	17q11.2	transmembrane protein 98
225599_s_at	C8orf83	8q22.1	chromosome 8 open reading frame 83
227599_at	C3orf59	3q29	chromosome 3 open reading frame 59
232195_at	GPR158	10p12.1	G protein-coupled receptor 158
1553746_a_at	C12orf64	12q21.31	chromosome 12 open reading frame 64
201125_s_at	ITGB5	3q21.2	integrin, beta 5
205122_at	TMEFF1	9q31.1	transmembrane protein with EGF-like and two follistatin-like domains 1
207076_s_at	ASS1	9q34.11	argininosuccinate synthetase 1
213032_at	NFIB	9p22.3	nuclear factor I/B
218613_at	PSD3	8p22	pleckstrin and Sec7 domain containing 3
221942_s_at	GUCY1A3	4q32.1	guanylate cyclase 1, soluble, alpha 3
222116_s_at	TBC1D16	17q25.3	TBC1 domain family, member 16
226420_at	MECOM	3q26.2	MDS1 and EVI1 complex locus
242344_at	GABRB2	5q34	gamma-aminobutyric acid (GABA) A receptor, beta 2
203304_at	BAMBI	10p12.1	BMP and activin membrane-bound inhibitor homolog (Xenopus laevis)
204653_at	TFAP2A	6p24.3	transcription factor AP-2 alpha (activating enhancer binding protein 2 alpha)
205542_at	STEAP1	7q21.13	six transmembrane epithelial antigen of the prostate 1
220129_at	SOHLH2	13q13.3	spermatogenesis and oogenesis specific basic helix-loop-helix 2
227235_at	GUCY1A3	4q32.1	guanylate cyclase 1, soluble, alpha 3
238784_at	DPY19L2	7p14.2	dpy-19-like 2 (C. elegans)
235700_at	CT45A5	Xq26.3	cancer/testis antigen family 45, member A5
201681_s_at	DLG5	10q22.3	discs, large homolog 5 (Drosophila)
207666_x_at	SSX3	Xp11.23	synovial sarcoma, X breakpoint 3
214044_at	RYR2	1q43	ryanodine receptor 2 (cardiac)
223582_at	GPR98	5q14.3	G protein-coupled receptor 98
227955_s_at	EFNA5	5q21.3	ephrin-A5
228919_at	---	---	---
201540_at	FHL1	Xq26.3	four and a half LIM domains 1
201976_s_at	MYO10	5p15.1	myosin X
202016_at	MEST	7q32.2	mesoderm specific transcript homolog (mouse)
203355_s_at	PSD3	8p22	pleckstrin and Sec7 domain containing 3
205347_s_at	TMSB15A	Xq22.1	thymosin beta 15a
206508_at	CD70	19p13.3	CD70 molecule
212190_at	SERPINE2	2q36.1	serpin peptidase inhibitor, clade E (nexin, plasminogen activator inhibitor type 1), member 2
212458_at	SPRED2	2p14	sprouty-related, EVH1 domain containing 2
219855_at	NUDT11	Xp11.22	nudix (nucleoside diphosphate linked moiety X)-type motif 11
233092_s_at	LOC100271840	---	hypothetical LOC100271840
237563_s_at	---	---	---
204086_at	PRAME	22q11.22	preferentially expressed antigen in melanoma

205348_s_at	DYNC1I1	7q21.3	dynein, cytoplasmic 1, intermediate chain 1
214603_at	MAGEA2	Xq28	melanoma antigen family A, 2 /// melanoma antigen family A, 2B
219631_at	LRP12	8q22.3	low density lipoprotein-related protein 12
235947_at	---	---	---
238497_at	TMEM136	11q23.3	transmembrane protein 136
203386_at	TBC1D4	13q22.2	TBC1 domain family, member 4
204469_at	PTPRZ1	7q31.32	protein tyrosine phosphatase, receptor-type, Z polypeptide 1
210999_s_at	GRB10	7p12.1	growth factor receptor-bound protein 10
1567912_s_at	CT45A1	Xq26.3	cancer/testis antigen family 45, member A1 /// cancer/testis antigen family 45, member A2 /// cancer/testis antigen family 45, member A3 /// cancer/testis antigen family 45, member A4 /// cancer/testis antigen family 45, member A5 /// cancer/testis antigen family 45, member A6 /// hypothetical protein LOC100133581
206953_s_at	LPHN2	1p31.1	latrophilin 2
212354_at	SULF1	8q13.2	sulfatase 1
219010_at	C1orf106	1q32.1	chromosome 1 open reading frame 106
223948_s_at	TMPRSS3	21q22.3	transmembrane protease, serine 3
226446_at	HES6	2q37.3	hairy and enhancer of split 6 (Drosophila)
228347_at	SIX1	14q23.1	SIX homeobox 1
1564220_a_at	---	---	---
205709_s_at	CDS1	4q21.23	CDP-diacylglycerol synthase (phosphatidate cytidylyltransferase) 1
209655_s_at	TMEM47	Xp21.1	transmembrane protein 47
224507_s_at	MGC12916	---	hypothetical protein MGC12916
225056_at	SIPA1L2	1q42.2	signal-induced proliferation-associated 1 like 2
204365_s_at	REEP1	2p11.2	receptor accessory protein 1
206626_x_at	SSX1	Xp11.23	synovial sarcoma, X breakpoint 1
206834_at	HBD	11p15.4	hemoglobin, delta
209656_s_at	TMEM47	Xp21.1	transmembrane protein 47
223794_at	ARMC4	10p12.1	armadillo repeat containing 4
229057_at	SCN2A	2q24.3	sodium channel, voltage-gated, type II, alpha subunit
226225_at	MCC	5q22.2	mutated in colorectal cancers
208670_s_at	EID1	---	EP300 interacting inhibitor of differentiation 1
227109_at	CYP2R1	11p15.2	cytochrome P450, family 2, subfamily R, polypeptide 1
238452_at	FCRLB	1q23.3	Fc receptor-like B
242100_at	CHSY3	5q23.3	chondroitin sulfate synthase 3
216215_s_at	RBM9	---	RNA binding motif protein 9
212958_x_at	PAM	5q21.1	peptidylglycine alpha-amidating monooxygenase
239108_at	FAR2	12p11.22	Fatty acyl CoA reductase 2
204602_at	DKK1	10q21.1	dickkopf homolog 1 (Xenopus laevis)
207981_s_at	ESRRG	1q41	estrogen-related receptor gamma
204167_at	BTD	3p25.1	biotinidase
225010_at	CCDC6	10q21.2	coiled-coil domain containing 6
227153_at	IMMP2L	7q31.1	IMP2 inner mitochondrial membrane peptidase-like (S. cerevisiae)
205051_s_at	KIT	4q12	v-kit Hardy-Zuckerman 4 feline sarcoma viral oncogene homolog
1554609_at	---	---	---
1556389_at	CNPY3	6p21.1	canopy 3 homolog (zebrafish)
209360_s_at	RUNX1	21q22.12	runt-related transcription factor 1
230285_at	SVIP	11p14.3	small VCP/p97-interacting protein
220850_at	MORC1	3q13.13	MORC family CW-type zinc finger 1
1552519_at	ACVR1C	2q24.1	activin A receptor, type IC
201287_s_at	SDC1	2p24.1	syndecan 1
220470_at	BET1L	11p15.5	blocked early in transport 1 homolog (S. cerevisiae)-like
238026_at	RPL35A	3q29	ribosomal protein L35a
203725_at	GADD45A	1p31.3	growth arrest and DNA-damage-inducible, alpha
219410_at	TMEM45A	3q12.2	transmembrane protein 45A
218806_s_at	VAV3	1p13.3	vav 3 guanine nucleotide exchange factor
238461_at	EIF4E3	3p13	eukaryotic translation initiation factor 4E family member 3
203038_at	PTPRK	6q22.33	protein tyrosine phosphatase, receptor type, K
209961_s_at	HGF	7q21.11	hepatocyte growth factor (hepapoietin A; scatter factor)
214117_s_at	BTD	3p25.1	biotinidase
206176_at	BMP6	6p24.3	bone morphogenetic protein 6
230175_s_at	---	---	---
210997_at	HGF	7q21.11	hepatocyte growth factor (hepapoietin A; scatter factor)
225946_at	RASSF8	12p12.1	Ras association (RalGDS/AF-6) domain family (N-terminal) member 8
224856_at	FKBP5	6p21.31	FK506 binding protein 5
210998_s_at	HGF	7q21.11	hepatocyte growth factor (hepapoietin A; scatter factor)
219572_at	CADPS2	7q31.32	Ca++-dependent secretion activator 2
224840_at	FKBP5	6p21.31	FK506 binding protein 5
226884_at	LRRN1	3p26.2	leucine rich repeat neuronal 1
209966_x_at	ESRRG	1q41	estrogen-related receptor gamma
220615_s_at	FAR2	12p11.22	fatty acyl CoA reductase 2
226961_at	PRR15	7p14.3	proline rich 15
225058_at	GPR108	19p13.3	G protein-coupled receptor 108
233609_at	PTPRK	6q22.33	Protein tyrosine phosphatase, receptor type, K
202336_s_at	PAM	5q21.1	peptidylglycine alpha-amidating monooxygenase
210830_s_at	PON2	7q21.3	paraoxonase 2
219743_at	HEY2	6q22.31	hairy/enhancer-of-split related with YRPW motif 2

222921_s_at	HEY2	6q22.31	hairy/enhancer-of-split related with YRPW motif 2
215059_at	---	---	---
219628_at	ZMAT3	3q26.32	zinc finger, matrin type 3
217053_x_at	ETV1	7p21.2	ets variant 1
231130_at	FKBP7	2q31.2	FK506 binding protein 7
226692_at	SERF2	15q15.3	small EDRK-rich factor 2
230428_at	---	---	---
209960_at	HGF	7q21.11	hepatocyte growth factor (hepapoietin A; scatter factor)
214879_x_at	USF2	19q13.12	upstream transcription factor 2, c-fos interacting
210812_at	XRCC4	5q14.2	X-ray repair complementing defective repair in Chinese hamster cells 4
213324_at	SRC	20q11.23	v-src sarcoma (Schmidt-Ruppin A-2) viral oncogene homolog (avian)
220595_at	PDZRN4	12q12	PDZ domain containing ring finger 4
209583_s_at	CD200	3q13.2	CD200 molecule
204917_s_at	MLLT3	9p21.3	myeloid/lymphoid or mixed-lineage leukemia (trithorax homolog, Drosophila); translocated to, 3
222451_s_at	ZDHHC9	Xq26.1	zinc finger, DHHC-type containing 9
207399_at	BFSP2	3q22.1	beaded filament structural protein 2, phakinin
220120_s_at	EPB41L4A	5q22.2	erythrocyte membrane protein band 4.1 like 4A
221933_at	NLGN4X	Xp22.31	neuroligin 4, X-linked
203349_s_at	ETV5	3q27.2	ets variant 5
203769_s_at	STS	Xp22.31	steroid sulfatase (microsomal), isozyme S
225540_at	MAP2	2q34	microtubule-associated protein 2
221297_at	GPRC5D	12p13.1	G protein-coupled receptor, family C, group 5, member D
228218_at	---	---	---
229084_at	CNTN4	3p26.3	contactin 4
205073_at	CYP2J2	1p32.1	cytochrome P450, family 2, subfamily J, polypeptide 2
202728_s_at	LTBP1	2p22.3	latent transforming growth factor beta binding protein 1
205578_at	ROR2	9q22.31	receptor tyrosine kinase-like orphan receptor 2
230560_at	STXBP6	14q12	syntaxin binding protein 6 (amisyn)
227492_at	---	---	---
210130_s_at	TM7SF2	11q13.1	transmembrane 7 superfamily member 2
222520_s_at	IFT57	3q13.13	intraflagellar transport 57 homolog (Chlamydomonas)
222785_x_at	C11orf1	11q23.1	chromosome 11 open reading frame 1
1560316_s_at	GLCCI1	7p21.3	glucocorticoid induced transcript 1
229244_at	---	---	---
208190_s_at	LSR	19q13.12	lipolysis stimulated lipoprotein receptor
228150_at	SEC16B	1q25.2	SEC16 homolog B (S. cerevisiae)
238646_at	---	---	---
239468_at	MKX	10p12.1	mohawk homeobox
227209_at	CNTN1	12q12	Contactin 1
1569652_at	MLLT3	9p21.3	myeloid/lymphoid or mixed-lineage leukemia (trithorax homolog, Drosophila); translocated to, 3
236646_at	C12orf59	12p13.2	chromosome 12 open reading frame 59
214395_x_at	EEF1D	8q24.3	eukaryotic translation elongation factor 1 delta (guanine nucleotide exchange protein)
218807_at	VAV3	1p13.3	vav 3 guanine nucleotide exchange factor
244787_at	---	---	---
201876_at	PON2	7q21.3	paraoxonase 2
215195_at	PRKCA	17q24.2	protein kinase C, alpha
227342_s_at	MYEOV	11q13.3	myeloma overexpressed (in a subset of t(11;14) positive multiple myelomas)
229319_at	---	---	---
1553871_at	C19orf34	19p13.3	chromosome 19 open reading frame 34
200666_s_at	DNAJB1	19p13.12	DnaJ (Hsp40) homolog, subfamily B, member 1
205990_s_at	WNT5A	3p14.3	wingless-type MMTV integration site family, member 5A
226618_at	FLJ25076	---	probable ubiquitin-conjugating enzyme E2 FLJ25076
218772_x_at	TMEM38B	9q31.2	transmembrane protein 38B
231530_s_at	C11orf1	11q23.1	chromosome 11 open reading frame 1
235494_at	---	---	---
222433_at	ENAH	1q42.12	enabled homolog (Drosophila)
221207_s_at	NBEA	13q13.3	neurobeachin
210946_at	PPAP2A	5q11.2	phosphatidic acid phosphatase type 2A
1563182_at	ACVR1C	2q24.1	activin A receptor, type IC
200730_s_at	PTP4A1	6q12	protein tyrosine phosphatase type IVA, member 1
225496_s_at	SYTL2	11q14.1	synaptotagmin-like 2
228956_at	UGT8	4q26	UDP glycosyltransferase 8
223136_at	AIG1	6q24.2	androgen-induced 1
202011_at	TJP1	15q13.1	tight junction protein 1 (zona occludens 1)
210336_x_at	MZF1	19q13.43	myeloid zinc finger 1
213938_at	ERC2	3p14.3	ELKS/RAB6-interacting/CAST family member 2
231431_s_at	---	---	---
200973_s_at	TSPAN3	15q24.3	tetraspanin 3
1561657_at	---	---	---
1552716_at	SPEF2	5p13.2	sperm flagellar 2
229233_at	NRG3	10q23.1	neuregulin 3
200811_at	CIRBP	19p13.3	cold inducible RNA binding protein
204118_at	CD48	1q23.3	CD48 molecule
203998_s_at	SYT1	12q21.2	synaptotagmin I

212095_s_at	MTUS1	8p22	mitochondrial tumor suppressor 1
203895_at	PLCB4	20p12.3	phospholipase C, beta 4
206701_x_at	EDNRB	13q22.3	endothelin receptor type B
1554833_at	MCTP2	15q26.2	multiple C2 domains, transmembrane 2
223854_at	PCDHB10	5q31.3	protocadherin beta 10
226586_at	ANKS6	9q22.33	ankyrin repeat and sterile alpha motif domain containing 6
212843_at	NCAM1	11q23.2	neural cell adhesion molecule 1
226439_s_at	NBEA	13q13.3	neurobeachin
243109_at	MCTP2	15q26.2	multiple C2 domains, transmembrane 2
231997_at	TBCEL	11q23.3	tubulin folding cofactor E-like
204918_s_at	MLLT3	9p21.3	myeloid/lymphoid or mixed-lineage leukemia (trithorax homolog, Drosophila); translocated to, 3
213425_at	WNT5A	3p14.3	wingless-type MMTV integration site family, member 5A
202729_s_at	LTBP1	2p22.3	latent transforming growth factor beta binding protein 1
226462_at	STXBP6	14q12	syntaxin binding protein 6 (amisyn)
205442_at	MFAP3L	4q33	microfibrillar-associated protein 3-like
214734_at	EXPH5	11q22.3	exophilin 5
225133_at	KLF3	4p14	Kruppel-like factor 3 (basic)
207996_s_at	C18orf1	18p11.21	chromosome 18 open reading frame 1
239252_at	---	---	---
203987_at	FZD6	8q22.3	frizzled homolog 6 (Drosophila)
213415_at	CLIC2	Xq28	chloride intracellular channel 2
227394_at	NCAM1	11q23.2	neural cell adhesion molecule 1
233582_at	LOC115110	---	hypothetical protein LOC115110
210755_at	HGF	7q21.11	hepatocyte growth factor (hepapoietin A; scatter factor)
227306_at	---	---	---
232745_x_at	SPEF2	5p13.2	sperm flagellar 2
204014_at	DUSP4	8p12	dual specificity phosphatase 4
208711_s_at	CCND1	11q13.3	cyclin D1
212724_at	RND3	2q23.3	Rho family GTPase 3
217998_at	LOC100289208	---	hypothetical protein LOC100289208 /// pleckstrin homology-like domain, family A, member 1
234996_at	CALCRL	2q32.1	calcitonin receptor-like
231227_at	---	---	---
242523_at	---	---	---
203243_s_at	PDLIM5	4q22.3	PDZ and LIM domain 5
220994_s_at	STXBP6	14q12	syntaxin binding protein 6 (amisyn)
203698_s_at	FRZB	2q32.1	frizzled-related protein
1557673_at	---	---	---
1554997_a_at	PTGS2	1q31.1	prostaglandin-endoperoxide synthase 2 (prostaglandin G/H synthase and cyclooxygenase)
205373_at	CTNNA2	2p12	catenin (cadherin-associated protein), alpha 2
212099_at	RHOB	2p24.1	ras homolog gene family, member B
243433_at	---	---	---
213110_s_at	COL4A5	Xq22.3	collagen, type IV, alpha 5
209708_at	MOXD1	6q23.2	monooxygenase, DBH-like 1
235047_x_at	NACC1	19p13.2	nucleus accumbens associated 1, BEN and BTB (POZ) domain containing
223704_s_at	DMRT2	9p24.3	doublesex and mab-3 related transcription factor 2
231726_at	PCDHB14	5q31.3	protocadherin beta 14
201843_s_at	EFEMP1	2p16.1	EGF-containing fibulin-like extracellular matrix protein 1
213901_x_at	RBM9	---	RNA binding motif protein 9
1553253_at	ASB16	17q21.31	ankyrin repeat and SOCS box-containing 16
209101_at	CTGF	6q23.2	connective tissue growth factor
222365_at	---	---	---
233265_at	---	---	---
238605_at	NOL4	18q12.1	nucleolar protein 4
1558796_a_at	LOC728052	---	Similar to hCG2031213
218885_s_at	GALNT12	9q22.33	UDP-N-acetyl-alpha-D-galactosamine:polypeptide N-acetylgalactosaminyltransferase 12 (GalNAc-T12)
235201_at	---	---	---
243184_at	---	---	---
204271_s_at	EDNRB	13q22.3	endothelin receptor type B
206045_s_at	NOL4	18q12.1	nucleolar protein 4
202973_x_at	FAM13A	4q22.1	family with sequence similarity 13, member A
215428_at	---	---	---
227202_at	CNTN1	12q12	Contactin 1
212104_s_at	RBM9	---	RNA binding motif protein 9
210942_s_at	ST3GAL6	3q12.1	ST3 beta-galactoside alpha-2,3-sialyltransferase 6
235205_at	LOC346887	---	similar to solute carrier family 16 (monocarboxylic acid transporters), member 14
213355_at	ST3GAL6	3q12.1	ST3 beta-galactoside alpha-2,3-sialyltransferase 6
219747_at	C4orf31	4q27	chromosome 4 open reading frame 31
241560_at	---	---	---
227180_at	ELOVL7	5q12.1	ELOVL family member 7, elongation of long chain fatty acids (yeast)
227722_at	RPS23	5q14.2	ribosomal protein S23
230319_at	---	---	---
204321_at	NEO1	15q24.1	neogenin homolog 1 (chicken)

223470_at	PIGM	1q23.2	phosphatidylinositol glycan anchor biosynthesis, class M
207623_at	ABCF2	7q36.1	ATP-binding cassette, sub-family F (GCN20), member 2
226656_at	CRTAP	3p22.3	cartilage associated protein
235168_at	PIGM	1q23.2	phosphatidylinositol glycan anchor biosynthesis, class M
231851_at	RAVER2	1p31.3	ribonucleoprotein, PTB-binding 2
204824_at	ENDOG	9q34.11	endonuclease G
203215_s_at	MYO6	6q14.1	myosin VI
210157_at	C19orf2	19q12	chromosome 19 open reading frame 2
201061_s_at	STOM	9q33.2	stomatin
207266_x_at	RBMS1	2q24.2	RNA binding motif, single stranded interacting protein 1
221524_s_at	RRAGD	6q15	Ras-related GTP binding D
217820_s_at	ENAH	1q42.12	enabled homolog (Drosophila)
223042_s_at	FUNDC2	Xq28	FUN14 domain containing 2
227503_at	---	---	---
225269_s_at	RBMS1	2q24.2	RNA binding motif, single stranded interacting protein 1
227126_at	---	---	---
227903_x_at	C19orf20	19p13.3	chromosome 19 open reading frame 20
225823_at	C19orf70	19p13.3	chromosome 19 open reading frame 70
209147_s_at	PPAP2A	5q11.2	phosphatidic acid phosphatase type 2A
202912_at	ADM	11p15.4	adrenomedullin
224802_at	NDFIP2	13q31.1	Nedd4 family interacting protein 2
208745_at	ATP5L	11q23.3	ATP synthase, H+ transporting, mitochondrial F0 complex, subunit G
205208_at	ALDH1L1	3q21.3	aldehyde dehydrogenase 1 family, member L1
225745_at	LRP6	12p13.2	low density lipoprotein receptor-related protein 6
212463_at	CD59	11p13	CD59 molecule, complement regulatory protein
236009_at	PERP	6q23.3	PERP, TP53 apoptosis effector
223823_at	KCNMB2	3q26.32	potassium large conductance calcium-activated channel, subfamily M, beta member 2
202946_s_at	BTBD3	20p12.2	BTB (POZ) domain containing 3
231131_at	FAM133A	Xq21.32	family with sequence similarity 133, member A
1557167_at	HCG11	6p22.2	HLA complex group 11
225265_at	RBMS1	2q24.2	RNA binding motif, single stranded interacting protein 1
203748_x_at	RBMS1	2q24.2	RNA binding motif, single stranded interacting protein 1
229313_at	ANO5	11p14.3	anoctamin 5
209368_at	EPHX2	8p21.2	epoxide hydrolase 2, cytoplasmic
218868_at	ACTR3B	7q36.1	ARP3 actin-related protein 3 homolog B (yeast)
221523_s_at	RRAGD	6q15	Ras-related GTP binding D
1560115_a_at	KIAA1217	10p12.2	KIAA1217
222735_at	TMEM38B	9q31.2	transmembrane protein 38B
236622_at	PIGM	1q23.2	phosphatidylinositol glycan anchor biosynthesis, class M
201482_at	QSOX1	1q25.2	quiescin Q6 sulfhydryl oxidase 1
219532_at	ELOVL4	6q14.1	elongation of very long chain fatty acids (FEN1/Elo2, SUR4/Elo3, yeast)-like 4
202242_at	TSPAN7	Xp11.4	tetraspanin 7
236752_at	---	---	---
202648_at	---	---	---
225698_at	C5orf26	---	chromosome 5 open reading frame 26
227370_at	FAM171B	2q32.1	family with sequence similarity 171, member B
228375_at	IGSF11	3q13.32	immunoglobulin superfamily, member 11
204944_at	PTPRG	3p14.2	protein tyrosine phosphatase, receptor type, G
202893_at	UNC13B	9p13.3	unc-13 homolog B (C. elegans)
236104_at	HNRPLL	2p22.1	heterogeneous nuclear ribonucleoprotein L-like
220253_s_at	LRP12	8q22.3	low density lipoprotein-related protein 12
208712_at	CCND1	11q13.3	cyclin D1
225112_at	ABI2	2q33.2	abl-interactor 2
220062_s_at	MAGEC2	Xq27.2	melanoma antigen family C, 2
235228_at	CCDC85A	2p16.1	coiled-coil domain containing 85A
223125_s_at	C1orf21	1q25.3	chromosome 1 open reading frame 21
206609_at	MAGEC1	Xq27.2	melanoma antigen family C, 1
225004_at	TMEM101	17q21.31	transmembrane protein 101
244532_x_at	---	---	---
209737_at	MAGI2	7q21.11	membrane associated guanylate kinase, WW and PDZ domain containing 2
210145_at	PLA2G4A	1q31.1	phospholipase A2, group IVA (cytosolic, calcium-dependent)
225922_at	FNIP2	4q32.1	folliculin interacting protein 2
209631_s_at	GPR37	7q31.33	G protein-coupled receptor 37 (endothelin receptor type B-like)
223614_at	MMP16	8q21.3	matrix metallopeptidase 16 (membrane-inserted)
235014_at	LOC147727	---	hypothetical LOC147727
231856_at	KIAA1244	6q23.3	KIAA1244
1568597_at	LOC646762	---	hypothetical LOC646762
214829_at	AASS	7q31.32	aminoadipate-semialdehyde synthase
1554438_at	KIAA1217	10p12.2	KIAA1217
211098_x_at	TMCO1	1q24.1	transmembrane and coiled-coil domains 1
1557169_x_at	HCG11	6p22.2	HLA complex group 11
222896_at	TMEM38A	19p13.11	transmembrane protein 38A
210655_s_at	FOXO3	6q21	forkhead box O3 /// forkhead box O3B pseudogene
1557765_at	LOC643401	---	hypothetical protein LOC643401
220755_s_at	C6orf48	HSCHR6_MHC_QBL	chromosome 6 open reading frame 48
229435_at	GLIS3	9p24.2	GLIS family zinc finger 3

214001_x_at	---	---	---
204485_s_at	TOM1L1	17q22	target of myb1 (chicken)-like 1
231807_at	KIAA1217	10p12.2	KIAA1217
243278_at	---	---	---
205110_s_at	FGF13	Xq27.1	fibroblast growth factor 13
205968_at	KCNS3	2p24.2	potassium voltage-gated channel, delayed-rectifier, subfamily S, member 3
225600_at	C8orf83	8q22.1	chromosome 8 open reading frame 83
244359_s_at	---	---	---
243918_at	---	---	---
207086_x_at	GAGE1	Xp11.23	G antigen 1 /// G antigen 12C /// G antigen 12D /// G antigen 12E /// G antigen 12F /// G antigen 12G /// G antigen 12H /// G antigen 12I /// G antigen 12J /// G antigen 2A /// G antigen 2C /// G antigen 2D /// G antigen 2E /// G antigen 4 /// G antigen 5 /// G antigen 6 /// G antigen 7 /// G antigen 8
219195_at	PPARGC1A	4p15.2	peroxisome proliferator-activated receptor gamma, coactivator 1 alpha
235764_at	---	---	---
