# Supplementary material for: Identification of Pluripotent and Adult Stem Cell Genes Unrelated to Cell Cycle and Associated with Poor Prognosis in Multiple Myeloma
Source: PLoS One. 2012 Jul 31;7(7):e42161. doi: 10.1371/journal.pone.0042161 (PMC3409163; doi:10.1371/journal.pone.0042161)
Supplement: Table S3 — The 50 genes with pronostic value in HM and UAMS-TT2 cohorts. (PDF) [file pone.0042161.s005.pdf]

Table S3. The 50 genes with pronostic value in HM and UAMS-TT2 cohorts

Probe sets	Name	Cytoband	Location	Type(s)	Maxstat cut-point	% of patients overexpressing the gene	P (log-rank)	Hazard ratio	Prognostic value	Categorie	Description
**Cancer testis antigen**											
211674_x_at	CTAG1A	Xq28	Cytoplasm	other	36	32.04	0.013	2.129	BAD	Cancer testis	cancer/testis antigen 1A /// cancer/testis antigen 1B
207663_x_at	GAGE3	Xp11.23	unknown	other	221	21.36	0.019	2.122	BAD	Cancer testis	G antigen 3
208235_x_at	GAGE12F	Xp11.23	unknown	other	70	30.10	0.001	2.659	BAD	Cancer testis	G antigen 12F /// G antigen 12G /// G antigen 12I /// G antigen 7
214612_x_at	MAGEA6	Xq28	unknown	other	1211	18.45	0.003	2.486	BAD	Cancer testis	melanoma antigen family A, 6
214642_x_at	MAGEA5	Xq28	unknown	other	309	17.48	0.009	2.329	BAD	Cancer testis	melanoma antigen family A, 5
**Membrane proteins**											
1552519_at	ACVR1C	2q24.1	Plasma Membrane	kinase	419	74.76	0.030	0.506	GOOD	Membran proteins	activin A receptor, type IC
203304_at	BAMBI	10p12.1	Plasma Membrane	other	576	10.19	0.021	2.536	BAD	Membran proteins	BMP and activin membrane-bound inhibitor homolog (Xenopus laevis)
203627_at	IGF1R	15q26.3	Plasma Membrane	transmembra	150	25.24	0.004	2.372	BAD	Membran proteins	insulin-like growth factor 1 receptor
205433_at	BCHE	3q26.1	Plasma Membrane	enzyme	131	32.52	0.029	1.942	BAD	Membran proteins	butyrylcholinesterase
207307_at	HTR2C	Xq23	Plasma Membrane	G-protein co	284	14.08	0.007	2.576	BAD	Membran proteins	5-hydroxytryptamine (serotonin) receptor 2C
207717_s_at	PKP2	12p11.21	Plasma Membrane	other	398	27.18	0.003	2.443	BAD	Membran proteins	plakophilin 2
213194_at	ROBO1	3p12.2	Plasma Membrane	transmembra	638	29.61	0.000	2.931	BAD	Membran proteins	roundabout, axon guidance receptor, homolog 1 (Drosophila)
220993_s_at	GPR63	6q16.1	Plasma Membrane	G-protein co	180	28.16	0.017	2.075	BAD	Membran proteins	G protein-coupled receptor 63
223993_s_at	CNIH4	1q42.11	Plasma Membrane	other	3817	60.19	0.043	1.936	BAD	Membran proteins	cornichon homolog 4 (Drosophila)
**Metabolism**											
202620_s_at	PLOD2	3q24	Cytoplasm	enzyme	1184	15.53	0.022	2.196	BAD	Metabolism	procollagen-lysine, 2-oxoglutarate 5-dioxygenase 2
205354_at	GAMT	19p13.3	Cytoplasm	enzyme	36	81.55	0.000	0.230	GOOD	Metabolism	guanidinoacetate N-methyltransferase
210130_s_at	TM7SF2	11q13.1	Cytoplasm	enzyme	560	63.11	0.004	0.402	GOOD	Metabolism	transmembrane 7 superfamily member 2
213249_at	FBXL7	5p15.1	Cytoplasm	enzyme	325	12.62	0.030	2.152	BAD	Metabolism	F-box and leucine-rich repeat protein 7
217771_at	GOLM1	9q21.33	Cytoplasm	other	631	10.19	0.013	2.462	BAD	Metabolism	golgi membrane protein 1
219733_s_at	SLC27A5	19q13.43	Cytoplasm	transporter	211	14.56	0.015	2.479	BAD	Metabolism	solute carrier family 27 (fatty acid transporter), member 5
219855_at	NUDT11	Xp11.22	Cytoplasm	phosphatase	468	11.65	0.000	4.633	BAD	Metabolism	nudix (nucleoside diphosphate linked moiety X)-type motif 11
217894_at	KCTD3	1q41	unknown	ion channel	522	22.82	0.031	1.981	BAD	Metabolism	potassium channel tetramerisation domain containing 3
**Transcription**											
209511_at	POLR2F	22q13.1	Nucleus	enzyme	1615	13.59	0.007	2.593	BAD	transcription	polymerase (RNA) II (DNA directed) polypeptide F
212151_at	PBX1	1q23.3	Nucleus	transcription	777	10.68	0.001	3.078	BAD	Transcription	pre-B-cell leukemia homeobox 1
213032_at	NFIB	9p22.3	Nucleus	transcription	11	52.43	0.007	0.431	GOOD	Transcription	nuclear factor I/B
**Translation**											
228523_at	NANOS1	10q26.11	Cytoplasm	other	170	52.91	0.002	2.959	BAD	Translation	nanos homolog 1 (Drosophila)
200811_at	CIRBP	19p13.3	Nucleus	translation re	6154	88.83	0.015	0.413	GOOD	Translation	cold inducible RNA binding protein
**Development**											
216194_s_at	TBCB	19q13.12	Cytoplasm	other	1507	42.23	0.011	2.196	BAD	Development	tubulin folding cofactor B
231807_at	KIAA1217	10p12.2	Cytoplasm	other	1499	22.33	0.015	0.298	GOOD	Development	KIAA1217
214608_s_at	EYA1	8q13.3	Nucleus	phosphatase	316	11.65	0.005	2.677	BAD	Development	eyes absent homolog 1 (Drosophila)
228654_at	SPIN4	Xq11.1	unknown	other	1393	10.19	0.001	3.311	BAD	Development	spindlin family, member 4
**Cytosqueleton**											
205442_at	MFAP3L	4q33	unknown	other	1624	50.97	0.001	0.340	GOOD	Cytosqueleton	microfibrillar-associated protein 3-like
224823_at	MYLK	3q21.1	Cytoplasm	kinase	743	19.90	0.009	2.303	BAD	cytosqueleton	myosin light chain kinase
**Cell signaling**											
204066_s_at	AGAP1	2q37.2	Cytoplasm	enzyme	518	16.50	0.001	2.743	BAD	Cell signaling	ArfGAP with GTPase domain, ankyrin repeat and PH domain 1
**Chromatin assembly**											
204749_at	NAP1L3	Xq21.32	Nucleus	other	277	12.14	0.000	4.011	BAD	Chromatin assemblye	nucleosome assembly protein 1-like 3
**Cell death**											
204364_s_at	REEP1	2p11.2	Cytoplasm	other	180	19.42	0.005	2.403	BAD	Cell Death	receptor accessory protein 1
**Immune response**											
225646_at	CTSC	11q14.2	Cytoplasm	peptidase	591	36.41	0.008	2.235	BAD	Immune response	cathepsin C
**Other**											
223253_at	EPDR1	7p14.1	Nucleus	other	493	32.52	0.002	2.493	BAD	Other	ependymin related protein 1 (zebrafish)
223530_at	TDRKH	1q21.3	Cytoplasm	other	297	10.68	0.048	2.141	BAD	Other	tudor and KH domain containing
224129_s_at	DPY30	2p22.3	Nucleus	other	3535	18.45	0.003	2.776	BAD	Other	dpy-30 homolog (C. elegans)
1554057_at	LOC645676	---	unknown	other	943	51.46	0.008	2.473	BAD	Other	hypothetical LOC645676
1568597_at	LOC646762	---	unknown	other	1563	23.79	0.014	2.144	BAD	Other	hypothetical LOC646762
1568780_at	LOC649305	---	unknown	other	253	16.99	0.041	1.964	BAD	Other	hypothetical LOC649305
204521_at	C12orf24	12q24.11	unknown	other	933	11.17	0.048	2.069	BAD	Other	chromosome 12 open reading frame 24
219010_at	C1orf106	1q32.1	unknown	other	150	83.98	0.050	3.761	BAD	Other	chromosome 1 open reading frame 106
219061_s_at	LAGE3	Xq28	unknown	other	1272	74.27	0.013	3.083	BAD	Other	L antigen family, member 3
219260_s_at	C17orf81	17p13.1	unknown	other	1085	11.17	0.041	2.285	BAD	Other	chromosome 17 open reading frame 81
231131_at	FAM133A	Xq21.32	unknown	other	2658	14.56	0.027	2.189	BAD	Other	family with sequence similarity 133, member A
240185_at	LOC1001477	---	unknown	other	206	10.19	0.001	3.288	BAD	Other	hypothetical LOC100147773
64900_at	FLJ22167	---	unknown	other	293	43.69	0.015	2.134	BAD	Other	hypothetical protein FLJ22167
