# Supplementary material for: Identification of Pluripotent and Adult Stem Cell Genes Unrelated to Cell Cycle and Associated with Poor Prognosis in Multiple Myeloma
Source: PLoS One. 2012 Jul 31;7(7):e42161. doi: 10.1371/journal.pone.0042161 (PMC3409163; doi:10.1371/journal.pone.0042161)
Supplement: Table S4 — The 37 stem cell myeloma genes. (PDF) [file pone.0042161.s006.pdf]

Table S4. The 37 stem cell myeloma genes

Gene name and gene ontology annotation	Prognostic value	Probe set	Cytoband	Location	Type(s)	% of patients overexpressing the gene	*P*-value (log-rank)	Hazard ratio	Description
**Metabolism**									
PLOD2	BAD	202620_s_at	3q24	Cytoplasm	enzyme	15.5	0.022	2.196	procollagen-lysine, 2-oxoglutarate 5-dioxygenase 2
GAMT	GOOD	205354_at	19p13.3	Cytoplasm	enzyme	81.6	0.000	0.230	guanidinoacetate N-methyltransferase
TM7SF2	GOOD	210130_s_at	11q13.1	Cytoplasm	enzyme	63.1	0.004	0.402	transmembrane 7 superfamily member 2
FBXL7	BAD	213249_at	5p15.1	Cytoplasm	enzyme	12.6	0.030	2.152	F-box and leucine-rich repeat protein 7
GOLM1	BAD	217771_at	9q21.33	Cytoplasm	other	10.2	0.013	2.462	golgi membrane protein 1
KCTD3	BAD	217894_at	1q41	unknown	ion channel	22.8	0.031	1.981	potassium channel tetramerisation domain containing 3
SLC27A5	BAD	219733_s_at	19q13.43	Cytoplasm	transporter	14.6	0.015	2.479	solute carrier family 27 (fatty acid transporter), member 5
NUDT11	BAD	219855_at	Xp11.22	Cytoplasm	phosphatase	11.7	0.000	4.633	nudix (nucleoside diphosphate linked moiety X)-type motif 11
**Membrane proteins**									
BAMBI	BAD	203304_at	10p12.1	Plasma Mem	other	10.2	0.021	2.536	BMP and activin membrane-bound inhibitor homolog (Xenopus laevis)
IGF1R	BAD	203627_at	15q26.3	Plasma Mem	transmembra	25.2	0.004	2.372	insulin-like growth factor 1 receptor
BCHE	BAD	205433_at	3q26.1	Plasma Mem	enzyme	32.5	0.029	1.942	butyrylcholinesterase
PKP2	BAD	207717_s_at	12p11.21	Plasma Mem	other	27.2	0.003	2.443	plakophilin 2
ROBO1	BAD	213194_at	3p12.2	Plasma Mem	transmembra	29.6	0.000	2.931	roundabout, axon guidance receptor, homolog 1 (Drosophila)
CNIH4	BAD	223993_s_at	1q42.11	Plasma Mem	other	60.2	0.043	1.936	cornichon homolog 4 (Drosophila)
**Transcription**									
POLR2F	BAD	209511_at	22q13.1	Nucleus	enzyme	13.6	0.007	2.593	polymerase (RNA) II (DNA directed) polypeptide F
PBX1	BAD	212151_at	1q23.3	Nucleus	transcription	10.7	0.001	3.078	pre-B-cell leukemia homeobox 1
NFIB	GOOD	213032_at	9p22.3	Nucleus	transcription	52.4	0.007	0.431	nuclear factor I/B
**Cytosqueleton**									
MFAP3L	GOOD	205442_at	4q33	unknown	other	51.0	0.001	0.340	microfibrillar-associated protein 3-like
MYLK	BAD	224823_at	3q21.1	Cytoplasm	kinase	19.9	0.009	2.303	myosin light chain kinase
**Development**									
SPIN4	BAD	228654_at	Xq11.1	unknown	other	10.2	0.001	3.311	spindlin family, member 4
KIAA1217	GOOD	231807_at	10p12.2	Cytoplasm	other	22.3	0.015	0.298	KIAA1217
**Translation**									
NANOS1	BAD	228523_at	10q26.11	Cytoplasm	other	52.9	0.002	2.959	nanos homolog 1 (Drosophila)
**Cell signaling**									
AGAP1	BAD	204066_s_at	2q37.2	Cytoplasm	enzyme	16.5	0.001	2.743	ArfGAP with GTPase domain, ankyrin repeat and PH domain 1
**Chromatin assembly**									
NAP1L3	BAD	204749_at	Xq21.32	Nucleus	other	12.1	0.000	4.011	nucleosome assembly protein 1-like 3
**Other**									
LOC645676	BAD	1554057_at	---	unknown	other	51.5	0.008	2.473	hypothetical LOC645676
LOC646762	BAD	1568597_at	---	unknown	other	23.8	0.014	2.144	hypothetical LOC646762
LOC649305	BAD	1568780_at	---	unknown	other	17.0	0.041	1.964	hypothetical LOC649305
C12orf24	BAD	204521_at	12q24.11	unknown	other	11.2	0.048	2.069	chromosome 12 open reading frame 24
C1orf106	BAD	219010_at	1q32.1	unknown	other	84.0	0.050	3.761	chromosome 1 open reading frame 106
LAGE3	BAD	219061_s_at	Xq28	unknown	other	74.3	0.013	3.083	L antigen family, member 3
C17orf81	BAD	219260_s_at	17p13.1	unknown	other	11.2	0.041	2.285	chromosome 17 open reading frame 81
EPDR1	BAD	223253_at	7p14.1	Nucleus	other	32.5	0.002	2.493	ependymin related protein 1 (zebrafish)
TDRKH	BAD	223530_at	1q21.3	Cytoplasm	other	10.7	0.048	2.141	tudor and KH domain containing
DPY30	BAD	224129_s_at	2p22.3	Nucleus	other	18.4	0.003	2.776	dpy-30 homolog (C. elegans)
FAM133A	BAD	231131_at	Xq21.32	unknown	other	14.6	0.027	2.189	family with sequence similarity 133, member A
LOC100147773	BAD	240185_at	---	unknown	other	10.2	0.001	3.288	hypothetical LOC100147773
FLJ22167	BAD	64900_at	---	unknown	other	43.7	0.015	2.134	hypothetical protein FLJ22167
