# Supplementary material for: Identification of Pluripotent and Adult Stem Cell Genes Unrelated to Cell Cycle and Associated with Poor Prognosis in Multiple Myeloma
Source: PLoS One. 2012 Jul 31;7(7):e42161. doi: 10.1371/journal.pone.0042161 (PMC3409163; doi:10.1371/journal.pone.0042161)
Supplement: Table S5 — Clinical characteristics of patients in the 2 groups defined by SCscore. The 206 previously-untreated patients of the HM cohort were treated at the university hospitals of Heidelberg and Montpellier. Patients were separated in 2 groups: low-risk (SCscore ≤ −2.7) and high-risk (SCscore > −2.7) SCscore groups. Data are the percentages of patients within these 2 groups with the indicated clinical or biological parameters. When the percentages were different with a chisquare test (P ≤ .05), data are shown in bold and italic. (PDF) [file pone.0042161.s007.pdf]

	SC score groups	
	^{SC}Score ≤ -2.70 (n = 185)	^{SC}Score> -2.7 (n = 21)
Categories	% of patients in each group	
Age >= 65 yr	18%	29%
IgA subtype	22%	29%
Kappa light chain	63%	75%
Lambda light chain	35%	25%
Non-secreting	2%	0%
β2M<= 3.5 mg/ml	65%	52%
β2M> 5.5 mg/ml	14%	29%
LDH>= 240 IU/liter	23%	25%
**Albumin < 35 g/liter**	**30%**	**55%**
**Hemoglobin< 10 g/dl**	**25%**	**57%**
C-reactive protein >= 5 mg/liter	36%	38%
Bone lesions		
0: normal bone structure	21%	30%
1: osteopenie / osteoporosis	29%	45%
2: osteolyse [1-3]	7%	10%
3: major structural damage [>3]	43%	15%

	^{SC}Score ≤ -2.7 (n = 159)			^{SC}Score >-2.7 (n = 21)		
Staging	I	II	III	I	II	III
Salmon and durie	11%	15%	74%	10%	14%	76%
ISS	50%	35%	15%	29%	43%	28%

Table S5. Clinical characteristics of patients in the 2 groups defined by ^{SC}score

The 206 previously-untreated patients of the HM cohort were treated at the university hospitals of Heidelberg and Montpellier. Patients were separated in 2 groups: low-risk (^{SC}score ≤ -2.7) and high-risk (^{SC}score > -2.7) ^{SC}score groups. Data are the percentages of patients within these 2 groups with the indicated clinical or biological parameters. When the percentages were different with a chisquare test (*P* ≤ .05), data are shown in bold and italic.
